# Impact of DNA extraction on whole genome sequencing analysis for characterization and relatedness of Shiga toxin-producing *Escherichia coli* isolates

**DOI:** 10.1038/s41598-020-71207-3

**Published:** 2020-09-04

**Authors:** Stéphanie Nouws, Bert Bogaerts, Bavo Verhaegen, Sarah Denayer, Denis Piérard, Kathleen Marchal, Nancy H. C. Roosens, Kevin Vanneste, Sigrid C. J. De Keersmaecker

**Affiliations:** 1 Transversal Activities in Applied Genomics, Sciensano, Brussels, Belgium; 2 Department of Information Technology, IDLab, Ghent University, IMEC, Ghent, Belgium; 3 National Reference Laboratory for Shiga Toxin-Producing Escherichia coli (NRL-STEC), Foodborne Pathogens, Sciensano, Brussels, Belgium; 4 Department of Microbiology and Infection Control, National Reference Center for Shiga Toxin-Producing Escherichia coli (NRC-STEC), Vrije Universiteit Brussel (VUB), Universitair Ziekenhuis Brussel (UZ Brussel), Brussels, Belgium; 5 Department of Plant Biotechnology and Bioinformatics, Ghent University, Ghent, Belgium; 6 Department of Genetics, University of Pretoria, Pretoria, South Africa

**Keywords:** Applied microbiology, Microbial genetics, Pathogens, Policy and public health in microbiology, Genomics, Sequencing, Public health, Bioinformatics

## Abstract

Whole genome sequencing (WGS) has proven to be the ultimate tool for bacterial isolate characterization and relatedness determination. However, standardized and harmonized workflows, e.g. for DNA extraction, are required to ensure robust and exchangeable WGS data. Data sharing between (inter)national laboratories is essential to support foodborne pathogen control, including outbreak investigation. This study evaluated eight commercial DNA preparation kits for their potential influence on: (i) DNA quality for Nextera XT library preparation; (ii) MiSeq sequencing (data quality, read mapping against plasmid and chromosome references); and (iii) WGS data analysis, i.e. isolate characterization (serotyping, virulence and antimicrobial resistance genotyping) and phylogenetic relatedness (core genome multilocus sequence typing and single nucleotide polymorphism analysis). Shiga toxin-producing *Escherichia coli* (STEC) was selected as a case study. Overall, data quality and inferred phylogenetic relationships between isolates were not affected by the DNA extraction kit choice, irrespective of the presence of confounding factors such as EDTA in DNA solution buffers. Nevertheless, completeness of STEC characterization was, although not substantially, influenced by the plasmid extraction performance of the kits, especially when using Nextera XT library preparation. This study contributes to addressing the WGS challenges of standardizing protocols to support data portability and to enable full exploitation of its potential.

## Introduction

Whole genome sequencing (WGS) is being or has already been implemented internationally in foodborne pathogen surveillance and outbreak investigations^1–4^. Thanks to its single nucleotide resolution, WGS provides superior isolate characterization and (sub)typing. This enables increased pathogen insight, and linking of human illness to specific foods or production environments with an unprecedented level of confidence^5^.

There are however several challenges to be considered to fully exploit its potential, including the standardization and harmonization of laboratory and computational workflows. Several consortia^6–9^ are currently addressing these bottlenecks in an effort to provide robust and comparable data. Exchange of data between (inter)national laboratories is indeed essential for sound outbreak investigation^10^.

Although a high variety of commercial DNA extraction kits is being used for WGS of foodborne pathogens^11–14^, the literature evaluating the potential impact of DNA extraction workflows on WGS and subsequent isolate characterization and phylogeny is rather limited. Concisely, these studies have demonstrated that DNA extraction kit choice does not influence downstream WGS data analysis of different Gram-negative and -positive pathogens^15,16^, except for salting-out methods that can deplete small plasmids (< 5 kb)^16^. Consequently, virulence and antimicrobial resistance (AMR) genotyping, and subsequent interpretation of strain pathogenicity, can also be affected. Additionally, DNA extraction kit components containing confounding factors such as ethylenediaminetetraacetic acid (EDTA) can diminish or inhibit enzyme activation^17^ required for library preparation and sequencing.

The need therefore exists to more extensively investigate the extent to which DNA extraction kits can affect the outcome of WGS to enable selecting a robust method that allows full characterization and accurate phylogenetic inference of foodborne isolates. In this study, eight frequently used DNA extraction kits (Table 1), referred to as ‘kits’ onwards, were examined to ascertain their effectiveness for extracting DNA from Shiga toxin-producing *Escherichia coli* (STEC) for the purpose of WGS analysis. More specifically, the performance of the kits was evaluated at the level of: (i) DNA quality; (ii) MiSeq sequencing (data quality and read mapping to plasmid and chromosomal references); and (iii) downstream isolate characterization (serotype, antimicrobial resistance (AMR) and virulence genes) and determination of the phylogenetic relationship between isolates.

**Table 1:** Summary of selected DNA extraction kit characteristics and performance.

DNA extraction kit	DNA extraction method	Average completion time (h:min)	Average DNA yield (µg/ml culture) ± s.d.	Average DNA concentration (ng/µl) ± s.d.	DNA purity (average ± s.d.)		Length range of fragments (kb)	Average DIN** ± s.d.	General convenience of protocol	Plasmid extraction performance	Remark
		Sample cost (€)*/total no. reactions			A260/280	A260/230					
DNeasy Blood & Tissue***- Qiagen	Solid-phase (silica column)^21^	2h30	2.40 ± 0.42	23.99 ± 4.24	1.99 ± 0.01	1.78 ± 0.13	[19.88,25.49]	7.07 ± 0.24	+ + +	Impaired	Used by CDC^22^
		€3.76/250									
DNeasy UltraClean Microbial- Qiagen	Solid-phase (bead beating)^23^	2h50	0.78 ± 0.31	28.13 ± 11.04	1.92 ± 0.04	1.87 ± 0.17	[22.50, > 60.00]	8.42 ± 0.33	+ +	Impaired	Bead-beating: also Gram positive bacteria^23^
		€2.87/250									
Easy-DNA Genomic DNA Purification***- Invitrogen	Salting-out^24^	3h30	1.98 ± 0.83	19.82 ± 8.28	1.90 ± 0.01	2.09 ± 0.04	[22.81, > 60.00]	8.96 ± 0.83	+	Impaired	HMW DNA^24^, suitable for NGS^24^
		€2.29/200									
GenElute Bacterial Genomic DNA***- Sigma-Aldrich	Solid-phase (silica column)^25^	2h40	5.53 ± 0.95	33.16 ± 5.70	1.91 ± 0.02	2.19 ± 0.13	[23.37,51.59]	9.13 ± 0.18	+ + +	Good	Especially for Gram-negative bacteria^25^
		€2.59/350									
Genomic-tip 20/G- Qiagen	Solid-phase (anion-exchange)^26^	8h20	1.17 ± 0.29	17.58 ± 4.40	1.84 ± 0.04	1.71 ± 0.19	[53.31, > 60.00]	8.93 ± 0.37	+	Good	HMW DNA ensured^26^
		€10.88/75									
MasterPure Complete DNA Purification***- Lucigen	Salting-out^27^	2h50	2.67 ± 0.53	38.14 ± 7.51	1.87 ± 0.03	1.79 ± 0.21	[58.58, > 60.00]	9.53 ± 0.21	+	Moderate	Recommended by Illumina^28^
		€3.61/200									
NucliSENS miniMag- bioMérieux	Solid-phase (magnetic beads)	1h50	0.72 ± 0.18	7.15 ± 2.50	2.09 ± 0.07	0.76 ± 0.69	[2.46, > 60.00]	7.47 ± 0.70	+ +	Moderate	Possibility of automation
		€7.19/48									
Wizard Genomic DNA Purification***- Promega	Salting-out^29^	2h50	2.06 ± 0.65	22.66 ± 4.55	1.94 ± 0.03	2.10 ± 0.09	> 60.00	9.40 ± 0.34	+	Impaired	High quality DNA ensured^29^
		€0.69/500									

The number of cells used as starting material ranged from 7.36 × 10^8^ ± 8.56 × 10^7^ per ml. All averages and ranges were calculated from the three replicates of the seven DNA extracts. Both DNA concentration and yield are shown since the applied workflows accompanying the kits differed in recommended DNA elution/rehydration volume. General convenience (labor intensity, turn-around time and handling convenience) of the kits is indicated as experienced in this study, from less ( +) to more (+ + +) convenient. Excluding the Genomic-tip 20/G kit with its one-day protocol, all solid-phase procedures were experienced as user-friendly, while the salting-out procedures were experienced as less convenient. Similarly, the plasmid extraction performance as observed in this study from uniquely mapped plasmid reads per million input reads were rated from less (Impaired), medium (Moderate) to best (Good) performing. Fragment lengths are shown as ranges because the TapeStation Genomic DNA ScreenTape only gives exact measurements until 60 kb. Note that only for the NucliSENS miniMag, extra DNA fragments of ~ 2.5 and ~ 5.5 kb were systematically observed across all DNA extracts, resulting in the very large range.

*NGS* next generation sequencing, *HMW* high molecular weight.

*Prices as of August 2020 (excl. TVA, shipping, and handling costs). Prices were calculated from kits with highest throughput. Cost of extra products or materials required but not provided with the kit were not taken into account.

**DIN: DNA Integrity Number, ranging from 1.00 for highly degraded to 10.00 for highly intact DNA^30^.

***DNA was eluted/rehydrated in 10 mM Tris–HCl (pH 8.5) instead of in the EDTA-containing buffer provided with the kit (see Supplementary Table S1 online).

## Results

### Selection of STEC isolates

To evaluate the potential influence of the kit on isolate characterization and relatedness determination, seven isolates were selected (Table 2), including outbreak^18^ (i.e. related) and non-outbreak (i.e. unrelated) isolates. The isolates originated from different matrices (human feces, food samples and a carcass swab), and were previously conventionally serotyped as O157:H7 containing the pO157 plasmid^19^, and O113:H21 containing the pO113 plasmid^20^. The seven isolates were used to test eight kits (Table 1). For four kits, the impact of the presence of EDTA in their DNA elution/rehydration buffers on WGS analysis was evaluated (Supplementary Table S1 online).

**Table 2:** Characteristics of the selected isolates.

TIAC reference	Outbreak/non-outbreak	Origin	Characterization results with conventional methods						Characterization results with WGS analyses	
			Serotype	*stx1*	*stx2*	*eae*	*ehxA*	AMR phenotype	Serotype	AMR genes identified with all kits
TIAC1151	O	Beef	O157:H7	1	1	1	1	Susceptible	O157:H7^θ^	*–*
TIAC1152	O	Beef	O157:H7	1	1	1	1	Susceptible	O157:H7	*–*
TIAC1153	N	Swab carcass bovine	O157:H7	1	1	1	1	AMP KAN, STR SUL TET TMP	O157:H7	*blaTEM-Ib* *aph(3")-Ib*, *aph(3′)-Ia*, and *aph(6)-Id* *sul2* *tet(A)* *dfrA8*
TIAC1165	O	Human feces	O157:H7	1	1	1	1	Susceptible	O157:H7_o_	*–*
TIAC1169	O	Human feces	O157:H7	1	1	1	1	Susceptible	O157:H7*	*–*
TIAC1638	N	Human feces	O157:H7	1	1	1	1	Not tested	O157:H7	*–*
TIAC1660	N	Human feces	O113:H21	0	1	0	1	Not tested	O113:H21*	*–*

Results on STEC serotyping, AMR susceptibility (disc diffusion), and presence of virulence genes assessed previously^18^ are shown (for food isolates according to ISO/TS13136:2012). Gene presence is indicated with ‘1’, absence with ‘0’. The O- and H-type, and AMR genotype determined with WGS for each sample are indicated. Obtained serotypes and AMR genotypes per sample were independent from the applied kit. AMR gene names refer to the ResFinder^31,32^ database. When no AMR genes were detected, this is indicated as “–”. No ambiguities with regard to H-typing were observed. Exceptions in O-typing are represented with a symbol:

^θ^Ambiguous O-typing was retrieved when processed with the DNeasy Blood & Tissue kit, in one of three sequencing run replicates (allele 201 was not called with BLAST + for both the O157-encoding *wzx* and *wzy* genes), but could be resolved in the other two sequencing replicates.

_o_Ambiguous O-typing was retrieved when processed with the DNeasy UltraClean Microbial kit, in two of three sequencing run replicates (allele 201 was not called with BLAST + for both the O157-encoding *wzx* and *wzy* genes), but could be resolved in one of the three sequencing replicates.

*Ambiguous O-typing was retrieved when processed with the Genomic-tip 20/G kit (TIAC1169: allele 201 was not called with BLAST + for both the O157-encoding *wzx* and *wzy* genes; TIAC1660: allele 115 was not called with BLAST + for gene *wzx* encoding the O113-genotype).

*AMP* ampicillin, *KAN* kanamycin, *STR* streptomycin, *SUL* sulphonamides, *TET* tetracycline, *TMP* trimethoprim.

### Extracted DNA quality control checks

The yield and concentration, purity, and integrity of the extracted DNA were evaluated (Table 1). All kits yielded sufficient DNA amounts and concentrations (> 1 ng in 5 µl) to perform Nextera XT library preparation, irrespective of EDTA presence in DNA solution buffers (Supplementary Table S1 online). DNA purity differed significantly between the eight kits (Kruskal–Wallis, n: 168, α: 0.05, p-value: 3.33 × 10^–6^), with the highest purity obtained for all DNA extracts of the GenElute Bacterial Genomic DNA (gDNA), Wizard gDNA Purification and Easy-DNA gDNA Purification kits. Removing EDTA and other confounding factors using the gDNA Clean & Concentrator kit after DNeasy Blood & Tissue sample preparation, also increased DNA purity (Supplementary Table S1 online). When using the MasterPure Complete DNA extraction and DNeasy UltraClean Microbial kits, higher DNA yield and purity, respectively, could be obtained over time through increased experience with the protocol. An additional control check with PCR (Supplementary Table S2 online) was performed on all DNA extracts of isolates TIAC1165 and TIAC1660, targeting an *E. coli* housekeeping gene (*uidA*) and plasmid-specific genes (*ehxA* (pO157 and pO113) and *saa* (pO113)), indicating that all DNA extracts contained *E. coli* DNA and that all tested kits had the potential to extract plasmid DNA (Supplementary Table S3 online), irrespective of the plasmid type (pO113 in TIAC1660, or pO157 in the other isolates).

### Advanced quality control checks of the sequencing data

**Figure 1:**
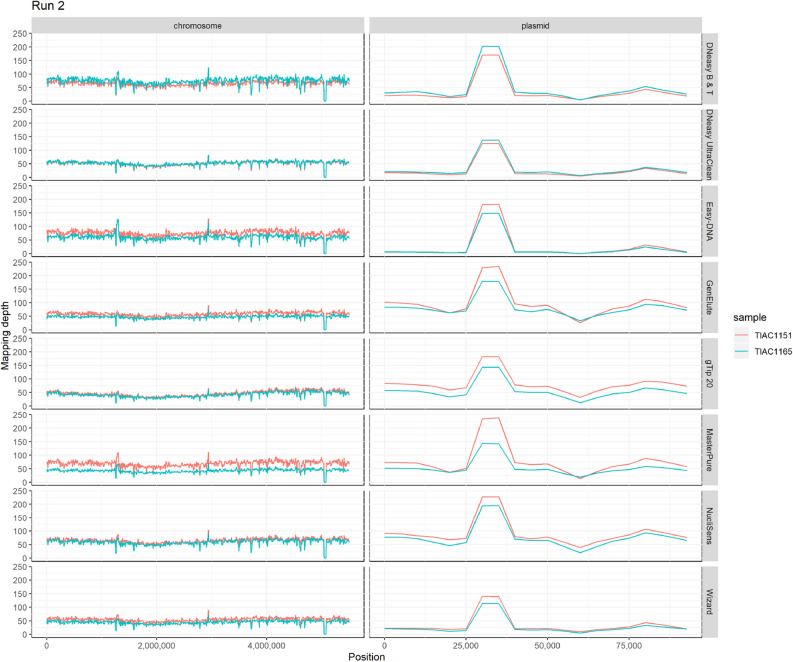
Overview of median mapping depths against the Sakai *E. coli* O157:H7 reference genome and the Sakai *E. coli* pO157 plasmid for sequencing run replicates TIAC1151 and TIAC1165 per kit, sequenced in run 2. The median read mapping depth for each sample was calculated using a sliding window of 10,000 bases shifted by 5,000 bases for each data point. Abbreviations: DNeasy Blood & Tissue kit (DNeasy B & T), DNeasy UltraClean Microbial kit (DNeasy UltraClean), Easy-DNA gDNA Purification kit (Easy-DNA), GenElute Bacterial gDNA kit (GenElute), Genomic-tip 20/G kit (gTip 20), MasterPure Complete DNA Purification kit (MasterPure), NucliSENS miniMag (NucliSens), Wizard gDNA Purification kit (Wizard).

Per kit for each isolate, one DNA replicate was chosen for Nextera XT library preparation and subsequent MiSeq sequencing over three different sequencing runs according to the set-up in Supplementary Table S4 online. For the sake of simplicity, the resulting data of an isolate processed with a specific kit are referred to as ‘sample’, whereas the resulting data of the seven isolates for the eight kits are referred to as ‘all samples’. The two isolates (TIAC1151 and TIAC1165) for each kit included in each sequencing run are referred to as ‘sequencing run replicates’. Quality control checks were performed on the raw data to evaluate the kit’s influence on read quality. None of the samples and sequencing run replicates exhibited any indication of potential contamination, using a cut-off of > 1% of reads being classified to any species other than *E. coli* with Kraken. Per kit across the seven samples, the median sequencing depth against the assemblies was always > 35-fold. In all three sequencing runs, no significant differences (run 1 p-value: 1.75 × 10^–1^; run 2 p-value: 2.23 × 10^–1^; run 3 p-value: 8.11 × 10^–2^) were observed between median sequencing depths of the sequencing run replicates obtained per kit (Kruskal–Wallis, n: 16, α: 0.05). Overall, evenness of read distributions over the Sakai *E. coli* O157:H7 reference genome and Sakai *E. coli* pO157 plasmid reference was independent of the employed kit (Fig. 1) and sequencing run (Supplementary Fig. S1 online). Moreover, median read mapping depths against the Sakai *E. coli* pO157 plasmid reference showed a peak in a region from 30.8 to 32.0 kb annotated as a transposase (GenBank accession: NP_052637.1), and was therefore potentially inserted multiple times in the pO157 plasmid of isolates TIAC1151 and TIAC1165 compared to the Sakai *E. coli* pO157 plasmid reference. The online Supplementary Tables give more information on read trimming statistics (Supplementary Table S5), assembly statistics (Supplementary Table S6), and advanced quality control statistics (Supplementary Table S7).

### Kit’s influence on isolate characterization

#### Serotype determination

All samples and sequencing run replicates were serotyped in silico by aligning the assembled contigs against the reference sequences in the SerotypeFinder database with BLAST + . Serotypes matching the conventional serotyping results were identified for 94.32% of all samples and sequencing run replicates (n: 88). No ambiguities for H-typing were observed across all samples and sequencing run replicates. O-typing did not succeed for five samples (i.e. 5.68%) generated with different kits on a total of 88 (all samples and sequencing run replicates), due to ambiguous typing using BLAST + detection of the O-antigen determining *wzx* and *wzy* genes. Ambiguous O-typing of sequencing run replicates was not consistent over different sequencing runs, indicating that O-typing performance was affected by the sequencing process, rather than the choice of the kit. Detection using direct read mapping with SRST2 did not resolve ambiguous O-typing in four of these five samples. Further investigation (Supplementary Notes online) indicated that ambiguous O-typing was caused by assembly artifacts affecting the detection of the corresponding *wzx* and/or *wzy* alleles. Moreover, the median sequencing depth in the region of contigs aligning to the *wzx* and/or *wzy* genes of the five ambiguous O-typed samples was only 3.99 (Interquartile range (IQR): 4.22). The median sequencing depth in the contig regions aligning to both O-typing genes in all accurately serotyped samples and sequencing run replicates (17.31, IQR: 15.74) was higher than in the ambiguously O-typed samples, but still consistently lower than the overall median sequencing depth against assemblies of all samples and sequencing run replicates (45.00, IQR: 15.00). Notably, the O-antigen determining genes *wzx* and *wzy* are located in a low %GC-content region (2,782.5 to 2,787.5 kb) of the Sakai *E. coli* O157:H7 reference genome, visualized in Supplementary Fig. S2online*.*

#### Virulence genotype profile determination

**Figure 2:**
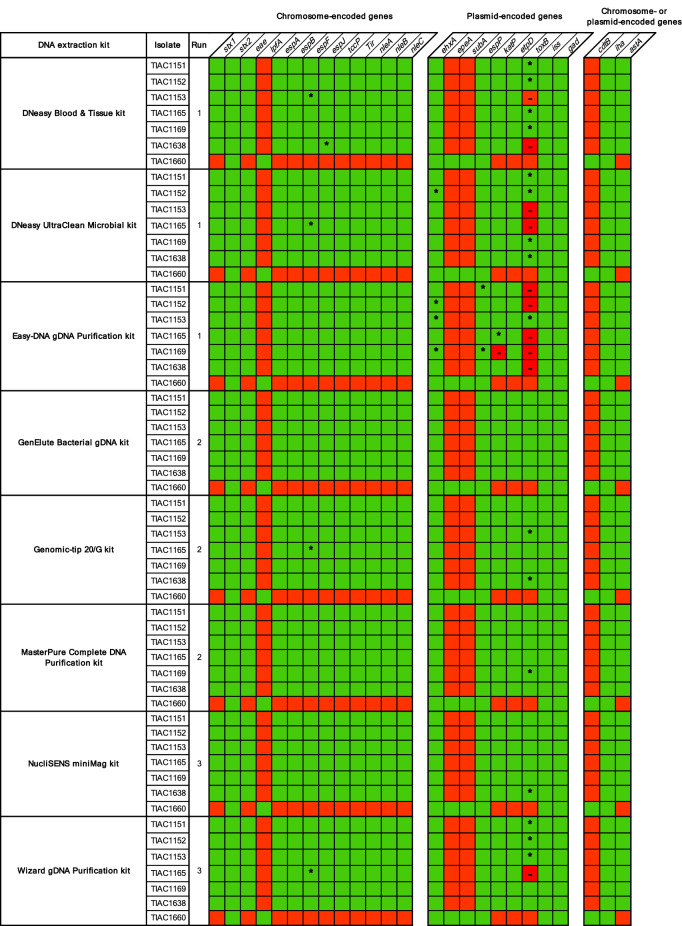
Overview of the virulence genotype obtained for all samples. Presence and absence of virulence genes are indicated in green and red, respectively, as determined using BLAST + and SRST2. *Virulence genes detected only with SRST2; —Missed virulence genes, referred to as false negatives (neither detected with SRST2 nor BLAST + while presence of the gene was expected, i.e. detected in the same isolate processed with a different kit, or detected in a sequencing run replicate of the isolate).

The virulence genotype profile was determined in silico using the VirulenceFinder database for all samples (Fig. 2) and sequencing run replicates (Supplementary Fig. S3 online). A virulence gene was expected to be present in an isolate when it could be detected using BLAST + and/or SRST2 in any of its samples. In total, the presence or absence of 25 different virulence genes, of which nine were plasmid-encoded, was determined for all samples and sequencing run replicates. Detection of *stx1/2*, *eae* and *ehxA* corresponded to the results of conventional typing methods (Table 2). The frequencies of detected and undetected genes of sequencing run replicates were compared, but no effect of the sequencing run on virulence genotyping could be identified (Chi-square test, n: 1,200, α: 0.05, p-value: 9.94 × 10^–1^). Hence, all statistical analyses evaluating the effect of the kit on virulence genotyping were subsequently performed on all samples. No significant influence of the kits on detected virulence gene profiles with at least one of the two detection methods could be observed (Chi-square Test, n: 1,400, α: 0.05, p-value: 9.95 × 10^–1^). When comparing gene profiles obtained with SRST2 and BLAST + separately for all samples and sequencing run replicates, SRST2 provided more sensitive gene detection compared to BLAST + in regions with low sequencing depth. On a total of 1,400 observations (i.e. 25 genes detected across all samples (n: 56)), 39 genes expected to be present and of which 34 were plasmid-encoded, were missed with BLAST + . For 28 genes of those 39, SRST2 could resolve the missed gene detection. These 28 genes were distributed over 25 samples, from which the DNA of the majority (i.e. 20 samples) was prepared with the Wizard gDNA Purification (4/7), DNeasy Blood & Tissue (6/7), DNeasy UltraClean Microbial (5/7), and Easy-DNA gDNA Purification (5/7) kits. The median mapping depth in these 28 genes was only 4.87 (IQR: 3.09). Sixteen out of these 28 were linked to the plasmid-encoded *toxB* gene. Additionally, over all samples, 11 genes could not be detected using both detection methods (indicated as ‘-’ in Fig. 2). These 11 false negatives were distributed over ten samples, again all generated with the four kits mentioned above. All false negatives were linked to the *toxB* gene, except for one that was linked to the plasmid-encoded *katP* gene. Because the missed detection of *toxB* was not consistent over sequencing run replicates (Supplementary Fig. S3 online) and the applied computational methods allow variation compared to the reference gene sequences (maximum 10% nucleotide divergence and minimum 60% query coverage), this is unlikely to be caused by a new variant that was absent in the VirulenceFinder database. Figure 1 indicates a systematic decrease in median sequencing depth of the pO157 plasmid in sequencing run replicates TIAC1151 and TIAC1165, over all kits and irrespective of the sequencing run (Supplementary Fig. S1 online), in the region from 55.5 to 65.5 kb containing the *toxB* gene. Notably, a decrease in %GC-content to ~ 30% in this region was found compared to the average %GC-content of 47.71% of the Sakai *E. coli* pO157 plasmid reference (Supplementary Fig. S4 online).

#### Detection of genetic antimicrobial resistance and concordance with phenotypic susceptibility testing

The genotypic AMR profile was determined by using BLAST + and SRST2 with the ResFinder database for all samples and sequencing run replicates (Table 2). The resulting genotypic AMR profile per sample was independent from the used kit, since the genotypic profile for each respective sample generated with one kit was identical to the retrieved genotypic profile of that sample generated with all other kits. From the phenotypical AMR tests that were performed on the five isolates (TIAC1151, TIAC1152, TIAC1153, TIAC1165 and TIAC1169), only TIAC1153 was resistant to ampicillin, kanamycin, streptomycin, sulphonamides, tetracyclin and trimethoprim, which was consistent with prediction results of WGS AMR genotyping.

### Kit’s influence on WGS of plasmid DNA

**Figure 3:**
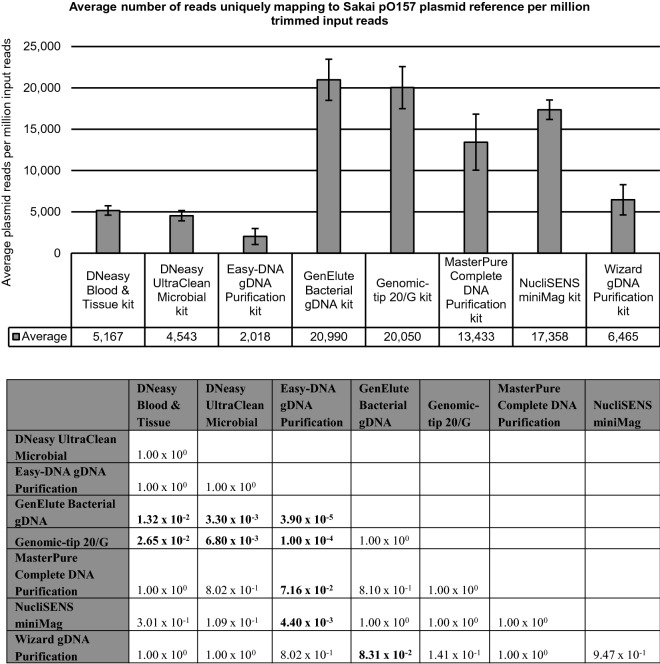
Average number of reads mapping uniquely to the Sakai *E. coli* pO157 plasmid reference normalized per one million trimmed input reads for the eight kits. Number of reads mapping uniquely against the Sakai *E. coli* pO157 plasmid reference per million input reads when mapping simultaneously against the Sakai *E. coli* pO157 plasmid (NC_002128.1) and Sakai *E. coli* O157:H7 genome (NC_002695.2) reference. Values are averaged over all *E. coli* O157:H7 samples (TIAC1151, TIAC1152, TIAC1153, TIAC1165, TIAC1169 and TIAC1638) that were generated with each kit, without inclusion of the sequencing run replicate results for TIAC1151 and TIAC1165. Bars represent the standard deviation across samples for each kit. Significant differences in average plasmid reads per million trimmed input reads were identified with the Kruskal–Wallis test (n: 48, α: 0.05, p-value: 2.80 × 10^–7^) followed by Dunn post-hoc analysis with Holm correction, as depicted in the accompanying table with significant values depicted in bold.

The influence of the kit on WGS of plasmid DNA was analyzed for the pO157 plasmid in *E. coli* O157:H7 samples. Median read mapping depth against the Sakai *E. coli* pO157 plasmid reference was observed to be consistently lower relative to the Sakai *E. coli* O157:H7 reference genome for the samples generated with the Easy-DNA gDNA Purification, DNeasy Blood & Tissue, DNeasy UltraClean Microbial, and Wizard gDNA Purification kits compared to the other kits (Fig. 1). Moreover, unique read mapping against both references was performed for all *E. coli* O157:H7 samples (i.e. TIAC1151, TIAC1152, TIAC1153, TIAC1165, TIAC1169, and TIAC1638). The average number of reads mapping uniquely against the pO157 plasmid reference per million input reads over the six *E. coli* O157:H7 samples was compared per kit (Fig. 3). Based on this number, a significantly lower efficiency of plasmid extraction was identified for the Easy-DNA gDNA Purification, DNeasy UltraClean Microbial, DNeasy Blood & Tissue and to a lesser extent the Wizard gDNA Purification kits, compared to the GenElute Bacterial gDNA and Genomic-tip 20/G kits that had the highest efficiency (Kruskal–Wallis test, n: 48, α: 0.05, p-value: 2.80 × 10^–7^, Dunn post-hoc analysis with Holm correction, see Fig. 3). It was verified that all kits fully extracted the pO157 plasmid, since 100% of the Sakai *E. coli* pO157 plasmid reference was covered for sequencing run replicates TIAC1151 and TIAC1165, irrespective of the employed kit (Fig. 1 and Supplementary Fig. S1 online).

### Kit’s influence on inferring isolate relationships

The influence of kits on inferred phylogenetic relationships between samples was determined through comparison of both core genome multilocus sequence typing (cgMLST)- and single nucleotide polymorphism (SNP)-based typing with the a priori known relationship.

#### cgMLST-based typing

**Figure 4:**
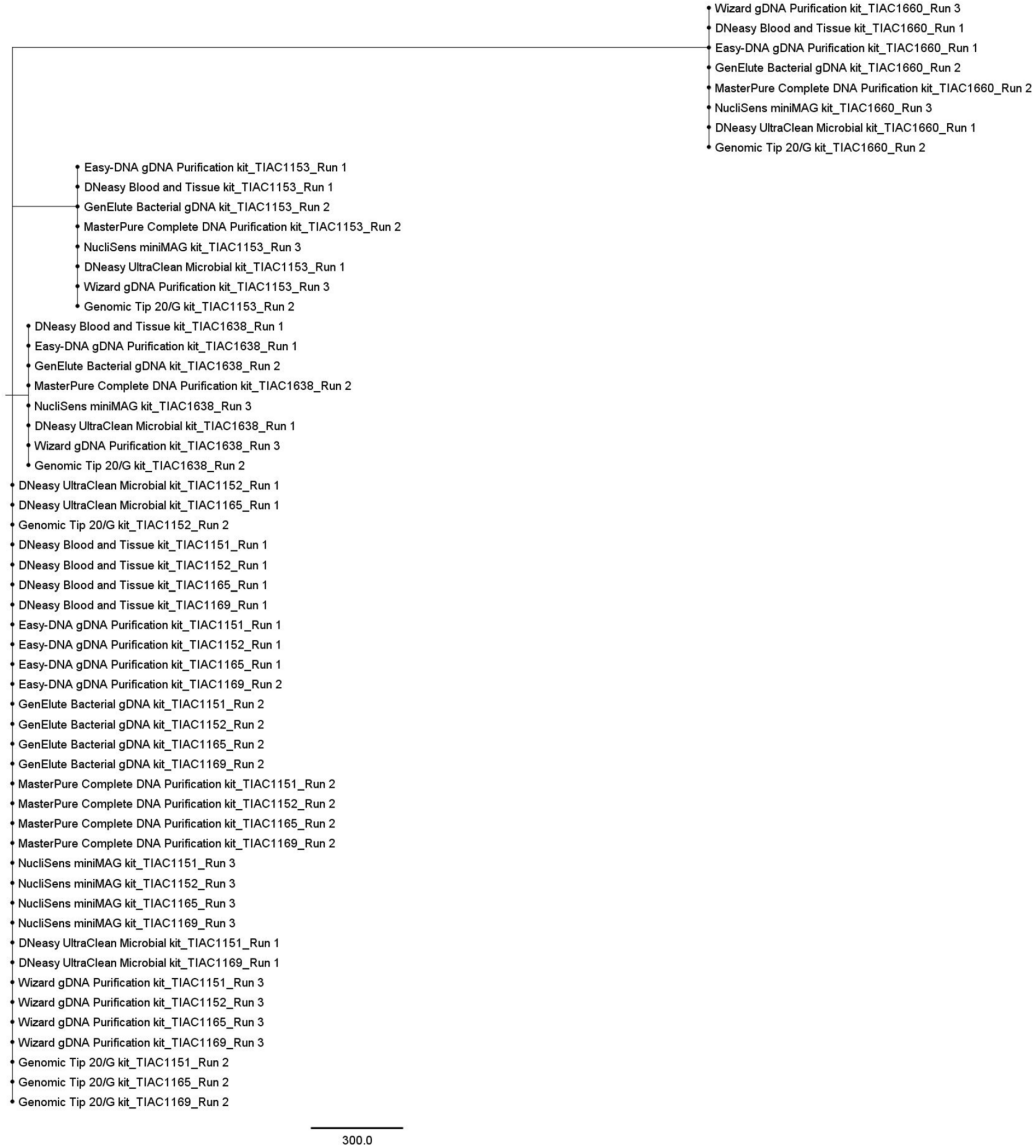
cgMLST-based tree of all samples. A minimum spanning tree was created with GrapeTree using the MSTreeV2 method on all outbreak and non-outbreak samples generated with the eight kits, excluding sequencing run replicates. All outbreak samples (TIAC1151, TIAC1152, TIAC1165 and TIAC1169) consistently cluster together, while non-outbreak samples TIAC1153, TIAC1638 and TIAC1660 are separated from the outbreak cluster and delineated per isolate. The scale bar represents the number of cgMLST allele differences between samples. One cgMLST allele difference with other outbreak samples was observed for only four samples (TIAC1152 generated with the Genomic-tip 20/G, TIAC1152 generated with the DNeasy UltraClean Microbial kit, TIAC1165 generated with the DNeasy UltraClean Microbial kit, and TIAC1153 generated with the Easy-DNA gDNA Purification kit), which is not visible in the figure, because of the large scale.

On average, 99.32% ± 0.90% (s.d.) of all 2,513 core genome loci could be detected with 100% query coverage and sequence identity, irrespective of the sequencing run and kit. In Fig. 4, the relatedness of all samples based on cgMLST is shown. A visual representation of the relationship between all samples and sequencing run replicates is also shown in the online Supplementary Fig. S5. Both cgMLST trees demonstrate that neither the used kit (demonstrated by Fig. 4 and Supplementary Fig. S5) nor the sequencing run (demonstrated by Supplementary Fig. S5) influenced retrieved relationships between isolates, since all outbreak samples (in Fig. 4 and Supplementary Fig. S5) and their sequencing run replicates (in Supplementary Fig. S5) consistently clustered together in one large single clade carried by one single branch, separated from the non-outbreak samples that clustered together per isolate. Zero cgMLST allele differences existed within the outbreak clade, except for three samples in which one cgMLST allele difference was found (Fig. 4). In one non-outbreak sample, also one cgMLST allele difference was observed compared to the cgMLST profile of the other samples of the same isolate (Fig. 4). These differences could be explained by an allele that was not called as a perfect hit, due to fragmentation of the assembly (Supplementary Notes online).

#### SNP-based typing

**Figure 5:**
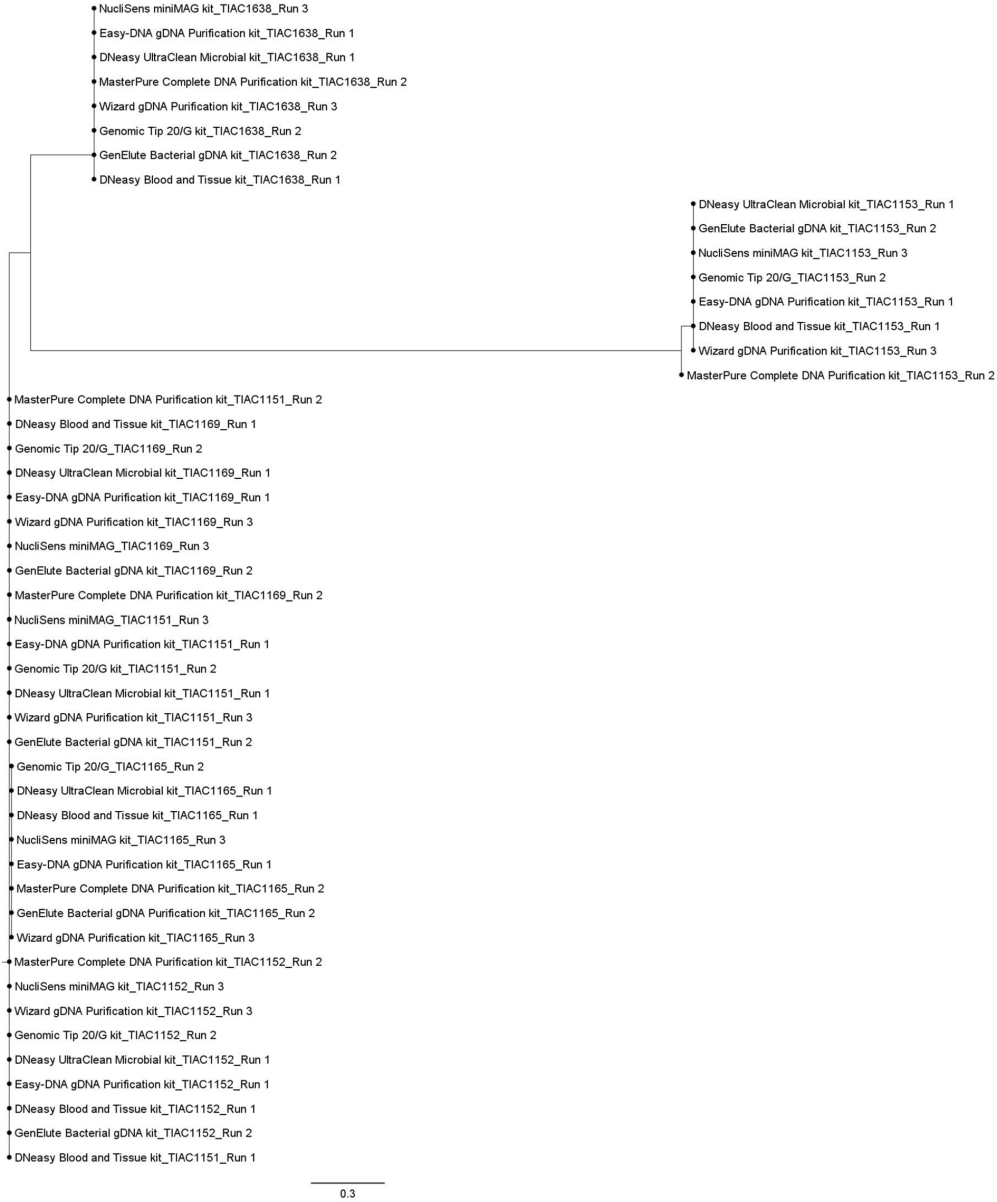
SNP-based tree of all O157:H7 samples. A maximum likelihood SNP tree was generated using the K2 nucleotide substitution model, containing all O157:H7 samples. Non-O157:H7 samples (TIAC1660) were excluded from SNP calling, due to high divergence from the Sakai *E. coli* O157:H7 reference genome. All outbreak samples (TIAC1151, TIAC1152, TIAC1165 and TIAC1169) consistently clustered together, irrespective of the employed kit. Within the outbreak clade, for all TIAC1165 samples, a limited number of discrepant SNPs with other outbreak samples existed, largely confined to a hypothetical transposase region (*ydcC* gene). The non-outbreak samples (TIAC1153 and TIAC1638) were separated from the outbreak clade, and clustered together per isolate. Notably, for TIAC1153 samples, a small number of SNPs different with the reference genome between the sample generated with the MasterPure Complete DNA Purification kit and all other TIAC1153 samples, was observed. This difference was solely due to masking of a low-quality region (see “Results”). The distance scale bar represents the average number of nucleotide substitutions per site.

A phylogenetic tree was created based on overall SNPs called against the Sakai *E. coli* O157:H7 reference genome between all *E. coli* O157 samples (Fig. 5). A visual representation of the relationships between all samples and sequencing run replicates based on SNPs is represented in the online Supplementary Fig. S6*.* A similar phylogeny compared to the one observed in the cgMLST tree was obtained. The relationships between the isolates were not affected by the sequencing run and kit, since no discrepant SNPs (i.e. a SNP found in one of the samples of an isolate compared to the Sakai *E. coli* O157:H7 reference genome that is however not consistently observed in all samples of the same isolate) were identified in pairwise comparisons of the SNP profiles in shared high-quality positions of each sequencing run replicate over the three runs (i.e. each sequencing run replicate always had exactly the same SNPs compared to the Sakai *E. coli* O157:H7 reference, irrespective of the sequencing run; Supplementary Fig. S6 online), and since no discrepant SNPs were identified in pairwise comparisons of the SNP profiles in shared high-quality positions of each included sample generated with a respective kit (i.e. each sample of the same isolate always had exactly the same SNPs compared to the Sakai *E. coli* O157:H7 reference, irrespective of the used kit). However, as visible in Fig. 5, sample TIAC1153 generated with the MasterPure Complete DNA Purification kit was located on a different branch, although at very close distance, compared to all other TIAC1153 samples. Moreover, the median number of discrepant SNPs compared to the Sakai *E. coli* O157:H7 reference genome between outbreak samples per kit differed between the eight kits, ranging from two to eight (SNP matrices in Supplementary Table S8 online). However, no significant influence of the kit on the obtained number of discrepant SNPs compared to the reference genome between outbreak samples could be identified (Kruskal–Wallis, n: 48, α: 0.05, p-value: 6.16 × 10^–1^). Most of these discrepant SNPs between the outbreak samples were specifically located in the TIAC1165 samples. Notably, as shown in Supplementary Table S9 online*,* the large majority of discrepant SNPs that were called in sample TIAC1165 were located in the chromosomal region from 274,300 to 274,400 bp, irrespective of the employed kit. This chromosomal region is annotated as the hypothetical transposase encoding gene *ydcC* (GenBank NP_308268.1), suggesting that these are bona fide SNPs present only in the TIAC1165 samples.

### Influence of EDTA on isolate characterization and inferring isolate relationships

The influence of EDTA in the DNA solution buffers of four kits, and removing EDTA using the gDNA Clean & Concentrator kit from DNA extracts obtained with the DNeasy Blood & Tissue kit (Supplementary Table S1 online), was evaluated at different levels of the WGS workflow for TIAC1151, TIAC1165 and TIAC1660 (Supplementary Note, accompanied by Supplementary Fig. S3, S5, S6, S7, S8, and Supplementary Tables S10, S11 online). Overall, no effect of EDTA on WGS data quality, isolate characterization and isolate relationship retrieval could be determined.

## Discussion

In our study, several parameters have been quantified with the aim to evaluate the relative effectiveness of eight different DNA extraction kits based on their WGS output for surveillance and outbreak investigation of a foodborne pathogen.

First, DNA quality control (QC) results demonstrated that all kits rendered sufficient amounts of starting material for Nextera XT library preparation^33^. Although not all kits met Illumina’s DNA quality recommendations^33^ (Table 1), no influence of DNA purity on read quality after sequencing was observed from the advanced QC checks. The choice of the kit did therefore not influence sequencing quality.

Next, isolate characterization was limitedly impacted by the kit’s plasmid extraction performance. The majority of the 39 discrepant observations for assembly-based virulence genotyping (indicated as ‘*’ and ‘-’ in Fig. 2) were plasmid-encoded, and could mostly be explained by low sequencing depths in the corresponding regions^34^. Moreover, these issues were more common in samples generated with the Easy-DNA gDNA Purification, DNeasy UltraClean Microbial, DNeasy Blood & Tissue, and Wizard gDNA Purification kits. As deduced from the number of reads mapping to the Sakai *E. coli* pO157 plasmid reference per million trimmed input reads, these kits were indeed significantly less efficient in extracting plasmid DNA. Salting-out kits were previously found to exhibit more difficulties with extracting small plasmids (< 5 kb) from *Klebsiella pneumoniae*^16^. However, in addition to the STEC pO157 plasmid being 92 kb in size^19^, we observed impaired plasmid DNA extraction for both solid-phase and salting-out extraction methods (Table 1). Therefore, we could not confirm this association for STEC.

Nevertheless, failed assembly-based virulence gene detection could be resolved with SRST2 (direct read mapping) in 28 of 39 observations. The remaining 11 observations were mainly linked to the plasmid-encoded *toxB* gene, and only occurred in samples generated with kits that had lower plasmid DNA extraction efficiency. Notably, *toxB* exhibits a %GC-content decrease, similar to the O-typing genes *wzx* and *wzy*. Although WGS-determined serotyping matched with traditional methods in the majority of samples and sequencing run replicates, ambiguous serotyping (5.68%) was only caused by O-typing issues. However, since this was not consistent over sequencing run replicates, no influence of the kit on O-typing was suggested. Likely, these sequencing depth drops were the indirect result of their lower %GC-contents coupled with using Nextera XT^35,36^. However, despite the sensitivity of the Nextera XT library preparation to %GC-bias, its protocol convenience, and possibility to run under an ISO/IEC17025 accredited environment^37^, render the library preparation kit still attractive for routine use. With regard to AMR genotyping, we did not identify any influence of the kit, in agreement with Pasquali et al*.*, 2019^15^. However, considering the few AMR genes detected, a larger study evaluating the kit influence on AMR genotyping is of interest.

Besides characterization, the influence of the kit on isolate relationships was evaluated. With both cgMLST- and SNP-based phylogenetic inferences, all outbreak samples clustered together and were clearly separated from non-outbreak samples, regardless of the applied kit. Regarding cgMLST, in four outbreak and non-outbreak samples generated with three different kits, one cgMLST allele difference was identified, however, without affecting the predefined relatedness between the samples. This is likely because on average 99.32% of cgMLST loci could be detected so that enough information was present in the allele call matrix to reliably resolve phylogenetic relationships. Regarding SNPs, our results were consistent with Pasquali et al., 2019^15^, since no SNP discrepancies were identified in pairwise comparisons between different kits per isolate, i.e. every sample had exactly the same SNPs in shared high-quality positions compared to the Sakai *E. coli* O157:H7 reference genome when generated with the different kits. Kit choice did therefore not affect obtained SNP numbers when considering individual isolates. It should however be noted that ignored/masked positions (with lower quality) can be different for determining pairwise SNP differences than for constructing SNP matrices. This explains why some samples with zero SNP distance were positioned on different but closely located branches (Fig. 5). Only limited SNP discrepancies compared to the Sakai *E. coli* O157:H7 reference genome existed amongst the outbreak samples when generated with the different kits, which were the result of lower quality positions (mapping quality < 30) that had been masked/ignored. Interestingly, nearly all identified SNP discrepancies were, irrespective of kit, situated in isolate TIAC1165, within the *ydcC* gene encoding a hypothetical transposase^38^, suggesting these constitute ‘real’ SNP differences. The biological significance of these SNPs is currently unknown.

Overall, the presence of EDTA in DNA extracts used for WGS is not recommended^33^. Nevertheless, in our study we found that data quality, isolate characterization, and relatedness were not affected by EDTA presence (< 1.0 mM). With regard to its function in DNA preservation^39^, EDTA presence can therefore even be regarded as beneficial.

When putting these results in the perspective of outbreak scenarios, kit choice amongst those tested, including those containing EDTA, has only limited influence on cgMLST- and SNP-determined isolate relationships, which is beneficial for WGS data sharing. However, the few identified discrepancies between kits indicate that applying rigid cgMLST allele and/or SNP thresholds for outbreak cluster definition can potentially be dangerous. Particularly important for surveillance and less for outbreak investigation is that the kit’s plasmid extraction performance slightly influences the completeness of STEC characterization. Moreover, although we confirm that > 35-fold sequencing depth is sufficient for cost-efficient STEC characterization^14^, irrespective of the kit, we demonstrated that care must be taken for detection of low %GC-content genes in combination with Nextera XT library preparation, especially when a kit with impaired plasmid extraction is applied. In this perspective, our results suggest to apply SRST2-based STEC characterization, and to select a kit with efficient extraction performances of both chromosomal and plasmid DNA. Pooling less samples in the same run, as often the case in routine sequencing^40^, can prevent these detection issues, however, at the expense of a higher cost per sample. Additionally, kit selection for laboratory uptake can be based on other relevant criteria (Table 1), such as cost and handling convenience of workflows. Salting-out workflows are less expensive than solid-phase protocols, but were evaluated as less convenient. In a routine setting, kits with the possibility to automate (e.g. NucliSENS miniMag) are preferred. Moreover, the universality of a kit, i.e. appropriateness for different species and/or sequencing platforms (e.g. Oxford Nanopore Technologies—ONT) can be an important factor for kit selection in surveillance laboratories. Indeed, ONT generates long reads in real-time that are helpful for assembly issues and plasmid reconstruction. However, it requires high-quality DNA^41^, has a higher sequencing error rate and is less suitable in routine settings due to its rapid evolution. The GenElute Bacterial gDNA kit meets all criteria mentioned above, i.e. high-quality DNA extraction, good representation of chromosomal and plasmid DNA (for STEC), relatively cost-effective, user-friendly and includes protocols for Gram-positive bacteria, rendering it attractive to evaluate its suitability for other sequencing platforms and pathogens.

In conclusion, this comparative study was set up to extensively evaluate the performance of DNA extraction kits for Nextera XT library preparation and MiSeq WGS, focusing on isolate characterization and discrimination. Our conclusions are extendable, especially since the evaluated kits are also frequently used for other foodborne pathogens. Our study contributes to standardization supporting global WGS data portability, which is of particular relevance for controlling foodborne and other pathogens of clinical and public health interest.

## Methods

### STEC isolate selection

The selection of isolates for this study was based on assessed characteristics (Table 2) with previous, conventional analysis, and included: (i) four epidemiologically linked and laboratory confirmed outbreak isolates; and (ii) three non-outbreak isolates, from which one was collected during the outbreak (TIAC1153), and two outside the outbreak period (TIAC1638 and TIAC1660). Two of these non-outbreak isolates had the same serotype as the outbreak isolates, i.e. O157:H7, containing the pO157 plasmid^19^. The third non-outbreak isolate was identified as O113:H21, containing the pO113 plasmid^20^, which enabled to investigate plasmid extraction performances of the kits. All isolates were provided by the Belgian National Reference Laboratory and Center for STEC (NRL-STEC and NRC-STEC, respectively).

### Bacterial growth conditions

Isolates were preserved in a glycerol-Brain Heart Infusion (BHI) broth stock (40%) at minus 80 °C until analysis. A loopful of each glycerol stock was streaked onto separate Nutrient Agar (NA) plates and incubated overnight (16 h) at 37 °C. A single colony of each isolate was inoculated and sub-cultured in 10 ml of BHI, and incubated at 37 °C overnight. The number of cells was estimated by measuring Optical Density at 600 nm (OD600) in the linear range using the SmartSpec Plus spectrophotometer (Bio-Rad Laboratories Inc, Hercules, CA).

### DNA extraction

Total DNA (i.e. chromosomal and plasmid) from the bacterial cultures and a blank (BHI incubated overnight (16 h) at 37 °C) were extracted in biological triplicates using eight kits (Table 1) that were selected through literature review, incorporating differently existing methods and taking into account: (i) recommendations from leading institutes and companies in the field; (ii) automation possibilities; (iii) extraction completion time; and (iv) cost per sample. The kits were used according to the manufacturer’s recommendations, except for the DNeasy Blood & Tissue (Qiagen, Hilden, Germany), GenElute Bacterial Genomic DNA (gDNA) (Sigma-Aldrich, Missouri, US), Wizard gDNA Purification (Promega, Wisconsin, US) and MasterPure Complete DNA Purification (Lucigen Corporations, Wisconsin, US) kits, for which DNA was rehydrated or eluted in 10 mM Tris–HCl (pH 8.5) due to the presence of EDTA in the supplied DNA solution buffer.

The seven isolates were additionally processed in triplicate with these four EDTA-containing kits, according to the manufacturer’s protocol, with DNA rehydration/elution in the supplied buffer (Supplementary Table S1 online). It was investigated whether EDTA removal from an already existing stored DNA extract would impact downstream WGS results. Hereto, the seven extracts from the DNeasy Blood & Tissue kit with DNA eluted in the supplied EDTA-containing buffer were used for treatment with the gDNA Clean & Concentrator kit (BaseClear B.V., Leiden, The Netherlands) to eliminate EDTA according to the manufacturer’s instructions, while replacing the supplied Tris–EDTA (TE) elution buffer by 10 mM Tris–HCl (pH 8.5).

### Extracted total DNA quality control check

After DNA extraction, DNA concentration, purity and integrity were investigated with, respectively, the dsDNA HS and BR assay kits for the Qubit 4 fluorometer (Invitrogen, Carlsbad, CA), NanoDrop 2000 spectrophotometer (Thermo Fisher Scientific, Schwerte, Germany), and the Genomic DNA ScreenTape and Reagent kits for TapeStation 4200 electrophoresis (Agilent Technologies, Santa Clara, CA), according to the manufacturer’s recommendations. DNA extracts with A260/280 and A260/230 ratios of ~ 1.80 and 2.00 to 2.20, respectively, were considered as high-purity DNA extracts. High-purity extracts in combination with DNA integrity numbers (DIN) > 8.00 and fragment lengths > 60 kb were considered as high-quality DNA extracts. Conventional PCRs (primer sequences and concentrations, PCR program in Supplementary Table S2 online) were performed to investigate the plasmid extraction performance of each kit by targeting the plasmid-encoded *saa*^42^ (plasmid pO113) and *ehxA*^43^ (plasmid pO157 and pO113) genes, and the *E. coli* chromosomally-encoded housekeeping gene *uidA*^44^ in the triplicate extracts of TIAC1165, TIAC1660, and the blank processed with each kit. A boiled extract of both isolates and a blank containing sterile DNase/RNase-free H_2_O were considered as positive (PC) and negative (NC) PCR controls, respectively, and were prepared as described previously^45^. The PCR products were visualized with the TapeStation 4200 instrument using the D1000 ScreenTape and Reagent kits as indicated by the manufacturer’s protocol.

### Library preparation and MiSeq sequencing

Per kit for each isolate, one of the three replicates was selected for subsequent library preparation and WGS. For EDTA effect evaluation, one of three isolates for each of the four EDTA-containing kits, including two outbreak isolates (TIAC1151 and 1165) and one non-outbreak isolate (TIAC1660), were selected for subsequent library preparation and WGS to allow joint sequencing in the same run. One nanogram (in 5 µl) of each selected DNA extract was used as template to construct Nextera XT sequencing libraries (Illumina, San Diego, CA) according to the manufacturer’s recommendations. Library fragmentation length and library DNA concentration were evaluated using the TapeStation 4200 with the HS D5000 ScreenTape and Reagent kits and dsDNA HS assay kit for the Qubit 4 fluorometer, respectively. All libraries were sequenced on a MiSeq instrument (Illumina, San Diego, CA) distributed over four different runs (Supplementary Table S4 online) using the MiSeq V3 chemistry, as described by the manufacturer’s protocol, for the production of 2 × 250 bp paired-end reads.

### Data analysis

The results in this study were produced through the separate use of the individual tools. Trimmomatic 0.36^46^ was used for trimming raw reads by removing Nextera XT adaptors and other Illumina-specific sequences (‘Illuminaclip’ set to value ‘NexteraPE-PE.fa:2:30:10’), removing low-quality residues at read beginnings and endings (‘leading:10’ and ‘trailing:10’), clipping reads when average Q-scores dropped below 20 over a sliding window of four residues (‘slidingwindow:4:20’), and dropping reads shorter than 40 bases after processing (‘minlen:40’). Trimmed reads were de novo assembled using SPAdes 3.10.0^47^ setting the ‘*–careful*’ and ‘*–cov-cutoff auto*’ options to reduce mismatches and short indels, and remove low coverage contigs, respectively. Orphaned reads resulting from trimming (i.e., reads where only one read of the pair survived) were provided to the assembler as single-end reads. Relevant assembly statistics were calculated with Quast 4.4^48^. Afterwards, trimmed paired-end and orphaned reads were mapped against the de novo assembly, the Sakai *E. coli* O157:H7 reference genome (NCBI accession NC_002695.2^49–52^) and the Sakai *E. coli* pO157 plasmid reference (NCBI accession NC_002128.1^49^), using Bowtie2 2.3.0^53,54^ with the *‘–sensitive’* and *‘–end-to–end’* settings. Median read mapping depths for all alignments were determined using SAMtools depth 1.9^55^ with the ‘-*a*’ option enabled. Median read mapping depths against the Sakai *E. coli* O157:H7 reference genome and Sakai *E. coli* pO157 plasmid for sequencing run replicates TIAC1151 and TIAC1165 were visualized using ggplot2^56^ in R 3.6.1 by averaging depth values over a sliding window of 10,000 bases shifted by 5,000 bases for each data point. Additionally, to compare chromosomal versus plasmid DNA read fractions between different kits, read numbers mapping uniquely to either the plasmid or chromosome were determined by simultaneously mapping trimmed reads against the Sakai *E. coli* O157:H7 reference genome and Sakai *E. coli* pO157 plasmid reference using Bowtie2 with the ‘*-k’* parameter set to one. Reads numbers mapping uniquely to the plasmid or chromosome were then determined using SAMtools idxstats 1.9^55^, and normalized per million of (trimmed) input reads.

A contamination check was executed using Kraken 0.10.5^57^ with default parameters and a database containing all NCBI RefSeq Genome entries (database accessed January 24th, 2018) with accession prefixes NC, NW, AC, NG, NT, NS, and NZ of the following taxonomic groups: archaea, bacteria, fungi, human, protozoa, and viruses.

All samples and sequencing run replicates were genotypically characterized for the presence of AMR genes, virulence genes and serotype determining genes using two different approaches: (i) by aligning assemblies against databases with reference sequences using BLAST + 2.6.0^58,59^; and (ii) by read mapping with SRST2 0.2.0^60^ with the options *‘–max-divergence 10’*, ‘*–gene-max-mismatch 10’* and *‘–max-unaligned-overlap 100*’. Database sequences were clustered beforehand with an 80% sequence identity cut-off using the ‘cd-hit-est’ function from CD-HIT 4.6.8^61^. With SRST2, hits with < 60% query coverage and/or > 10% sequence divergence were omitted. Hits identified with BLAST + were removed when < 60% query coverage and/or < 90% sequence identity was observed. When using BLAST + , the best hit for each detected database cluster was then determined based on the method for allele scoring described by Larsen et al., 2012^62^. The ResFinder^31,32^, VirulenceFinder^32,63^, and SerotypeFinder^64^ databases were used to detect AMR genes, virulence genes, and serotype determining genes, respectively. All database sequences were retrieved from their respective sources on September 3rd 2018. In case of unexpected results, contig alignment against the corresponding reference gene using BLAST + was performed to identify assembly artifacts.

In silico cgMLST was performed as described for gene detection using the EnteroBase^65^ cgMLST scheme containing 2,513 loci (downloaded on September 3rd, 2018) to evaluate whether a priori known relationships between isolates could be retrieved. Only exact allele calls (i.e., requiring a full-length 100% identical match) were accepted. For tree construction, loci called in < 80% of samples were stripped from the allele call matrix. Minimum spanning trees based on the allele call matrix were created using GrapeTree 2.0^66^ with the ‘method’ option set to ‘MSTreeV2′, and afterwards visualized using FigTree 1.4.3^67^.

Lastly, a SNP analysis was similarly performed to evaluate retrieved relationships between isolates. All samples belonging to serotype O157:H7 were introduced to a local SnapperDB 1.0.6^68^ instance using the Sakai *E. coli* O157:H7 reference genome (NCBI accession NC_002695.2^49–52^). SNP calling was done using PHEnix 1.4.1^69^ with dependency versions BWA 0.7.17^70^, GATK 3.7^71^, SAMtools 1.9 and Picard 2.8.3^72^. The average depth cutoff was set to 20 (other options were left at default values). SNP matrices were extracted from the database using the SnapperDB ‘get_the_snps’ function with the ‘Soft Core’ alignment type, which outputs alignments where at least 80% of the samples are A/C/T/G at each position, low-quality positions are masked as ‘N’ in the alignment. The most appropriate nucleotide substitution model for the SNP matrices was selected and used to construct maximum likelihood phylogenies with MEGA 7.0.20^73^ using all sites, a Subtree-Pruning-Regrafting (SPR) level of 5, a very weak branch swap filter and 100 bootstrap replicates, after which phylogenetic trees were visualized with FigTree.

### Statistical analysis

Obtained datasets (sequencing depth against de novo assemblies, sequencing depths in specific contig regions, mapping depths against certain genes, and detected SNP numbers) were descriptively summarized using the median and interquartile range (IQR). Descriptive analyses of percentages (DNA yield, DNA concentration, DNA purity, DIN, %cgMLST loci detected and %GC-content) were performed using the average and standard deviation (s.d.). Statistical analyses were performed using the R programming language 3.6.1^74^. The null hypothesis was rejected when obtained p-values were < 0.05. The Kruskal–Wallis test is a non-parametric test used to analyze medians of three or more groups with limited sample sizes to: (i) analyze the DNA purity (based on A260/230) across the triplicate DNA extracts per kit; (ii) evaluate the median sequencing depths of the sequencing run replicates obtained per kit against the assemblies; (iii) evaluate read numbers of all samples per kit uniquely mapping to the plasmid reference per million of input trimmed reads; and (iv) analyze median SNP discrepancies between outbreak isolates for the eight kits. Mutual comparison of test groups was performed using a Dunn post-hoc analysis with Holm correction. The Chi-squared test is a non-parametric test that was used to investigate whether: (i) the sequencing run affects observed frequencies of detected virulence genes for the sequencing run replicates; and (ii) the choice of kit impacts observed frequencies of detected virulence genes in all samples. Other statistical analyses performed to investigate the influence of EDTA present in DNA solution buffers of four kits are stated in the Supplementary Methods.

## Supplementary information

Supplementary Information.

## Data Availability

The data generated and analyzed during the current study are available in the NCBI SRA repository (https://www.ncbi.nlm.nih.gov/sra) under accession number PRJNA574887 (in-house sequenced data) and its accession numbers are listed in Supplementary Table S11 online.
