# Supplementary material for: Impact of DNA extraction on whole genome sequencing analysis for characterization and relatedness of Shiga toxin-producing Escherichia coli isolates
Source: Sci Rep. 2020 Sep 4;10:14649. doi: 10.1038/s41598-020-71207-3 (PMC7474065; doi:10.1038/s41598-020-71207-3)
Supplement: Supplementary file 1 — Supplementary Information. [file 41598_2020_71207_MOESM1_ESM.pdf]

Impact of DNA extraction on Whole Genome Sequencing analysis for characterization and relatedness of Shiga toxin-producing *Escherichia coli* isolates

Stéphanie Nouws[1,2,$], Bert Bogaerts[1,2,$], Bavo Verhaegen[3], Sarah Denayer[3], Denis Piérard[4] , Kathleen Marchal[2,5,6], Nancy H. Roosens[1], Kevin Vanneste[1,$], Sigrid C. J. De Keersmaecker[1,$,*]

[1] *Transversal activities in Applied Genomics, Sciensano, Brussels, Belgium*

[2] *Department of Information Technology, IDLab, Ghent University, IMEC,Ghent, Belgium*

[3] *National Reference Laboratory for Shiga toxin-producing Escherichia coli (NRL-STEC), Foodborne Pathogens, Sciensano, Brussels, Belgium*

[4] *Vrije Universiteit Brussel (VUB), Universitair Ziekenhuis Brussel (UZ Brussel), Department of Microbiology and Infection Control, National Reference Center for Shiga toxin-producing Escherichia coli (NRC-STEC), Brussels, Belgium*

[5] *Department of Plant Biotechnology and Bioinformatics, Ghent University, Ghent, Belgium*

[6] *Department of Genetics, University of Pretoria, Pretoria, South Africa*

* *Corresponding author* (sigrid.dekeersmaecker@sciensano.be)

[$]*Equally contributed*

Supplementary Information

CONTENTS

1. Supplementary Methods **3**
1.1. Statistical analyses of WGS data from EDTA containing samples 3
2. Supplementary Notes **3**
2.1. Investigation on the ambiguous serotyping 3
2.2. Investigation on the ambiguous detection of cgMLST alleles 4
2.3. The influence of EDTA on WGS data quality, isolate characterization and relatedness between the isolates 4
3. Supplementary Tables **7**
3.1. Supplementary Table S1: Summary of selected DNA extraction kits for evaluation of EDTA influences 7
3.2. Supplementary table S2: PCR conditions and primer sequences for detection of plasmid-specific genes 7
3.3. Supplementary Table S3: Conventional PCR results 8
3.4. Supplementary Table S4: Set-up of the sequencing runs 9
3.5. Supplementary Table S5: Read trimming statistics 10
3.6. Supplementary Table S6: Assembly statistics 17
3.7. Supplementary Table S7: Advanced quality control statistics 24
3.8. Supplementary Table S8: SNP matrix per kit containing outbreak isolates 31
3.9. Supplementary Table S9: Location of discrepant SNPs 33
3.10. Supplementary Table S10: Influence of EDTA on SNP number between outbreak isolates per kit 35
3.11. Supplementary Table S11: Accession numbers of datasets in NCBI SRA 36
4. Supplementary Figures **41**
4.1. Supplementary Fig. S1: Median mapping depths against Sakai *E. coli* O157:H7 reference genome and Sakai *E. coli* pO157 plasmid 41
4.2. Supplementary Fig. S2: %GC-content decrease in O-typing region of the Sakai *E. coli* O157:H7 reference genome 42
4.3. Supplementary Fig. S3: Overview of virulence genotype obtained per sequencing run replicate and EDTA-containing samples 43
4.4. Supplementary Fig. S4: %GC-content decrease in *toxB*-region of the Sakai *E. coli* O157:H7 plasmid reference genome 44
4.5. Supplementary Fig. S5: cgMLST tree containing all samples, sequencing run replicates and EDTA-containing samples 44
4.6. Supplementary Fig. S6: SNP-tree containing all samples, sequencing run replicates and EDTA-containing samples 46
4.7. Supplementary Fig. S7: Influence of EDTA on Nextera XT library preparation 47
4.8. Supplementary Fig. S8: Influence of EDTA on average number of reads uniquely mapping to the Sakai *E. coli* O157:H7 reference per million input reads 47
5. References **49**

1. Supplementary Methods

1.1. Statistical analyses of WGS data from EDTA containing samples

Fragmentation lengths of the library after Nextera XT preparation were descriptively analyzed using the average and standard deviation (s.d.) as center and variability measurements, respectively. The Mann-Whitney U test is a non-parametric test, used to compare the medians of two groups with limited sample sizes. This statistical analysis was used to: (i) Evaluate the effect of EDTA on the fragmentation step in Nextera XT library preparation; (ii) Assess the influence of EDTA on the median SNP number differences between the outbreak isolates, for each of the four DNA extraction kits; and (iii) Evaluate the influence EDTA on the number of reads uniquely mapping to the plasmid reference per million of input reads. The Kruskal-Wallis test is a non-parametric test, used to analyze the medians of three or more groups with limited sample sizes. This statistical test was used to: (i) Evaluate the effect of EDTA removal on the median SNP number differences between the outbreak isolates for the DNeasy Blood & Tissue kit.

2. Supplementary Notes

2.1. Investigation on the ambiguous serotyping

Ambiguous O-antigen genotyping results with BLAST+ assembly-based gene detection were only obtained in five out of 88 (or 5.07 % of the) samples and sequencing run replicates. For these five, serotyping was repeated using direct read mapping. Using thresholds of minimum 60 % and 10 % gene coverage and sequence divergence, respectively, SRST2 only allowed O-typing for sample TIAC1151 (run 1) generated with the DNeasy Blood & Tissue kit by identifying allele 201 for both the *wzx* and *wzy* genes. Indeed, for all outbreak samples, *wxz* allele 201 and *wxy* allele 201 could be identified. The assemblies of the five samples (TIAC1151 with DNeasy Blood & Tissue kit (run 1), TIAC1165 with DNeasy UltraClean Microbial kit and sequenced in run 1 and 3, TIAC1169 with Genomic-tip 20/G in run 2, and TIAC1660 with Genomic-tip 20/G sequenced in run 2) were aligned against allele 201 of the *wzx* and *wzy* genes coding for the O157-type in the outbreak samples and against allele 115 of gene *wzx* that was called as O113-type for TIAC1660. For the regions of all contigs aligning to the respective *wzx* and *wzy* alleles, 100 % sequence identity was observed. In all these samples, the assemblies aligning to the respective *wzx* and/or *wzy* alleles were fragmented and a combination of two or more contigs was

observed, fully or partly covering the respective gene allele. The median sequencing depth in the regions of those contigs was 3.99 (IQR: 4.22), potentially explaining the broken assembly.

2.2. Investigation on the ambiguous detection of cgMLST alleles

Only a very limited number of cgMLST differences existed within the different isolates. These differences could be explained by wrongly called alleles in: (i) Locus *b3119* for sample TIAC1152 generated with the Genomic-tip 20/G (allele 610 with 98.96 % identity and 90.19 % coverage instead of allele 9); (ii) Locus *b3119* for sample TIAC1152 generated with the DNeasy UltraClean Microbial kit (allele 763 with 98.57 % identity and 96.33 % coverage instead of allele 9); (iii) Locus *b3119* for sample TIAC1165 generated with the DNeasy UltraClean Microbial kit (run 1; allele 610 with 99.29 % identity and 65.42 % coverage instead of allele 9); and (iv) Locus *b1113* (allele 832 with 98.24 % identity and 89.14 % coverage instead of allele 35) for sample TIAC1153 generated with the Easy-DNA Genomic DNA (gDNA) Purification kit. Therefore, the assemblies of these samples were aligned against locus *b3119* allele 9 that was called for all outbreak samples (TIAC1151, TIAC1152, TIAC1165, and TIAC1169) or locus *b1113* allele 35 that was called for all TIAC1153 samples processed with the different DNA extraction kits. The results indicated that 100 % sequence identity with the correct, respective allele 9 or 35, was found in all these samples. However, due to the fragmented assembly, an incorrect allele was reported with less sequence identity, but longer query coverage.

2.3. The influence of EDTA on WGS data quality, isolate characterization and relatedness between the isolates

Due to the existence of discussion about the presence of EDTA in DNA extracts for WGS purposes and its potential impact on the quality and content of the WGS data, we evaluated the impact of EDTA on library preparation, subsequent WGS and STEC characterization and phylogenetic relatedness.

The influence of EDTA in the DNA solution buffers of the GenElute Bacterial gDNA, the DNeasy Blood & Tissue, the MasterPure Complete DNA Purification and the Wizard gDNA Purification kits, and the removal of EDTA using the gDNA Clean & Concentrator kit from the DNA extracts obtained with the DNeasy Blood & Tissue kit, was evaluated at different levels of the WGS workflow for isolates TIAC1151, TIAC1165 and TIAC1660 (all sequenced in run 4). After Nextera XT library preparation, DNA fragment lengths were determined, allowing to investigate the potential influence of EDTA on DNA fragmentation. Results are shown in *Supplementary Fig. S7*. No significant impact of EDTA on DNA fragmentation could be determined (Mann Whitney U test, n: 8, α: 0.05, p-value: 1.00). Although it has been reported

previously that EDTA has an effect on metal-dependent enzyme activation[1], we did not observe an effect of the presence of EDTA on DNA fragmentation by the metal-dependent Tn5 transposase[2] during Nextera XT library preparation. One possible reason for the unaffected enzyme activity, despite the presence of 0.5 or 1.0 mM EDTA, is the multiple DNA dilution and washing steps in the Nextera XT library preparation and MiSeq sequencing protocols that potentially decrease EDTA concentrations to levels too low to impact enzyme activity. Indeed, it has been demonstrated previously that inhibitory effects of EDTA on enzyme activity could only be observed when EDTA is in excess over the divalent cations that are required for enzyme activation[3].

The samples generated with the four sample preparation kits and DNA elution or rehydration in the supplied buffer containing EDTA were all sequenced in a fourth sequencing run. The median sequencing depth per sample in the fourth run was in the same range compared to when the DNA was prepared with the four DNA extraction kits but eluted in Tris-HCl, indicating that the presence of 0.5 mM or 1.0 mM EDTA did not affect the obtained median sequencing depth. All WGS quality control checks were met by all datasets, independent of the presence of EDTA or other confounding factors in the DNA extracts.

For almost all samples, results for serotyping, virulence and AMR genotyping per sample were identical to those obtained for each corresponding sample from which the DNA was dissolved in the absence of EDTA, except for two samples (TIAC1151 and TIAC1660) generated with the Wizard gDNA Purification kit. In these samples, the *tet41* AMR gene was detected as imperfect hit (for sample TIAC1151: 90.93 % query identity and 71.95 % query coverage, for sample TIAC1660: 90.81 % query identity and 74.44 % query coverage) using BLAST+ that was never detected in the respective samples generated with the other kits. Because the mapping depth in both contigs was very low (for sample TIAC1151: 0.87, for sample TIAC1660: 0.72) and because direct read mapping using SRST2 did not detect *tet41* in both of the samples, the detected *tet41* gene was, therefore, only detected with low confidence and is likely a false positive hit as a result of assembly issues of the respective contigs in both samples. Moreover, STEC characterization results were independent from the EDTA removal performed directly after DNeasy Blood & Tissue DNA preparation with elution in the supplied buffer (0.5 mM EDTA). The previously described effect of issues with assembly-based BLAST+ detection of some plasmid-encoded virulence genes (see main manuscript) was not observed for the three samples (TIAC1151, TIAC1165 and TIAC1660) generated with the DNeasy Blood & Tissue or the Wizard gDNA Purification kits and

eluted in the EDTA-containing buffer (*Supplementary Fig. S3*). The numbers of reads per million input reads mapping against the plasmid reference were irrespective of the presence of EDTA in the DNA extract, as depicted in *Supplementary Fig. S8*.

Exactly the same phylogenetic relationships as described previously (see main manuscript) between samples were retrieved based on both cgMLST (*Supplementary Fig. S5*) and SNP (*Supplementary Fig. S6*), irrespective of the presence of EDTA or its removal. Indeed, for each of the four DNA preparation kits, no SNP differences were identified when comparing the SNP profiles of TIAC1151 or TIAC1165 for which the DNA was eluted in the EDTA-containing buffer, or in 10 mM Tris-HCl (pH 8.5). For the GenElute Bacterial gDNA (p-value: 3.28×10^{-1}), the MasterPure Complete DNA Purification (p-value: 3.28×10^{-1}), and the Wizard gDNA Purification (p-value: 3.28×10^{-1}) kits, the number of SNPs compared to the Sakai *E. coli* O157:H7 reference genome between the outbreak samples was independent from the presence of EDTA, see *Supplementary Table S10* (Mann-Whitney U test, n: 12, α: 0.05). Moreover, for the DNeasy Blood & Tissue kit, neither the presence of EDTA nor the extra cleaning step affected the obtained SNP profiles (Kruskal Wallis, n: 18, α: 0.05, p-value: 5.14×10^{-1}).

Conclusively, the presence of EDTA did not affect the performance of isolate characterization, nor the retrieved relatedness between the samples. However, since false positive assembly-based detection of the AMR *tet41* gene was observed in samples generated with the Wizard gDNA Purification kit, caution must be taken for assembly-based STEC characterization if this kit is used and its DNA is rehydrated in the EDTA-containing buffer. We therefore recommend using direct read mapping with SRST2 for STEC characterization. Conclusively, the presence of EDTA in concentrations up to 1.0 mM in DNA extracts is no problem for STEC characterization and determining the relatedness between isolates when using the Nextera XT library preparation kit. Besides, the presence of EDTA in the DNA extract can even be regarded as advantageous with regard to its function in DNA preservation.

3. Supplementary Tables

3.1. Supplementary Table S1: Summary of selected DNA extraction kits for evaluation of EDTA influences

Supplementary Table S1 The selection of kits for the evaluation of EDTA presence, together with their characteristics and performance

DNA extraction kit	Accompanying DNA solution buffer kit / 10 mM Tris-HCl (pH 8.5)	Average DNA yield (µg/ml culture) ± s.d.	Average DNA concentration (ng/µl) ± s.d.	DNA purity (Average ± s.d.)		Length range of fragments (kb)	Average DIN*** ± s.d.
				A260/280	A260/230		
DNeasy Blood & Tissue	Buffer AE (0.5 mM EDTA)	2.62 ± 0.90*	26.17 ± 9.01*	1.85 ± 0.05*	1.57 ± 0.25*	[22.01,>60.00]*	7.40 ± 0.31*
	Buffer AE (0.5 mM EDTA) before DNA clean-up	3.31 ± 0.59**	33.08 ± 5.90**	1.82 ± 0.04**	1.47 ± 0.32**	[25.49,50.82]**	7.53 ± 0.29**
	10 mM Tris-HCl after DNA clean-up	2.70 ± 0.60**	26.96 ± 6.02**	1.91 ± 0.02**	2.08 ± 0.17**	[17.39,24.56]**	7.11 ± 0.26**
GenElute Bacterial Genomic DNA	Elution Solution (0.5mM EDTA)	5.57 ± 1.49*	34.83 ± 6.43*	1.85 ± 0.02*	2.11 ± 0.14*	[39.84,>60.00]*	8.78 ± 0.35*
	10 mM Tris-HCl (pH 8.5)	5.53 ± 0.95*	33.16 ± 5.70*	1.91 ± 0.02*	2.19 ± 0.13*	[23.37,51.59]*	9.13 ± 0.18*
MasterPure Complete DNA Purification	TE Buffer (1.0 mM EDTA)	2.32 ± 0.73*	34.07 ± 10.07*	1.85 ± 0.05*	1.73 ± 0.34*	[12.00,>60.00]*	8.94 ± 0.26*
	10 mM Tris-HCl (pH 8.5)	2.67 ± 0.53*	38.14 ± 7.51*	1.87 ± 0.03*	1.79 ± 0.21*	[58.58,>60.00]*	9.53 ± 0.21*
Wizard Genomic DNA Purification	DNA Rehydration Solution (1.0 mM EDTA)	2.30 ± 0.69*	23.04 ± 6.92*	1.88 ± 0.04*	2.29 ± 0.46*	[37.85,>60.00]*	9.06 ± 0.46*
	10 mM Tris-HCl (pH 8.5)	2.06 ± 0.65*	22.66 ± 4.55*	1.94 ± 0.03*	2.10 ± 0.09*	>60.00*	9.40 ± 0.34*

*The number of cells used as starting material ranged from 7.36 x 10^8 ± 8.56 x 10^7 per ml, as estimated by measuring Optical Density at 600nm (OD600). All averages and ranges were calculated from the three replicates of the seven DNA extracts. Both DNA concentration and yield are shown since the applied workflows accompanying the kits differed in recommended DNA elution/rehydration volume. Fragment lengths are shown as ranges because the TapeStation Genomic DNA ScreenTape only gives exact measurements until 60 kb. Only results relevant to the presence of EDTA in the DNA extracts are shown. For comparison with the results on DNA elution/rehydration in 10 mM Tris-HCl (pH 8.5), consult Table 1 in the main manuscript. *Averages calculated from the three replicates of the seven DNA extracts; **Averages calculated from the seven DNA extracts; ***DIN: DNA Integrity Number, ranging from 1.00 for highly degraded to 10.00 for highly intact DNA; Abbreviations: Next Generation Sequencing (NGS), High Molecular Weight (HMW)*

3.2. Supplementary table S2: PCR conditions and primer sequences for detection of plasmid-specific genes

Supplementary Table S2 PCR conditions and primer sequences for detection of plasmid-specific genes

a	Targeted sequence	Primer name	Primer sequence (5' → 3')	Primer concentration (nM)	Product size (bp)	Tm primer (°C)*	Reference
Hemolysin (*ehxA*) – pO157 and pO113		ehxA-135-F	5'-CGTTAAGGAACAGGAGGTGTCAGTA-3'	80	142	64.70	Nielsen, E.M. *et al.* 2003[4]
		ehxA-135-R	5'-ATCATGTTTTCCGCCAATGAG-3'	400		63.00	
STEC autoagglutinating adhesin (*saa*) – pO113		saa-135-F	5'-TGCCGCTGGTATAATTTTTCG-3'	80	85	60.70	Nielsen, E.M. *et al.* 2003[4]
		saa-135-R	5'-AACGCCTGTTCCATGTTGTG-3'	400		65.00	
β-Glucuronidase (*uidA*) - chromosome		uidA-3-F	5'-GCAGTTTCATCAATCACCAC-3'	250	87	59.80	Barbau-Piednoir, E. *et al.*, 2018[5]
		uidA-3-R	5'-CTCCTACCGTACCTCGCATTAC-3'	250		63.70	

b	Step	Number of cycles		Temperature (°C)		Time (h:min:sec)	
		uidA	*ehxA* and *saa*	*uidA*	*ehxA* and *saa*	*uidA*	*ehxA* and *saa*
Initial activation		1	1	95	95	0:03:00	0:15:00
Amplification	Denaturation	40	30	95	95	0:00:30	0:00:10

	**Hybridization**			57 (Tm-5)	60 (Tm - 5)	0:00:30	0:00:30
	**Elongation**			72	72	0:01:00	0:00:30
**Elongation**		1	1	72	72	0:10:00	0:10:00
**Cooling**		1	1	4	4	∞	∞

*Details on the primer sets for each target sequence (S2.a) and the PCR program (S2.a) are shown. The reaction was performed in a final volume of 25 µl containing 100 ng in 5 µl of the appropriate template DNA, 1X DreamTaq PCR MasterMix (Thermo Fisher Scientific, Schwerte, Germany) and the appropriate concentration of each primer, prepared on ice. *Tm: Melting temperature*

3.3. Supplementary Table S3: Conventional PCR results

Supplementary Table S3 Results of the conventional PCR detecting plasmid-specific genes and chromosome-encoded **uidA** ***gene***

DNA extraction kit	Isolate	Serotype	Targeted sequence		
			uidA	***ehxA***	***saa***
**DNeasy Blood & Tissue**	TIAC1165	O157:H7	1	1	0
	TIAC1660	O113:H21	1	1	1
	Blank	-	0	0	0
**DNeasy UltraClean Microbial kit**	TIAC1165	O157:H7	1	1	0
	TIAC1660	O113:H21	1	1	1
	Blank	-	0	0	0
**Easy-DNA gDNA Purification kit**	TIAC1165	O157:H7	1	1	0
	TIAC1660	O113:H21	1	1	1
	Blank	-	0	0	0
**GenElute Bacterial gDNA kit**	TIAC1165	O157:H7	1	1	0
	TIAC1660	O113:H21	1	1	1
	Blank	-	0	0	0
**Genomic-tip 20/G**	TIAC1165	O157:H7	1	1	0
	TIAC1660	O113:H21	1	1	1
	Blank	-	0	0	0
**MasterPure Complete DNA Purification kit**	TIAC1165	O157:H7	1	1	0
	TIAC1660	O113:H21	1	1	1
	Blank	-	0	0	0
**NucliSENS miniMAG**	TIAC1165	O157:H7	1	1	0
	TIAC1660	O113:H21	1	1	1
	Blank	-	0	0	0
**Wizard gDNA Purification kit**	TIAC1165	O157:H7	1	1	0
	TIAC1660	O113:H21	1	1	1
	Blank	-	0	0	0
**Boiled DNA extract as PC**	TIAC1165	O157:H7	1	1	0
	TIAC1660	O113:H21	1	1	1
NTC		-	0	0	0

This table shows the results of the conventional PCR reaction to detect plasmid-encoded ehxA *and* saa *genes, and housekeeping gene* uidA *in the three-fold DNA extracts of TIAC1165 and 1660 processed with each DNA extraction kit. Presence (1) or absence (0) of the gene was identified after visualization with the D1000 ScreenTape and Reagent kit on the TapeStation 4200 instrument. The expected gene profiles for TIAC1165:* uidA +, ehxA + *and* saa -*; and TIAC1660:* uidA +, ehxA +, saa +*, were also confirmed by the boiled DNA extracts that served as positive control (PC). Identical results have been obtained for each of the three replicates*

of each DNA extract. The size of the PCR fragments was corresponding to the theoretical product size, mentioned in Supplementary Table S2. All Negative Template Controls (NTCs) or blanks tested negative for the presence of all genes.

3.4. Supplementary Table S4: Set-up of the sequencing runs

Supplementary Table S4 Set-up of the sequencing runs

Run	DNA extraction kit (EDTA or no EDTA in DNA solution buffer)	No. isolates	Genome size (Mb)	Total no. isolates / run	Theoretical output / run (Gb)	Obtained number of reads / run (Millions)
Run 1	DNeasy Blood & Tissue kit (no EDTA)	7	5	31	12.48	26.77
	DNeasy UltraClean Microbial kit (no EDTA)	7	5			
	Easy-DNA gDNA Purification kit (no EDTA)	7	5			
	GenElute Bacterial gDNA kit (no EDTA)	2*	5			
	Genomic-tip 20/G kit (no EDTA)	2*	5			
	MasterPure Complete DNA Purification kit (no EDTA)	2*	5			
	NucliSENS miniMag kit (no EDTA)	2*	5			
	Wizard gDNA Purification kit (no EDTA)	2*	5			
Run 2	DNeasy Blood & Tissue kit (no EDTA)	2*	5	31	12.48	24.24
	DNeasy UltraClean Microbial kit (no EDTA)	2*	5			
	Easy-DNA gDNA Purification kit (no EDTA)	2*	5			
	GenElute Bacterial gDNA kit (no EDTA)	7	5			
	Genomic-tip 20/G kit (no EDTA)	7	5			
	MasterPure Complete DNA Purification kit (no EDTA)	7	5			
	NucliSENS miniMag kit (no EDTA)	2*	5			
	Wizard gDNA Purification kit (no EDTA)	2*	5			
Run 3	DNeasy Blood & Tissue kit (no EDTA)	2*	5	33	13.24	23.76
	DNeasy UltraClean Microbial kit (no EDTA)	2*	5			
	Easy-DNA gDNA Purification kit (no EDTA)	2*	5			
	GenElute Bacterial gDNA kit (no EDTA)	2*	5			
	Genomic-tip 20/G kit (no EDTA)	2*	5			
	MasterPure Complete DNA Purification kit (no EDTA)	2*	5			
	NucliSENS miniMag kit (no EDTA)	7	5			
	Wizard gDNA Purification kit (no EDTA)	7	5			
	Unrelated to this study	7	5			
Run 4	DNeasy Blood & Tissue kit (0.5 mM EDTA)	3	5	36	11.84	23.94
	DNeasy Blood & Tissue kit (EDTA removed)	3	5			
	GenElute Bacterial gDNA kit (0.5 mM EDTA)	3	5			
	MasterPure Complete DNA Purification kit (1.0 mM EDTA)	3	5			
	Wizard gDNA Purification kit (1.0 mM EDTA)	3	5			
	Unrelated to this study	1	5			
	Unrelated to this study	20	3			

Each run contained libraries of a complete set of seven DNA extracts processed with three (run 1 and 2) or two (run 3) different DNA extraction kits. Moreover, the libraries that were prepared from the DNA of TIAC1151 and TIAC1165 obtained with each DNA extraction kit were subsequently sequenced in each run, so that each run contained two isolates (sequencing run replicates) prepared with each DNA extraction kit and that possible variability introduced by the sequencing run could be assessed. The three*

isolates (TIAC1151, TIAC1165 and TIAC1660) that have been processed with the four DNA extraction kits containing EDTA in their DNA solution buffers were sequenced in the fourth run. To achieve a required output of ~12.5 Gb in run 3 and 4, extra isolates unrelated to this study were complemented in these runs. The obtained number of reads in each run is also shown.

3.5. Supplementary Table S5: Read trimming statistics

Supplementary Table S5 Read trimming statistics

Sample<br>DNA extraction kit<br>Solution buffer	Library ID	Run	Total reads	Both reads surviving (%)	Forward read only surviving (%)	Reverse read only surviving (%)	Both reads dropped (%)
TIAC1151<br>DNeasy Blood & Tissue<br>10 mM Tris-HCl (pH 8.5)	Blood-TissueTIAC1151-TrisHCl	1	425634	84.83	12.72	0.62	1.83
TIAC1152<br>DNeasy Blood & Tissue<br>10 mM Tris-HCl (pH 8.5)	Blood-TissueTIAC1152-TrisHCl	1	609362	89.63	8.55	0.59	1.23
TIAC1153<br>DNeasy Blood & Tissue<br>10 mM Tris-HCl (pH 8.5)	Blood-TissueTIAC1153-TrisHCl	1	741619	93.12	5.02	0.92	0.93
TIAC1165<br>DNeasy Blood & Tissue<br>10 mM Tris-HCl (pH 8.5)	Blood-TissueTIAC1165-TrisHCl	1	774916	92.63	5.79	0.66	0.92
TIAC1169<br>DNeasy Blood & Tissue<br>10 mM Tris-HCl (pH 8.5)	Blood-TissueTIAC1169-TrisHCl	1	833484	91.15	7.16	0.59	1.10
TIAC1638<br>DNeasy Blood & Tissue<br>10 mM Tris-HCl (pH 8.5)	Blood-TissueTIAC1638-TrisHCl	1	902326	92.26	6.19	0.57	0.98
TIAC1660<br>DNeasy Blood & Tissue<br>10 mM Tris-HCl (pH 8.5)	Blood-TissueTIAC1660-TrisHCl	1	858887	93.39	5.02	0.73	0.86
TIAC1151<br>DNeasy Blood & Tissue<br>10 mM Tris-HCl (pH 8.5)	Blood-TissueTIAC-1151-TrisHCl	2	735313	91.03	7.39	0.59	1.00
TIAC1165<br>DNeasy Blood & Tissue<br>10 mM Tris-HCl (pH 8.5)	Blood-TissueTIAC-1165-TrisHCl	2	859572	90.80	7.10	0.90	1.20
TIAC1151<br>DNeasy Blood & Tissue<br>10 mM Tris-HCl (pH 8.5)	Blood-Tissue-TIAC-1151-TrisHCl	3	584994	91.85	6.30	0.72	1.13
TIAC1165<br>DNeasy Blood & Tissue<br>10 mM Tris-HCl (pH 8.5)	Blood-Tissue-TIAC-1165-TrisHCl	3	524112	92.87	4.98	0.97	1.17
TIAC1151<br>DNeasy Blood & Tissue<br>Buffer kit (0.5 mM EDTA)	DBTK-1151-buffer	4	595166	91.85	6.45	0.69	1.00

TIAC1165 DNeasy Blood & Tissue Buffer kit (0.5 mM EDTA)	DBTK-1165-buffer	4	541926	90.40	8.05	0.53	1.02
TIAC1660 DNeasy Blood & Tissue Buffer kit (0.5 mM EDTA)	DBTK-1660-buffer	4	945986	94.36	4.32	0.57	0.75
TIAC1151 DNeasy Blood & Tissue Cleanup - 10 mM tris-HCl (pH 8.5)	DBTK-1151-cleanup	4	456826	86.06	12.31	0.38	1.25
TIAC1165 DNeasy Blood & Tissue Cleanup - 10 mM tris-HCl (pH 8.5)	DBTK-1165-cleanup	4	681697	90.17	8.42	0.42	0.99
TIAC1660 DNeasy Blood & Tissue Cleanup - 10 mM tris-HCl (pH 8.5)	DBTK-1660-cleanup	4	684572	91.13	7.40	0.48	0.99
TIAC1151 GenElute Bacterial gDNA 10 mM Tris-HCl (pH 8.5)	GenEluteTIAC-1151-TrisHCl	2	686048	91.33	7.07	0.59	1.02
TIAC1152 GenElute Bacterial gDNA 10 mM Tris-HCl (pH 8.5)	GenEluteTIAC-1152-TrisHCl	2	525630	91.75	6.69	0.61	0.96
TIAC1153 GenElute Bacterial gDNA 10 mM Tris-HCl (pH 8.5)	GenEluteTIAC-1153-TrisHCl	2	618371	92.81	5.51	0.75	0.92
TIAC1165 GenElute Bacterial gDNA 10 mM Tris-HCl (pH 8.5)	GenEluteTIAC-1165-TrisHCl	2	561727	88.68	9.45	0.63	1.25
TIAC1169 GenElute Bacterial gDNA 10 mM Tris-HCl (pH 8.5)	GenEluteTIAC-1169-TrisHCl	2	816755	91.49	6.94	0.58	0.99
TIAC1638 GenElute Bacterial gDNA 10 mM Tris-HCl (pH 8.5)	GenEluteTIAC-1638-TrisHCl	2	644031	89.15	9.28	0.47	1.09
TIAC1660 GenElute Bacterial gDNA 10 mM Tris-HCl (pH 8.5)	GenEluteTIAC-1660-TrisHCl	2	670252	90.79	7.49	0.65	1.07
TIAC1151 GenElute Bacterial gDNA 10 mM Tris-HCl (pH 8.5)	GenElute-TIAC1151-TrisHCl	1	832350	91.75	6.52	0.70	1.04
TIAC1165 GenElute Bacterial gDNA 10 mM Tris-HCl (pH 8.5)	GenElute-TIAC1165-TrisHCl	1	633310	92.09	5.94	0.92	1.06
TIAC1151 GenElute Bacterial gDNA 10 mM Tris-HCl (pH 8.5)	GenElute-TIAC-1151-TrisHCl	3	444752	90.23	7.75	0.69	1.33

TIAC1165 GenElute Bacterial gDNA 10 mM Tris-HCl (pH 8.5)	GenElute-TIAC-1165-TrisHCl	3	530002	92.46	5.76	0.70	1.09
TIAC1151 GenElute Bacterial gDNA Buffer kit (0.5 mM EDTA)	GBGDK-1151-buffer	4	647544	91.27	7.10	0.56	1.07
TIAC1165 GenElute Bacterial gDNA Buffer kit (0.5 mM EDTA)	GBGDK-1165-buffer	4	567315	90.86	7.66	0.49	0.98
TIAC1660 GenElute Bacterial gDNA Buffer kit (0.5 mM EDTA)	GBGDK-1660-buffer	4	492822	91.59	6.87	0.58	0.96
TIAC1151 Easy-DNA gDNA Purification 10 mM Tris-HCl (pH 8.5)	Easy-DNATIAC1151-2	1	807389	90.61	7.43	0.75	1.21
TIAC1152 Easy-DNA gDNA Purification 10 mM Tris-HCl (pH 8.5)	Easy-DNATIAC1152-2	1	792171	90.38	7.88	0.57	1.17
TIAC1153 Easy-DNA gDNA Purification 10 mM Tris-HCl (pH 8.5)	Easy-DNATIAC1153-2	1	408324	90.92	5.87	1.92	1.29
TIAC1165 Easy-DNA gDNA Purification 10 mM Tris-HCl (pH 8.5)	Easy-DNATIAC1165-2	1	857729	92.65	5.58	0.82	0.95
TIAC1169 Easy-DNA gDNA Purification 10 mM Tris-HCl (pH 8.5)	Easy-DNATIAC1169-2	1	685449	91.95	6.15	0.85	1.06
TIAC1638 Easy-DNA gDNA Purification 10 mM Tris-HCl (pH 8.5)	Easy-DNATIAC1638-2	1	755900	89.56	8.66	0.53	1.25
TIAC1660 Easy-DNA gDNA Purification 10 mM Tris-HCl (pH 8.5)	Easy-DNATIAC1660-2	1	678303	88.78	9.37	0.55	1.31
TIAC1151 Easy-DNA gDNA Purification 10 mM Tris-HCl (pH 8.5)	EasyDNATIAC-1151-2	2	849966	91.98	6.38	0.67	0.97
TIAC1165 Easy-DNA gDNA Purification 10 mM Tris-HCl (pH 8.5)	EasyDNATIAC-1165-2	2	682710	87.97	10.15	0.57	1.31
TIAC1151 Easy-DNA gDNA Purification 10 mM Tris-HCl (pH 8.5)	Easy-DNA-TIAC-1151-2	3	705898	93.48	4.92	0.69	0.91
TIAC1165 Easy-DNA gDNA Purification 10 mM Tris-HCl (pH 8.5)	Easy-DNA-TIAC-1165-2	3	571716	92.36	5.85	0.68	1.10

TIAC1151<br>Genomic-tip 20/G<br>10 mM Tris-HCl (pH 8.5)	gTip20TIAC-1151-1	2	549241	89.90	8.44	0.56	1.10
TIAC1152<br>Genomic-tip 20/G<br>10 mM Tris-HCl (pH 8.5)	gTip20TIAC-1152-1	2	448903	89.02	9.23	0.55	1.20
TIAC1153<br>Genomic-tip 20/G<br>10 mM Tris-HCl (pH 8.5)	gTip20TIAC-1153-1	2	635343	91.10	7.34	0.59	0.97
TIAC1165<br>Genomic-tip 20/G<br>10 mM Tris-HCl (pH 8.5)	gTip20TIAC-1165-1	2	488304	90.70	7.27	0.87	1.16
TIAC1169<br>Genomic-tip 20/G<br>10 mM Tris-HCl (pH 8.5)	gTip20TIAC-1169-1	2	707090	90.45	7.88	0.61	1.06
TIAC1638<br>Genomic-tip 20/G<br>10 mM Tris-HCl (pH 8.5)	gTip20TIAC-1638-1	2	501042	92.29	4.94	1.71	1.06
TIAC1660<br>Genomic-tip 20/G<br>10 mM Tris-HCl (pH 8.5)	gTip20TIAC-1660-1	2	683965	88.97	9.37	0.52	1.15
TIAC1151<br>Genomic-tip 20/G<br>10 mM Tris-HCl (pH 8.5)	gTip20-TIAC1151-1	1	773365	90.20	7.87	0.65	1.27
TIAC1165<br>Genomic-tip 20/G<br>10 mM Tris-HCl (pH 8.5)	gTip20-TIAC1165-1	1	538621	87.02	10.85	0.58	1.54
TIAC1151<br>Genomic-tip 20/G<br>10 mM Tris-HCl (pH 8.5)	gTip-20-TIAC-1151-1	3	558927	93.27	5.10	0.66	0.97
TIAC1165<br>Genomic-tip 20/G<br>10 mM Tris-HCl (pH 8.5)	gTip-20-TIAC-1165-1	3	537953	93.05	5.19	0.73	1.04
TIAC1151<br>MasterPure Complete<br>DNA Purification<br>10 mM Tris-HCl (pH 8.5)	MasterPureTIAC-<br>1151-TrisHCl	2	786257	91.30	7.05	0.62	1.03
TIAC1152<br>MasterPure Complete<br>DNA Purification<br>10 mM Tris-HCl (pH 8.5)	MasterPureTIAC-<br>1152-TrisHCl	2	713787	92.10	6.45	0.56	0.89
TIAC1153<br>MasterPure Complete<br>DNA Purification<br>10 mM Tris-HCl (pH 8.5)	MasterPureTIAC-<br>1153-TrisHCl	2	617451	89.46	8.70	0.65	1.19
TIAC1165<br>MasterPure Complete<br>DNA Purification<br>10 mM Tris-HCl (pH 8.5)	MasterPureTIAC-<br>1165-TrisHCl	2	492315	89.43	8.83	0.59	1.14

TIAC1169 MasterPure Complete DNA Purification 10 mM Tris-HCl (pH 8.5)	MasterPureTIAC-1169-TrisHCl	2	621465	91.54	6.53	0.85	1.07
TIAC1638 MasterPure Complete DNA Purification 10 mM Tris-HCl (pH 8.5)	MasterPureTIAC-1638-TrisHCl	2	563570	88.44	9.76	0.57	1.23
TIAC1660 MasterPure Complete DNA Purification 10 mM Tris-HCl (pH 8.5)	MasterPureTIAC-1660-TrisHCl	2	739828	90.69	7.68	0.59	1.04
TIAC1151 MasterPure Complete DNA Purification 10 mM Tris-HCl (pH 8.5)	MasterPure-TIAC1151-TrisHCl	1	572885	86.38	11.57	0.47	1.58
TIAC1165 MasterPure Complete DNA Purification 10 mM Tris-HCl (pH 8.5)	MasterPure-TIAC1165-TrisHCl	1	531907	89.45	8.29	0.90	1.36
TIAC1151 MasterPure Complete DNA Purification 10 mM Tris-HCl (pH 8.5)	MasterPure-TIAC-1151-TrisHCl	3	511733	92.06	6.11	0.70	1.13
TIAC1165 MasterPure Complete DNA Purification 10 mM Tris-HCl (pH 8.5)	MasterPure-TIAC-1165-TrisHCl	3	419677	90.89	7.03	0.75	1.32
TIAC1151 MasterPure Complete DNA Purification Buffer kit (1.0 mM EDTA)	MCDPK-1151-buffer	4	722739	91.24	7.45	0.42	0.88
TIAC1165 MasterPure Complete DNA Purification Buffer kit (1.0 mM EDTA)	MCDPK-1165-buffer	4	639960	91.30	7.32	0.44	0.93
TIAC1660 MasterPure Complete DNA Purification Buffer kit (1.0 mM EDTA)	MCDPK-1660-buffer	4	387341	89.33	8.95	0.53	1.19
TIAC1151 Wizard gDNA Purification 10 mM Tris-HCl (pH 8.5)	Wizard-TIAC-1151-TrisHCl	3	448196	92.16	6.05	0.70	1.09
TIAC1152 Wizard gDNA Purification 10 mM Tris-HCl (pH 8.5)	Wizard-TIAC-1152-TrisHCl	3	402227	91.40	6.62	0.75	1.23
TIAC1153 Wizard gDNA Purification 10 mM Tris-HCl (pH 8.5)	Wizard-TIAC-1153-TrisHCl	3	603037	93.09	5.23	0.68	1.00
TIAC1165 Wizard gDNA Purification 10 mM Tris-HCl (pH 8.5)	Wizard-TIAC-1165-TrisHCl	3	368907	92.05	5.93	0.79	1.22
TIAC1169 Wizard gDNA Purification 10 mM Tris-HCl (pH 8.5)	Wizard-TIAC-1169-TrisHCl	3	540866	89.04	8.88	0.67	1.41

TIAC1638 Wizard gDNA Purification 10 mM Tris-HCl (pH 8.5)	Wizard-TIAC-1638-TrisHCl	3	514372	93.41	4.35	1.09	1.15
TIAC1660 Wizard gDNA Purification 10 mM Tris-HCl (pH 8.5)	Wizard-TIAC-1660-TrisHCl	3	404447	91.56	6.50	0.76	1.18
TIAC1151 Wizard gDNA Purification 10 mM Tris-HCl (pH 8.5)	Wizard-TIAC1151-TrisHCl	1	599660	90.78	7.37	0.70	1.14
TIAC1165 Wizard gDNA Purification 10 mM Tris-HCl (pH 8.5)	Wizard-TIAC1165-TrisHCl	1	487916	88.51	9.29	0.73	1.48
TIAC1151 Wizard gDNA Purification 10 mM Tris-HCl (pH 8.5)	WizardTIAC-1151-TrisHCl	2	619549	88.84	9.30	0.65	1.22
TIAC1165 Wizard gDNA Purification 10 mM Tris-HCl (pH 8.5)	WizardTIAC-1165-TrisHCl	2	514941	85.04	12.83	0.54	1.59
TIAC1151 Wizard gDNA Purification Buffer kit (0.5 mM EDTA)	WGDPK-1151-buffer	4	555615	90.79	7.53	0.59	1.10
TIAC1165 Wizard gDNA Purification Buffer kit (0.5 mM EDTA)	WGDPK-1165-buffer	4	586666	89.04	9.24	0.51	1.21
TIAC1660 Wizard gDNA Purification Buffer kit (0.5 mM EDTA)	WGDPK-1660-buffer	4	744918	92.09	6.51	0.51	0.90
TIAC1151 UltraClean Microbial DNA isolation Buffer kit	UltraCleanTIAC1151-3	1	1033500	92.38	6.06	0.61	0.95
TIAC1152 UltraClean Microbial DNA isolation Buffer kit	UltraCleanTIAC1152-3	1	687773	90.09	7.93	0.71	1.27
TIAC1153 UltraClean Microbial DNA isolation Buffer kit	UltraCleanTIAC1153-3	1	681744	92.56	5.61	0.85	0.98
TIAC1165 UltraClean Microbial DNA isolation Buffer kit	UltraCleanTIAC1165-3	1	764035	92.13	5.76	1.02	1.08
TIAC1169 UltraClean Microbial DNA isolation Buffer kit	UltraCleanTIAC1169-3	1	1287955	89.86	8.44	0.53	1.17
TIAC1638 UltraClean Microbial DNA isolation Buffer kit	UltraCleanTIAC1638-3	1	848708	90.16	7.98	0.66	1.21

TIAC1660 UltraClean Microbial DNA isolation Buffer kit	UltraCleanTIAC1660-3	1	993849	91.15	7.30	0.54	1.02
TIAC1151 UltraClean Microbial DNA isolation Buffer kit	UltraCleanTIAC-1151-3	2	595373	89.69	8.70	0.53	1.08
TIAC1165 UltraClean Microbial DNA isolation Buffer kit	UltraCleanTIAC-1165-3	2	604829	91.72	6.55	0.73	1.00
TIAC1151 UltraClean Microbial DNA isolation Buffer kit	UltraClean-TIAC-1151-3	3	523915	93.63	4.86	0.62	0.89
TIAC1165 UltraClean Microbial DNA isolation Buffer kit	UltraClean-TIAC-1165-3	3	654035	92.46	5.85	0.65	1.04
TIAC1151 NucliSENS miniMag Buffer kit	MiniMAG-TIAC-1151-3	3	476740	92.99	5.05	0.82	1.15
TIAC1152 NucliSENS miniMag Buffer kit	MiniMAG-TIAC-1152-3	3	616106	92.70	5.57	0.69	1.04
TIAC1153 NucliSENS miniMag Buffer kit	MiniMAG-TIAC-1153-3	3	599331	93.61	4.66	0.73	0.99
TIAC1165 NucliSENS miniMag Buffer kit	MiniMAG-TIAC-1165-3	3	490290	91.54	6.67	0.63	1.15
TIAC1169 NucliSENS miniMag Buffer kit	MiniMAG-TIAC-1169-3	3	467486	92.79	5.44	0.68	1.09
TIAC1638 NucliSENS miniMag Buffer kit	MiniMAG-TIAC-1638-3	3	449791	90.82	7.29	0.69	1.20
TIAC1660 NucliSENS miniMag Buffer kit	MiniMAG-TIAC-1660-3	3	627028	94.06	4.12	0.85	0.97
TIAC1151 NucliSENS miniMag Buffer kit	miniMAG-TIAC-1151-3	1	724730	92.21	6.15	0.63	1.01
TIAC1165 NucliSENS miniMag Buffer kit	miniMAG-TIAC-1165-3	1	572350	89.50	8.36	0.77	1.37
TIAC1151 NucliSENS miniMag Buffer kit	miniMAGTIAC-1151-3	2	749474	91.26	7.15	0.59	1.00

TIAC1165<br>NucliSENS miniMag<br>Buffer kit	miniMAGTIAC-1165-3	2	680980	91.74	6.58	0.66	1.02

In the first column, the sample name, used DNA extraction kit and the applied buffer for DNA rehydration or elution is mentioned. The second column contains the identity of each library as also indicated as the name of the WGS data in NCBI SRA. The following, consecutive columns list the run name, the percentage of total reads where both members of the read pair passed trimming, the percentage of total reads where only the forward read passed read trimming, the percentage of total reads where only the reverse read passed read trimming, and the percentage of total reads where both read pair members were dropped in the trimming step.

3.6. Supplementary Table S6: Assembly statistics

Supplementary Table S6 Assembly statistics

**Sample<br>DNA extraction kit<br>Solution buffer**	**Library ID**	**Run**	**N50**	**Number of contigs**	**Number of contigs >1000bp**
TIAC1151<br>DNeasy Blood & Tissue<br>10 mM Tris-HCl (pH 8.5)	Blood-TissueTIAC1151-TrisHCl	1	89551	278	209
TIAC1152<br>DNeasy Blood & Tissue<br>10 mM Tris-HCl (pH 8.5)	Blood-TissueTIAC1152-TrisHCl	1	149903	175	125
TIAC1153<br>DNeasy Blood & Tissue<br>10 mM Tris-HCl (pH 8.5)	Blood-TissueTIAC1153-TrisHCl	1	92385	256	191
TIAC1165<br>DNeasy Blood & Tissue<br>10 mM Tris-HCl (pH 8.5)	Blood-TissueTIAC1165-TrisHCl	1	149919	189	137
TIAC1169<br>DNeasy Blood & Tissue<br>10 mM Tris-HCl (pH 8.5)	Blood-TissueTIAC1169-TrisHCl	1	111103	234	174
TIAC1638<br>DNeasy Blood & Tissue<br>10 mM Tris-HCl (pH 8.5)	Blood-TissueTIAC1638-TrisHCl	1	72306	294	223
TIAC1660<br>DNeasy Blood & Tissue<br>10 mM Tris-HCl (pH 8.5)	Blood-TissueTIAC1660-TrisHCl	1	106371	145	118
TIAC1151<br>DNeasy Blood & Tissue<br>10 mM Tris-HCl (pH 8.5)	Blood-TissueTIAC-1151-TrisHCl	2	134306	199	149
TIAC1165<br>DNeasy Blood & Tissue<br>10 mM Tris-HCl (pH 8.5)	Blood-TissueTIAC-1165-TrisHCl	2	100158	220	167
TIAC1151<br>DNeasy Blood & Tissue<br>10 mM Tris-HCl (pH 8.5)	Blood-Tissue-TIAC-1151-TrisHCl	3	148373	221	131
TIAC1165<br>DNeasy Blood & Tissue<br>10 mM Tris-HCl (pH 8.5)	Blood-Tissue-TIAC-1165-TrisHCl	3	142785	208	142

TIAC1151<br>DNeasy Blood & Tissue<br>Buffer kit (0.5 mM EDTA)	DBTK-1151-buffer	4	148373	580	141
TIAC1165<br>DNeasy Blood & Tissue<br>Buffer kit (0.5 mM EDTA)	DBTK-1165-buffer	4	149903	490	139
TIAC1660<br>DNeasy Blood & Tissue<br>Buffer kit (0.5 mM EDTA)	DBTK-1660-buffer	4	154978	573	107
TIAC1151<br>DNeasy Blood & Tissue<br>Cleanup - 10 mM tris-HCl (pH 8.5)	DBTK-1151-cleanup	4	148626	397	135
TIAC1165<br>DNeasy Blood & Tissue<br>Cleanup - 10 mM tris-HCl (pH 8.5)	DBTK-1165-cleanup	4	149903	620	152
TIAC1660<br>DNeasy Blood & Tissue<br>Cleanup - 10 mM tris-HCl (pH 8.5)	DBTK-1660-cleanup	4	154978	454	98
TIAC1151<br>GenElute Bacterial gDNA<br>10 mM Tris-HCl (pH 8.5)	GenEluteTIAC-1151-<br>TrisHCl	2	145145	189	141
TIAC1152<br>GenElute Bacterial gDNA<br>10 mM Tris-HCl (pH 8.5)	GenEluteTIAC-1152-<br>TrisHCl	2	149903	163	119
TIAC1153<br>GenElute Bacterial gDNA<br>10 mM Tris-HCl (pH 8.5)	GenEluteTIAC-1153-<br>TrisHCl	2	168801	196	136
TIAC1165<br>GenElute Bacterial gDNA<br>10 mM Tris-HCl (pH 8.5)	GenEluteTIAC-1165-<br>TrisHCl	2	149903	160	114
TIAC1169<br>GenElute Bacterial gDNA<br>10 mM Tris-HCl (pH 8.5)	GenEluteTIAC-1169-<br>TrisHCl	2	149919	170	123
TIAC1638<br>GenElute Bacterial gDNA<br>10 mM Tris-HCl (pH 8.5)	GenEluteTIAC-1638-<br>TrisHCl	2	142785	201	145
TIAC1660<br>GenElute Bacterial gDNA<br>10 mM Tris-HCl (pH 8.5)	GenEluteTIAC-1660-<br>TrisHCl	2	139624	125	99
TIAC1151<br>GenElute Bacterial gDNA<br>10 mM Tris-HCl (pH 8.5)	GenElute-TIAC1151-<br>TrisHCl	1	148373	196	138
TIAC1165<br>GenElute Bacterial gDNA<br>10 mM Tris-HCl (pH 8.5)	GenElute-TIAC1165-<br>TrisHCl	1	98015	247	192

TIAC1151<br>GenElute Bacterial gDNA<br>10 mM Tris-HCl (pH 8.5)	GenElute-TIAC-1151-TrisHCl	3	124514	197	148
TIAC1165<br>GenElute Bacterial gDNA<br>10 mM Tris-HCl (pH 8.5)	GenElute-TIAC-1165-TrisHCl	3	148373	193	148
TIAC1151<br>GenElute Bacterial gDNA<br>Buffer kit (0.5 mM EDTA)	GBGDK-1151-buffer	4	148373	686	148
TIAC1165<br>GenElute Bacterial gDNA<br>Buffer kit (0.5 mM EDTA)	GBGDK-1165-buffer	4	148373	551	139
TIAC1660<br>GenElute Bacterial gDNA<br>Buffer kit (0.5 mM EDTA)	GBGDK-1660-buffer	4	154978	358	103
TIAC1151<br>Easy-DNA gDNA Purification<br>10 mM Tris-HCl (pH 8.5)	Easy-DNATIAC1151-2	1	148626	210	153
TIAC1152<br>Easy-DNA gDNA Purification<br>10 mM Tris-HCl (pH 8.5)	Easy-DNATIAC1152-2	1	140033	216	158
TIAC1153<br>Easy-DNA gDNA Purification<br>10 mM Tris-HCl (pH 8.5)	Easy-DNATIAC1153-2	1	108049	257	191
TIAC1165<br>Easy-DNA gDNA Purification<br>10 mM Tris-HCl (pH 8.5)	Easy-DNATIAC1165-2	1	148626	212	153
TIAC1169<br>Easy-DNA gDNA Purification<br>10 mM Tris-HCl (pH 8.5)	Easy-DNATIAC1169-2	1	124420	235	170
TIAC1638<br>Easy-DNA gDNA Purification<br>10 mM Tris-HCl (pH 8.5)	Easy-DNATIAC1638-2	1	113515	229	166
TIAC1660<br>Easy-DNA gDNA Purification<br>10 mM Tris-HCl (pH 8.5)	Easy-DNATIAC1660-2	1	102559	137	112
TIAC1151<br>Easy-DNA gDNA Purification<br>10 mM Tris-HCl (pH 8.5)	EasyDNATIAC-1151-2	2	112026	244	186
TIAC1165<br>Easy-DNA gDNA Purification<br>10 mM Tris-HCl (pH 8.5)	EasyDNATIAC-1165-2	2	111089	231	170
TIAC1151<br>Easy-DNA gDNA Purification<br>10 mM Tris-HCl (pH 8.5)	Easy-DNA-TIAC-1151-2	3	149024	220	157

TIAC1165<br>Easy-DNA gDNA Purification<br>10 mM Tris-HCl (pH 8.5)	Easy-DNA-TIAC-1165-2	3	149903	222	152
TIAC1151<br>Genomic-tip 20/G<br>10 mM Tris-HCl (pH 8.5)	gTip20TIAC-1151-1	2	149903	168	122
TIAC1152<br>Genomic-tip 20/G<br>10 mM Tris-HCl (pH 8.5)	gTip20TIAC-1152-1	2	110594	229	177
TIAC1153<br>Genomic-tip 20/G<br>10 mM Tris-HCl (pH 8.5)	gTip20TIAC-1153-1	2	104853	240	175
TIAC1165<br>Genomic-tip 20/G<br>10 mM Tris-HCl (pH 8.5)	gTip20TIAC-1165-1	2	111061	223	166
TIAC1169<br>Genomic-tip 20/G<br>10 mM Tris-HCl (pH 8.5)	gTip20TIAC-1169-1	2	109245	248	188
TIAC1638<br>Genomic-tip 20/G<br>10 mM Tris-HCl (pH 8.5)	gTip20TIAC-1638-1	2	111111	245	180
TIAC1660<br>Genomic-tip 20/G<br>10 mM Tris-HCl (pH 8.5)	gTip20TIAC-1660-1	2	116607	136	105
TIAC1151<br>Genomic-tip 20/G<br>10 mM Tris-HCl (pH 8.5)	gTip20-TIAC1151-1	1	58511	302	235
TIAC1165<br>Genomic-tip 20/G<br>10 mM Tris-HCl (pH 8.5)	gTip20-TIAC1165-1	1	95735	264	203
TIAC1151<br>Genomic-tip 20/G<br>10 mM Tris-HCl (pH 8.5)	gTip-20-TIAC-1151-1	3	153441	179	130
TIAC1165<br>Genomic-tip 20/G<br>10 mM Tris-HCl (pH 8.5)	gTip-20-TIAC-1165-1	3	142785	183	136
TIAC1151<br>MasterPure Complete DNA Purification<br>10 mM Tris-HCl (pH 8.5)	MasterPureTIAC-1151-TrisHCl	2	124514	196	145
TIAC1152<br>MasterPure Complete DNA Purification<br>10 mM Tris-HCl (pH 8.5)	MasterPureTIAC-1152-TrisHCl	2	165273	168	115
TIAC1153<br>MasterPure Complete DNA Purification<br>10 mM Tris-HCl (pH 8.5)	MasterPureTIAC-1153-TrisHCl	2	148373	195	136

TIAC1165<br>MasterPure Complete DNA Purification<br>10 mM Tris-HCl (pH 8.5)	MasterPureTIAC-1165-TrisHCl	2	147821	178	130
TIAC1169<br>MasterPure Complete DNA Purification<br>10 mM Tris-HCl (pH 8.5)	MasterPureTIAC-1169-TrisHCl	2	136218	175	127
TIAC1638<br>MasterPure Complete DNA Purification<br>10 mM Tris-HCl (pH 8.5)	MasterPureTIAC-1638-TrisHCl	2	118067	199	151
TIAC1660<br>MasterPure Complete DNA Purification<br>10 mM Tris-HCl (pH 8.5)	MasterPureTIAC-1660-TrisHCl	2	139700	121	96
TIAC1151<br>MasterPure Complete DNA Purification<br>10 mM Tris-HCl (pH 8.5)	MasterPure-TIAC1151-TrisHCl	1	95735	256	195
TIAC1165<br>MasterPure Complete DNA Purification<br>10 mM Tris-HCl (pH 8.5)	MasterPure-TIAC1165-TrisHCl	1	92356	246	187
TIAC1151<br>MasterPure Complete DNA Purification<br>10 mM Tris-HCl (pH 8.5)	MasterPure-TIAC-1151-TrisHCl	3	124420	204	144
TIAC1165<br>MasterPure Complete DNA Purification<br>10 mM Tris-HCl (pH 8.5)	MasterPure-TIAC-1165-TrisHCl	3	107345	232	173
TIAC1151<br>MasterPure Complete DNA Purification<br>Buffer kit (1.0 mM EDTA)	MCDPK-1151-buffer	4	148626	627	144
TIAC1165<br>MasterPure Complete DNA Purification<br>Buffer kit (1.0 mM EDTA)	MCDPK-1165-buffer	4	148626	568	139
TIAC1660<br>MasterPure Complete DNA Purification<br>Buffer kit (1.0 mM EDTA)	MCDPK-1660-buffer	4	157238	303	94
TIAC1151<br>Wizard gDNA Purification<br>10 mM Tris-HCl (pH 8.5)	Wizard-TIAC-1151-TrisHCl	3	160709	178	127
TIAC1152<br>Wizard gDNA Purification<br>10 mM Tris-HCl (pH 8.5)	Wizard-TIAC-1152-TrisHCl	3	148373	186	133
TIAC1153<br>Wizard gDNA Purification<br>10 mM Tris-HCl (pH 8.5)	Wizard-TIAC-1153-TrisHCl	3	174422	205	141
TIAC1165<br>Wizard gDNA Purification<br>10 mM Tris-HCl (pH 8.5)	Wizard-TIAC-1165-TrisHCl	3	58806	322	254

TIAC1169<br>Wizard gDNA Purification<br>10 mM Tris-HCl (pH 8.5)	Wizard-TIAC-1169-TrisHCl	3	126566	219	138
TIAC1638<br>Wizard gDNA Purification<br>10 mM Tris-HCl (pH 8.5)	Wizard-TIAC-1638-TrisHCl	3	145145	197	132
TIAC1660<br>Wizard gDNA Purification<br>10 mM Tris-HCl (pH 8.5)	Wizard-TIAC-1660-TrisHCl	3	143442	131	101
TIAC1151<br>Wizard gDNA Purification<br>10 mM Tris-HCl (pH 8.5)	Wizard-TIAC1151-TrisHCl	1	136094	205	147
TIAC1165<br>Wizard gDNA Purification<br>10 mM Tris-HCl (pH 8.5)	Wizard-TIAC1165-TrisHCl	1	83437	263	205
TIAC1151<br>Wizard gDNA Purification<br>10 mM Tris-HCl (pH 8.5)	WizardTIAC-1151-TrisHCl	2	148373	173	126
TIAC1165<br>Wizard gDNA Purification<br>10 mM Tris-HCl (pH 8.5)	WizardTIAC-1165-TrisHCl	2	142785	192	143
TIAC1151<br>Wizard gDNA Purification<br>Buffer kit (0.5 mM EDTA)	WGDPK-1151-buffer	4	148791	467	130
TIAC1165<br>Wizard gDNA Purification<br>Buffer kit (0.5 mM EDTA)	WGDPK-1165-buffer	4	149903	531	139
TIAC1660<br>Wizard gDNA Purification<br>Buffer kit (0.5 mM EDTA)	WGDPK-1660-buffer	4	146170	1268	229
TIAC1151<br>UltraClean Microbial DNA isolation<br>Buffer kit	UltraCleanTIAC1151-3	1	135948	207	150
TIAC1152<br>UltraClean Microbial DNA isolation<br>Buffer kit	UltraCleanTIAC1152-3	1	92365	243	187
TIAC1153<br>UltraClean Microbial DNA isolation<br>Buffer kit	UltraCleanTIAC1153-3	1	95424	257	195
TIAC1165<br>UltraClean Microbial DNA isolation<br>Buffer kit	UltraCleanTIAC1165-3	1	54572	334	247
TIAC1169<br>UltraClean Microbial DNA isolation<br>Buffer kit	UltraCleanTIAC1169-3	1	142785	199	140

TIAC1638<br>UltraClean Microbial DNA isolation<br>Buffer kit	UltraCleanTIAC1638-3	1	117952	215	158
TIAC1660<br>UltraClean Microbial DNA isolation<br>Buffer kit	UltraCleanTIAC1660-3	1	157238	117	87
TIAC1151<br>UltraClean Microbial DNA isolation<br>Buffer kit	UltraCleanTIAC-1151-3	2	107847	205	154
TIAC1165<br>UltraClean Microbial DNA isolation<br>Buffer kit	UltraCleanTIAC-1165-3	2	142785	186	133
TIAC1151<br>UltraClean Microbial DNA isolation<br>Buffer kit	UltraClean-TIAC-1151-3	3	124420	231	168
TIAC1165<br>UltraClean Microbial DNA isolation<br>Buffer kit	UltraClean-TIAC-1165-3	3	71306	273	211
TIAC1151<br>NucliSENS miniMag<br>Buffer kit	MiniMAG-TIAC-1151-3	3	149919	193	129
TIAC1152<br>NucliSENS miniMag<br>Buffer kit	MiniMAG-TIAC-1152-3	3	149919	177	121
TIAC1153<br>NucliSENS miniMag<br>Buffer kit	MiniMAG-TIAC-1153-3	3	174422	194	135
TIAC1165<br>NucliSENS miniMag<br>Buffer kit	MiniMAG-TIAC-1165-3	3	148626	202	142
TIAC1169<br>NucliSENS miniMag<br>Buffer kit	MiniMAG-TIAC-1169-3	3	149919	191	135
TIAC1638<br>NucliSENS miniMag<br>Buffer kit	MiniMAG-TIAC-1638-3	3	107945	272	167
TIAC1660<br>NucliSENS miniMag<br>Buffer kit	MiniMAG-TIAC-1660-3	3	157238	126	92
TIAC1151<br>NucliSENS miniMag<br>Buffer kit	miniMAG-TIAC-1151-3	1	107937	208	152
TIAC1165<br>NucliSENS miniMag<br>Buffer kit	miniMAG-TIAC-1165-3	1	107589	205	152

TIAC1151<br>NucliSENS miniMag<br>Buffer kit	miniMAGTIAC-1151-3	2	148626	177	126
TIAC1165<br>NucliSENS miniMag<br>Buffer kit	miniMAGTIAC-1165-3	2	142785	177	129

In the first column, the sample name, used kit and the applied buffer for DNA rehydration or elution is mentioned. The second column contains the identity of each library as also indicated as the name of the WGS data in NCBI SRA. The third, fourth, fifth, and sixth columns list the run name, N50, number of contigs, and number of contigs > 1,000 bases, respectively.

3.7. Supplementary Table S7: Advanced quality control statistics

Supplementary Table S7 Advanced quality control statistics

Sample<br>DNA extraction kit<br>Solution buffer	Library ID	Run	Median sequencing depth against assembly	IQR Median sequencing depth against assembly	Mapping rate (%) against assembly	cgMLST loci detected (%) (Total: 2513 loci)
TIAC1151<br>DNeasy Blood & Tissue<br>10 mM Tris-HCl (pH 8.5)	Blood-TissueTIAC1151-TrisHCl	1	29	12	99.73	99.76
TIAC1152<br>DNeasy Blood & Tissue<br>10 mM Tris-HCl (pH 8.5)	Blood-TissueTIAC1152-TrisHCl	1	45	16	99.78	99.76
TIAC1153<br>DNeasy Blood & Tissue<br>10 mM Tris-HCl (pH 8.5)	Blood-TissueTIAC1153-TrisHCl	1	58	19	99.77	99.52
TIAC1165<br>DNeasy Blood & Tissue<br>10 mM Tris-HCl (pH 8.5)	Blood-TissueTIAC1165-TrisHCl	1	59	19	99.76	99.76
TIAC1169<br>DNeasy Blood & Tissue<br>10 mM Tris-HCl (pH 8.5)	Blood-TissueTIAC1169-TrisHCl	1	65	23	99.73	99.72
TIAC1638<br>DNeasy Blood & Tissue<br>10 mM Tris-HCl (pH 8.5)	Blood-TissueTIAC1638-TrisHCl	1	69	30	99.74	98.81
TIAC1660<br>DNeasy Blood & Tissue<br>10 mM Tris-HCl (pH 8.5)	Blood-TissueTIAC1660-TrisHCl	1	67	27	99.73	97.06
TIAC1151<br>DNeasy Blood & Tissue<br>10 mM Tris-HCl (pH 8.5)	Blood-TissueTIAC-1151-TrisHCl	2	58	21	99.73	99.76
TIAC1165<br>DNeasy Blood & Tissue<br>10 mM Tris-HCl (pH 8.5)	Blood-TissueTIAC-1165-TrisHCl	2	68	24	99.73	99.76
TIAC1151<br>DNeasy Blood & Tissue<br>10 mM Tris-HCl (pH 8.5)	Blood-Tissue-TIAC-1151-TrisHCl	3	45	16	99.63	99.76

TIAC1165 DNeasy Blood & Tissue 10 mM Tris-HCl (pH 8.5)	Blood-Tissue-TIAC-1165-TrisHCl	3	41	15	99.65	99.76
TIAC1151 DNeasy Blood & Tissue Buffer kit (0.5 mM EDTA)	DBTK-1151-buffer	4	44	19	99.02	99.76
TIAC1165 DNeasy Blood & Tissue Buffer kit (0.5 mM EDTA)	DBTK-1165-buffer	4	39	17	98.98	99.76
TIAC1660 DNeasy Blood & Tissue Buffer kit (0.5 mM EDTA)	DBTK-1660-buffer	4	63	31	99.17	97.06
TIAC1151 DNeasy Blood & Tissue Cleanup - 10 mM tris-HCl (pH 8.5)	DBTK-1151-cleanup	4	32	13	98.90	99.76
TIAC1165 DNeasy Blood & Tissue Cleanup - 10 mM tris-HCl (pH 8.5)	DBTK-1165-cleanup	4	49	19	99.01	99.72
TIAC1660 DNeasy Blood & Tissue Cleanup - 10 mM tris-HCl (pH 8.5)	DBTK-1660-cleanup	4	50	22	99.16	97.06
TIAC1151 GenElute Bacterial gDNA 10 mM Tris-HCl (pH 8.5)	GenEluteTIAC-1151-TrisHCl	2	53	18	99.74	99.76
TIAC1152 GenElute Bacterial gDNA 10 mM Tris-HCl (pH 8.5)	GenEluteTIAC-1152-TrisHCl	2	41	14	99.74	99.76
TIAC1153 GenElute Bacterial gDNA 10 mM Tris-HCl (pH 8.5)	GenEluteTIAC-1153-TrisHCl	2	48	17	99.74	99.52
TIAC1165 GenElute Bacterial gDNA 10 mM Tris-HCl (pH 8.5)	GenEluteTIAC-1165-TrisHCl	2	41	15	99.75	99.76
TIAC1169 GenElute Bacterial gDNA 10 mM Tris-HCl (pH 8.5)	GenEluteTIAC-1169-TrisHCl	2	63	20	99.76	99.72
TIAC1638 GenElute Bacterial gDNA 10 mM Tris-HCl (pH 8.5)	GenEluteTIAC-1638-TrisHCl	2	48	20	99.75	98.81
TIAC1660 GenElute Bacterial gDNA 10 mM Tris-HCl (pH 8.5)	GenEluteTIAC-1660-TrisHCl	2	52	22	99.75	97.06
TIAC1151 GenElute Bacterial gDNA 10 mM Tris-HCl (pH 8.5)	GenElute-TIAC1151-TrisHCl	1	63	22	99.74	99.76

TIAC1165<br>GenElute Bacterial gDNA<br>10 mM Tris-HCl (pH 8.5)	GenElute-TIAC1165-TrisHCl	1	48	17	99.77	99.76
TIAC1151<br>GenElute Bacterial gDNA<br>10 mM Tris-HCl (pH 8.5)	GenElute-TIAC-1151-TrisHCl	3	33	13	99.74	99.76
TIAC1165<br>GenElute Bacterial gDNA<br>10 mM Tris-HCl (pH 8.5)	GenElute-TIAC-1165-TrisHCl	3	40	16	99.74	99.76
TIAC1151<br>GenElute Bacterial gDNA<br>Buffer kit (0.5 mM EDTA)	GBGDK-1151-buffer	4	47	19	99.06	99.76
TIAC1165<br>GenElute Bacterial gDNA<br>Buffer kit (0.5 mM EDTA)	GBGDK-1165-buffer	4	41	16	99.01	99.76
TIAC1660<br>GenElute Bacterial gDNA<br>Buffer kit (0.5 mM EDTA)	GBGDK-1660-buffer	4	31	15	98.50	97.06
TIAC1151<br>Easy-DNA gDNA<br>Purification<br>10 mM Tris-HCl (pH 8.5)	Easy-DNATIAC1151-2	1	61	20	99.75	99.76
TIAC1152<br>Easy-DNA gDNA<br>Purification<br>10 mM Tris-HCl (pH 8.5)	Easy-DNATIAC1152-2	1	61	20	99.77	99.76
TIAC1153<br>Easy-DNA gDNA<br>Purification<br>10 mM Tris-HCl (pH 8.5)	Easy-DNATIAC1153-2	1	28	11	99.69	99.48
TIAC1165<br>Easy-DNA gDNA<br>Purification<br>10 mM Tris-HCl (pH 8.5)	Easy-DNATIAC1165-2	1	66	22	99.76	99.76
TIAC1169<br>Easy-DNA gDNA<br>Purification<br>10 mM Tris-HCl (pH 8.5)	Easy-DNATIAC1169-2	1	53	19	99.77	99.72
TIAC1638<br>Easy-DNA gDNA<br>Purification<br>10 mM Tris-HCl (pH 8.5)	Easy-DNATIAC1638-2	1	55	20	99.73	98.81
TIAC1660<br>Easy-DNA gDNA<br>Purification<br>10 mM Tris-HCl (pH 8.5)	Easy-DNATIAC1660-2	1	54	20	99.78	97.06
TIAC1151<br>Easy-DNA gDNA<br>Purification<br>10 mM Tris-HCl (pH 8.5)	EasyDNATIAC-1151-2	2	69	24	99.72	99.76
TIAC1165<br>Easy-DNA gDNA<br>Purification<br>10 mM Tris-HCl (pH 8.5)	EasyDNATIAC-1165-2	2	52	18	99.71	99.76

TIAC1151<br>Easy-DNA gDNA<br>Purification<br>10 mM Tris-HCl (pH 8.5)	Easy-DNA-TIAC-<br>1151-2	3	56	20	99.68	99.76
TIAC1165<br>Easy-DNA gDNA<br>Purification<br>10 mM Tris-HCl (pH 8.5)	Easy-DNA-TIAC-<br>1165-2	3	44	16	99.69	99.76
TIAC1151<br>Genomic-tip 20/G<br>10 mM Tris-HCl (pH 8.5)	gTip20TIAC-1151-1	2	41	18	99.71	99.76
TIAC1152<br>Genomic-tip 20/G<br>10 mM Tris-HCl (pH 8.5)	gTip20TIAC-1152-1	2	34	16	99.73	99.72
TIAC1153<br>Genomic-tip 20/G<br>10 mM Tris-HCl (pH 8.5)	gTip20TIAC-1153-1	2	50	19	99.74	99.52
TIAC1165<br>Genomic-tip 20/G<br>10 mM Tris-HCl (pH 8.5)	gTip20TIAC-1165-1	2	37	17	99.72	99.76
TIAC1169<br>Genomic-tip 20/G<br>10 mM Tris-HCl (pH 8.5)	gTip20TIAC-1169-1	2	56	23	99.74	99.72
TIAC1638<br>Genomic-tip 20/G<br>10 mM Tris-HCl (pH 8.5)	gTip20TIAC-1638-1	2	37	17	99.75	98.81
TIAC1660<br>Genomic-tip 20/G<br>10 mM Tris-HCl (pH 8.5)	gTip20TIAC-1660-1	2	54	23	99.76	97.06
TIAC1151<br>Genomic-tip 20/G<br>10 mM Tris-HCl (pH 8.5)	gTip20-TIAC1151-1	1	58	28	99.73	99.76
TIAC1165<br>Genomic-tip 20/G<br>10 mM Tris-HCl (pH 8.5)	gTip20-TIAC1165-1	1	38	17	99.71	99.76
TIAC1151<br>Genomic-tip 20/G<br>10 mM Tris-HCl (pH 8.5)	gTip-20-TIAC-1151-1	3	42	20	99.70	99.76
TIAC1165<br>Genomic-tip 20/G<br>10 mM Tris-HCl (pH 8.5)	gTip-20-TIAC-1165-1	3	41	19	99.70	99.76
TIAC1151<br>MasterPure Complete DNA<br>Purification<br>10 mM Tris-HCl (pH 8.5)	MasterPureTIAC-<br>1151-TrisHCl	2	63	22	99.67	99.76
TIAC1152<br>MasterPure Complete DNA<br>Purification<br>10 mM Tris-HCl (pH 8.5)	MasterPureTIAC-<br>1152-TrisHCl	2	56	19	99.66	99.76

TIAC1153<br>MasterPure Complete DNA Purification<br>10 mM Tris-HCl (pH 8.5)	MasterPureTIAC-1153-TrisHCl	2	47	16	99.65	99.52
TIAC1165<br>MasterPure Complete DNA Purification<br>10 mM Tris-HCl (pH 8.5)	MasterPureTIAC-1165-TrisHCl	2	38	13	99.65	99.76
TIAC1169<br>MasterPure Complete DNA Purification<br>10 mM Tris-HCl (pH 8.5)	MasterPureTIAC-1169-TrisHCl	2	49	17	99.71	99.72
TIAC1638<br>MasterPure Complete DNA Purification<br>10 mM Tris-HCl (pH 8.5)	MasterPureTIAC-1638-TrisHCl	2	42	18	99.69	98.81
TIAC1660<br>MasterPure Complete DNA Purification<br>10 mM Tris-HCl (pH 8.5)	MasterPureTIAC-1660-TrisHCl	2	62	25	99.75	97.06
TIAC1151<br>MasterPure Complete DNA Purification<br>10 mM Tris-HCl (pH 8.5)	MasterPure-TIAC1151-TrisHCl	1	41	15	99.74	99.76
TIAC1165<br>MasterPure Complete DNA Purification<br>10 mM Tris-HCl (pH 8.5)	MasterPure-TIAC1165-TrisHCl	1	38	15	99.72	99.76
TIAC1151<br>MasterPure Complete DNA Purification<br>10 mM Tris-HCl (pH 8.5)	MasterPure-TIAC-1151-TrisHCl	3	40	14	99.58	99.76
TIAC1165<br>MasterPure Complete DNA Purification<br>10 mM Tris-HCl (pH 8.5)	MasterPure-TIAC-1165-TrisHCl	3	32	13	99.60	99.76
TIAC1151<br>MasterPure Complete DNA Purification<br>Buffer kit (1.0 mM EDTA)	MCDPK-1151-buffer	4	53	23	99.05	99.76
TIAC1165<br>MasterPure Complete DNA Purification<br>Buffer kit (1.0 mM EDTA)	MCDPK-1165-buffer	4	46	19	98.97	99.76
TIAC1660<br>MasterPure Complete DNA Purification<br>Buffer kit (1.0 mM EDTA)	MCDPK-1660-buffer	4	30	14	98.87	97.06
TIAC1151<br>Wizard gDNA Purification<br>10 mM Tris-HCl (pH 8.5)	Wizard-TIAC-1151-TrisHCl	3	35	14	99.68	99.76
TIAC1152<br>Wizard gDNA Purification<br>10 mM Tris-HCl (pH 8.5)	Wizard-TIAC-1152-TrisHCl	3	31	11	99.70	99.76
TIAC1153<br>Wizard gDNA Purification<br>10 mM Tris-HCl (pH 8.5)	Wizard-TIAC-1153-TrisHCl	3	46	17	99.75	99.52

TIAC1165<br>Wizard gDNA Purification<br>10 mM Tris-HCl (pH 8.5)	Wizard-TIAC-1165-<br>TrisHCl	3	29	12	99.67	99.76
TIAC1169<br>Wizard gDNA Purification<br>10 mM Tris-HCl (pH 8.5)	Wizard-TIAC-1169-<br>TrisHCl	3	41	16	99.66	99.72
TIAC1638<br>Wizard gDNA Purification<br>10 mM Tris-HCl (pH 8.5)	Wizard-TIAC-1638-<br>TrisHCl	3	39	16	99.66	98.81
TIAC1660<br>Wizard gDNA Purification<br>10 mM Tris-HCl (pH 8.5)	Wizard-TIAC-1660-<br>TrisHCl	3	33	14	99.72	97.02
TIAC1151<br>Wizard gDNA Purification<br>10 mM Tris-HCl (pH 8.5)	Wizard-TIAC1151-<br>TrisHCl	1	45	17	99.75	99.76
TIAC1165<br>Wizard gDNA Purification<br>10 mM Tris-HCl (pH 8.5)	Wizard-TIAC1165-<br>TrisHCl	1	36	14	99.72	99.76
TIAC1151<br>Wizard gDNA Purification<br>10 mM Tris-HCl (pH 8.5)	WizardTIAC-1151-<br>TrisHCl	2	47	16	99.74	99.76
TIAC1165<br>Wizard gDNA Purification<br>10 mM Tris-HCl (pH 8.5)	WizardTIAC-1165-<br>TrisHCl	2	38	15	99.73	99.76
TIAC1151<br>Wizard gDNA Purification<br>Buffer kit (0.5 mM EDTA)	WGDPK-1151-buffer	4	41	16	99.01	99.76
TIAC1165<br>Wizard gDNA Purification<br>Buffer kit (0.5 mM EDTA)	WGDPK-1165-buffer	4	43	16	98.99	99.76
TIAC1660<br>Wizard gDNA Purification<br>Buffer kit (0.5 mM EDTA)	WGDPK-1660-buffer	4	55	32	98.95	97.06
TIAC1151<br>UltraClean Microbial DNA<br>isolation<br>Buffer kit	UltraCleanTIAC1151-<br>3	1	81	27	99.78	99.76
TIAC1152<br>UltraClean Microbial DNA<br>isolation<br>Buffer kit	UltraCleanTIAC1152-<br>3	1	53	20	99.76	99.72
TIAC1153<br>UltraClean Microbial DNA<br>isolation<br>Buffer kit	UltraCleanTIAC1153-<br>3	1	53	19	99.79	99.52
TIAC1165<br>UltraClean Microbial DNA<br>isolation<br>Buffer kit	UltraCleanTIAC1165-<br>3	1	60	27	99.71	99.72

TIAC1169<br>UltraClean Microbial DNA isolation<br>Buffer kit	UltraCleanTIAC1169-3	1	100	35	99.77	99.72
TIAC1638<br>UltraClean Microbial DNA isolation<br>Buffer kit	UltraCleanTIAC1638-3	1	62	24	99.77	98.81
TIAC1660<br>UltraClean Microbial DNA isolation<br>Buffer kit	UltraCleanTIAC1660-3	1	75	30	99.74	97.06
TIAC1151<br>UltraClean Microbial DNA isolation<br>Buffer kit	UltraCleanTIAC-1151-3	2	47	17	99.73	99.76
TIAC1165<br>UltraClean Microbial DNA isolation<br>Buffer kit	UltraCleanTIAC-1165-3	2	48	17	99.74	99.76
TIAC1151<br>UltraClean Microbial DNA isolation<br>Buffer kit	UltraClean-TIAC-1151-3	3	42	18	99.72	99.76
TIAC1165<br>UltraClean Microbial DNA isolation<br>Buffer kit	UltraClean-TIAC-1165-3	3	53	20	99.73	99.76
TIAC1151<br>NucliSENS miniMag<br>Buffer kit	MiniMAG-TIAC-1151-3	3	37	14	99.61	99.76
TIAC1152<br>NucliSENS miniMag<br>Buffer kit	MiniMAG-TIAC-1152-3	3	48	17	99.41	99.76
TIAC1153<br>NucliSENS miniMag<br>Buffer kit	MiniMAG-TIAC-1153-3	3	46	17	99.64	99.52
TIAC1165<br>NucliSENS miniMag<br>Buffer kit	MiniMAG-TIAC-1165-3	3	38	14	99.58	99.76
TIAC1169<br>NucliSENS miniMag<br>Buffer kit	MiniMAG-TIAC-1169-3	3	37	14	99.52	99.72
TIAC1638<br>NucliSENS miniMag<br>Buffer kit	MiniMAG-TIAC-1638-3	3	34	15	99.51	98.81
TIAC1660<br>NucliSENS miniMag<br>Buffer kit	MiniMAG-TIAC-1660-3	3	51	21	99.58	97.06
TIAC1151<br>NucliSENS miniMag<br>Buffer kit	miniMAG-TIAC-1151-3	1	37	14	99.61	99.76

TIAC1165 NucliSENS miniMag Buffer kit	miniMAG-TIAC-1165-3	1	38	14	99.58	99.76
TIAC1151 NucliSENS miniMag Buffer kit	miniMAGTIAC-1151-3	2	58	20	99.69	99.76
TIAC1165 NucliSENS miniMag Buffer kit	miniMAGTIAC-1165-3	2	54	17	99.63	99.76

In the first column, the sample name, used kit and the applied buffer for DNA rehydration or elution is mentioned. The second column contains the identity of each library as also indicated as the name of the WGS data in NCBI SRA. The following, consecutive columns list the run name, median sequencing depth, Interquartile range (IQR) of the median sequencing depth, percentage of reads mapping back to the assembly, and percentage of cgMLST loci detected (on a total of 2,513 loci), respectively.

3.8. Supplementary Table S8: SNP matrix per kit containing outbreak isolates

Supplementary Table S8 The influence of the DNA extraction kit on the pairwise SNP differences between different outbreak isolates

**DNeasy Blood & Tissue kit**	TIAC1151	TIAC1152	TIAC1165	TIAC1169
TIAC1151	0			
TIAC1152	0	0		
TIAC1165	8	8	0	
TIAC1169	1	7	9	0
Median	7.5			
IQR	5.5			
**DNeasy UltraClean Microbial kit**	TIAC1151	TIAC1152	TIAC1165	TIAC1169
TIAC1151	0			
TIAC1152	0	0		
TIAC1165	13	13	0	
TIAC1169	1	1	14	0
Median	7			
IQR	12			
**Easy-DNA gDNA Purification kit**	TIAC1151	TIAC1152	TIAC1165	TIAC1169
TIAC1151	0			
TIAC1152	0	0		
TIAC1165	5	5	0	
TIAC1169	1	1	6	0
Median	3			
IQR	4			
**GenElute Bacterial gDNA kit**	TIAC1151	TIAC1152	TIAC1165	TIAC1169
TIAC1151	0			
TIAC1152	0	0		
TIAC1165	3	3	0	
TIAC1169	1	1	4	0
Median	2			
IQR	2			

**Genomic-tip 20/G**	TIAC1151	TIAC1152	TIAC1165	TIAC1169
TIAC1151	0			
TIAC1152	0	0		
TIAC1165	8	8	0	
TIAC1169	1	1	9	0
Median	4.5			
IQR	7			
**MasterPure Complete DNA Purification kit**	TIAC1151	TIAC1152	TIAC1165	TIAC1169
TIAC1151	0			
TIAC1152	0	0		
TIAC1165	3	3	0	
TIAC1169	1	1	4	0
Median	2			
IQR	2			
**NucliSENS miniMag**	TIAC1151	TIAC1152	TIAC1165	TIAC1169
TIAC1151	0			
TIAC1152	1	0		
TIAC1165	8	9	0	
TIAC1169	1	3	9	0
Median	5.5			
IQR	7.3			
**Wizard gDNA Purification kit**	TIAC1151	TIAC1152	TIAC1165	TIAC1169
TIAC1151	0			
TIAC1152	0	0		
TIAC1165	2	2	0	
TIAC1169	1	1	3	0
Median	1.5			
IQR	1			

The SNP matrix demonstrates the impact of the kit on the number of SNP discrepancies compared to the Sakai E. coli *O157:H7 reference genome between outbreak isolates. For each kit, the median number of SNPs different between the outbreak isolates, and the interquartile range, as calculated based on pairwise SNP differences between individual samples in shared high-quality positions.*

3.9. Supplementary Table S9: Location of discrepant SNPs

Supplementary Table S9 Overview of the discrepant SNP compared to the reference genome between outbreak isolates.

Kit	Isolate	Sample/Library name	Position														
			ydcC													-	*pgpC*
			274109	274121	274130	274273	274293	274307	274308	274316	274319	274321	274325	274334	274367	1590037	3418925
DNeasy Blood & Tissue	TIAC1151	Blood-TissueTIAC1151-TrisHCl	-	-	-	-	-	-	-	-	-	-	-	-	-	-	-
	TIAC1152	Blood-TissueTIAC1152-TrisHCl	-	-	-	-	-	-	-	-	-	-	-	-	-	-	-
	TIAC1165	Blood-TissueTIAC1165-TrisHCl	-	-	-	-	-	G	C	A	A	A	G	G	A	-	-
	TIAC1169	Blood-TissueTIAC1169-TrisHCl	-	-	-	-	-	-	-	-	-	-	-	-	-	-	G
DNeasy UltraClean Microbial	TIAC1151	UltraCleanTIAC1151-3	-	-	-	-	-	-	-	-	-	-	-	-	-	-	-
	TIAC1152	UltraCleanTIAC1152-3	-	-	-	-	-	-	-	-	-	-	-	-	-	-	-
	TIAC1165	UltraCleanTIAC1165-3	C	C	A	G	C	G	C	A	A	A	G	G	A	-	-
	TIAC1169	UltraCleanTIAC1169-3	-	-	-	-	-	-	-	-	-	-	-	-	-	-	G
Easy-DNA gDNA Purification	TIAC1151	Easy-DNATIAC1151-2	-	-	-	-	-	-	-	-	-	-	-	-	-	-	-
	TIAC1152	Easy-DNATIAC1152-2	-	-	-	-	-	-	-	-	-	-	-	-	-	-	-
	TIAC1165	Easy-DNATIAC1165-2	C	C	-	-	-	-	-	-	-	-	G	G	A	-	-
	TIAC1169	Easy-DNATIAC1169-2	-	-	-	-	-	-	-	-	-	-	-	-	-	-	G
GenElute Bacterial gDNA	TIAC1151	GenEluteTIAC-1151-TrisHCl	-	-	-	-	-	-	-	-	-	-	-	-	-	-	-
	TIAC1152	GenEluteTIAC-1152-TrisHCl	-	-	-	-	-	-	-	-	-	-	-	-	-	-	-
	TIAC1165	GenEluteTIAC-1165-TrisHCl	-	-	-	-	-	-	-	-	-	-	G	G	A	-	-
	TIAC1169	GenEluteTIAC-1169-TrisHCl	-	-	-	-	-	-	-	-	-	-	-	-	-	-	G
Genomic-tip 20/G	TIAC1151	gTip20TIAC-1151-1	-	-	-	-	-	-	-	-	-	-	-	-	-	-	-
	TIAC1152	gTip20TIAC-1152-1	-	-	-	-	-	-	-	-	-	-	-	-	-	-	-
	TIAC1165	gTip20TIAC-1165-1	-	-	-	-	-	G	C	A	A	A	G	G	A	-	-
	TIAC1169	gTip20TIAC-1169-1	-	-	-	-	-	-	-	-	-	-	-	-	-	-	G
MasterPure Complete DNA Purifcation	TIAC1151	MasterPureTIAC-1151-TrisHCl	-	-	-	-	-	-	-	-	-	-	-	-	-	-	-
	TIAC1152	MasterPureTIAC-1152-TrisHCl	-	-	-	-	-	-	-	-	-	-	-	-	-	-	-
	TIAC1165	MasterPureTIAC-1165-TrisHCl	-	-	-	-	-	-	-	-	-	-	G	G	A	-	-

	TIAC1169	MasterPureTIAC-1169-TrisHCl	-	-	-	-	-	-	-	-	-	-	-	-	-	-	G
NucliSENS miniMag	TIAC1151	MiniMAG-TIAC-1151-3	-	-	-	-	-	-	-	-	-	-	-	-	-	-	-
	TIAC1152	MiniMAG-TIAC-1152-3	-	-	-	-	-	-	-	-	-	-	-	-	-	T	-
	TIAC1165	MiniMAG-TIAC-1165-3	-	-	-	-	-	G	C	A	A	A	G	G	A	-	-
	TIAC1169	MiniMAG-TIAC-1169-3	-	-	-	-	-	-	-	-	-	-	-	-	-	-	G
Wizard gDNA Purification	TIAC1151	Wizard-TIAC-1151-TrisHCl	-	-	-	-	-	-	-	-	-	-	-	-	-	-	-
	TIAC1152	Wizard-TIAC-1152-TrisHCl	-	-	-	-	-	-	-	-	-	-	-	-	-	-	-
	TIAC1165	Wizard-TIAC-1165-TrisHCl	-	-	-	-	-	-	-	-	-	-	-	G	A	-	-
	TIAC1169	Wizard-TIAC-1169-TrisHCl	-	-	-	-	-	-	-	-	-	-	-	-	-	-	G

Only positions for which a discrepancy existed between the different outbreak samples are listed (i.e. SNPs compared to the Sakai E. coli *O157:H7 reference genome that had the same base in all samples are not listed), as calculated using comparison of all samples combined. A dash ('-') indicates that the reference genome position was found for the sample in question at that genomic position, whereas a position indicated in orange indicates that a SNP was found for the sample in question at that genomic positions. Positions correspond to the genomic coordinate in the reference genome; positions in coding regions are annotated with the corresponding gene name.*

3.10. Supplementary Table S10: Influence of EDTA on SNP number between outbreak isolates per kit

Supplementary Table S10 The influence of EDTA on the number of SNPs different between different outbreak samples per kit

DNeasy Blood & Tissue kit (0.5mM EDTA compared to 10 mM Tris-HCl (pH 8.5))				
Isolate_Buffer	TIAC1151_10 mM Tris-HCl	TIAC1152_10 mM Tris-HCl	TIAC1165_10 mM Tris-HCl	TIAC1169_10 mM Tris-HCl
TIAC1151_0.5 mM EDTA	0	0	8	1
TIAC1165_0.5 mM EDTA	10	11	0	11
Median	4.5			
IQR	10.25			
**DNeasy Blood & Tissue kit (EDTA removed compared to 10 mM Tris-HCl (pH 8.5))**				
Isolate_Buffer	TIAC1151_10 mM Tris-HCl	TIAC1152_10 mM Tris-HCl	TIAC1165_10 mM Tris-HCl	TIAC1169_10 mM Tris-HCl
TIAC1151_EDTA removed	0	0	8	1
TIAC1165_EDTA removed	9	9	0	10
Median	4.5			
IQR	9			
**GenElute Bacterial gDNA kit (0.5 mM EDTA compared to 10 mM Tris-HCl (pH 8.5))**				
Isolate_Buffer	TIAC1151_10 mM Tris-HCl	TIAC1152_10 mM Tris-HCl	TIAC1165_10 mM Tris-HCl	TIAC1169_10 mM Tris-HCl
TIAC1151_0.5 mM EDTA	0	0	3	1
TIAC1165_0.5 mM EDTA	8	8	0	9
Median	2			
IQR	8			
**MasterPure Complete DNA Purification kit (1.0 mM EDTA compared to 10 mM Tris-HCl (pH 8.5))**				
Isolate_Buffer	TIAC1151_10 mM Tris-HCl	TIAC1152_10 mM Tris-HCl	TIAC1165_10 mM Tris-HCl	TIAC1169_10 mM Tris-HCl
TIAC1151_1.0 mM EDTA	0	0	3	1
TIAC1165_1.0 mM EDTA	10	10	0	11
Median	2			
IQR	10			
**Wizard gDNA Purification kit (1.0 mM EDTA compared to 10 mM Tris-HCl (pH 8.5))**				
Isolate_Buffer	TIAC1151_10 mM Tris-HCl	TIAC1152_10 mM Tris-HCl	TIAC1165_10 mM Tris-HCl	TIAC1169_10 mM Tris-HCl
TIAC1151_1.0 mM EDTA	0	0	2	1
TIAC1165_1.0 mM EDTA	9	9	0	10
Median	1.5			
IQR	9			

The SNP matrix demonstrates the impact of EDTA on the number of SNPs different compared to the Sakai E. coli *O157:H7 reference genome between outbreak samples. For each kit, the median number of SNPs different between the outbreak samples from which the DNA was eluted/rehydrated in 10 mM Tris-HCl (pH 8.5) and the two outbreak samples (TIAC1151 and TIAC1165) from which the DNA was eluted/rehydrated in the accompanying kit buffer containing 0.5 mM (DNeasy Blood & Tissue and GenElute Bacterial gDNA kits) or 1.0 mM (MasterPure Complete DNA Purification and Wizard gDNA Purification kits) EDTA are shown, together with the interquartile range. For the DNeasy Blood & Tissue kit, the median number of SNPs different between the outbreak samples from which the DNA was eluted/rehydrated in 10 mM Tris-HCl (pH 8.5) and the two outbreak samples (TIAC1151 and TIAC1165) from which EDTA removal was performed using the DNA Clean & Concentrator kit after DNA elution in the accompanying kit buffer containing 0.5 mM EDTA is also shown together with the interquartile range (IQR).*

3.11. Supplementary Table S11: Accession numbers of datasets in NCBI SRA

Supplementary Table S11 Accession numbers of the datasets deposited in NCBI SRA

Accession	Biosample Accession	Sample Name	Library ID	Filename 1	Filename 2
SRR10201494	SAMN12871462	TIAC1151	Easy-DNATIAC1151-2	Easy-DNATIAC1151-2_S15_L001_R1_001.fastq.gz	Easy-DNATIAC1151-2_S15_L001_R2_001.fastq.gz
SRR10201493	SAMN12871462	TIAC1151	EasyDNATIAC-1151-2	EasyDNATIAC-1151-2_S28_L001_R1_001.fastq.gz	EasyDNATIAC-1151-2_S28_L001_R2_001.fastq.gz
SRR10201479	SAMN12871462	TIAC1151	Blood-Tissue-TIAC-1151-TrisHCl	Blood-Tissue-TIAC-1151-TrisHCl_S21_L001_R1_001.fastq.gz	Blood-Tissue-TIAC-1151-TrisHCl_S21_L001_R2_001.fastq.gz
SRR10201468	SAMN12871462	TIAC1151	Wizard-TIAC1151-TrisHCl	Wizard-TIAC1151-TrisHCl_S24_L001_R1_001.fastq.gz	Wizard-TIAC1151-TrisHCl_S24_L001_R2_001.fastq.gz
SRR10201457	SAMN12871462	TIAC1151	UltraCleanTIAC1151-3	UltraCleanTIAC1151-3_S8_L001_R1_001.fastq.gz	UltraCleanTIAC1151-3_S8_L001_R2_001.fastq.gz
SRR10201446	SAMN12871462	TIAC1151	DBTK-1151-buffer	DBTK-1151-buffer_S8_L001_R1_001.fastq.gz	DBTK-1151-buffer_S8_L001_R2_001.fastq.gz
SRR10201435	SAMN12871462	TIAC1151	Blood-TissueTIAC-1151-TrisHCl	Blood-TissueTIAC-1151-TrisHCl_S26_L001_R1_001.fastq.gz	Blood-TissueTIAC-1151-TrisHCl_S26_L001_R2_001.fastq.gz
SRR10201424	SAMN12871462	TIAC1151	gTip20TIAC-1151-1	gTip20TIAC-1151-1_S8_L001_R1_001.fastq.gz	gTip20TIAC-1151-1_S8_L001_R2_001.fastq.gz
SRR10201413	SAMN12871462	TIAC1151	UltraClean-TIAC-1151-3	UltraClean-TIAC-1151-3_S25_L001_R1_001.fastq.gz	UltraClean-TIAC-1151-3_S25_L001_R2_001.fastq.gz
SRR10201402	SAMN12871462	TIAC1151	GenElute-TIAC1151-TrisHCl	GenElute-TIAC1151-TrisHCl_S26_L001_R1_001.fastq.gz	GenElute-TIAC1151-TrisHCl_S26_L001_R2_001.fastq.gz
SRR10201492	SAMN12871462	TIAC1151	MCDPK-1151-buffer	MCDPK-1151-buffer_S15_L001_R1_001.fastq.gz	MCDPK-1151-buffer_S15_L001_R2_001.fastq.gz
SRR10201488	SAMN12871462	TIAC1151	Easy-DNA-TIAC-1151-2	Easy-DNA-TIAC-1151-2_S15_L001_R1_001.fastq.gz	Easy-DNA-TIAC-1151-2_S15_L001_R2_001.fastq.gz
SRR10201487	SAMN12871462	TIAC1151	miniMAG-TIAC-1151-3	miniMAG-TIAC-1151-3_S30_L001_R1_001.fastq.gz	miniMAG-TIAC-1151-3_S30_L001_R2_001.fastq.gz
SRR10201486	SAMN12871462	TIAC1151	DBTK-1151-cleanup	DBTK-1151-cleanup_S12_L001_R1_001.fastq.gz	DBTK-1151-cleanup_S12_L001_R2_001.fastq.gz
SRR10201485	SAMN12871462	TIAC1151	MiniMAG-TIAC-1151-3	MiniMAG-TIAC-1151-3_S8_L001_R1_001.fastq.gz	MiniMAG-TIAC-1151-3_S8_L001_R2_001.fastq.gz
SRR10201484	SAMN12871462	TIAC1151	Blood-TissueTIAC1151-TrisHCl	Blood-TissueTIAC1151-TrisHCl_S1_L001_R1_001.fastq.gz	Blood-TissueTIAC1151-TrisHCl_S1_L001_R2_001.fastq.gz
SRR10201483	SAMN12871462	TIAC1151	GenEluteTIAC-1151-TrisHCl	GenEluteTIAC-1151-TrisHCl_S1_L001_R1_001.fastq.gz	GenEluteTIAC-1151-TrisHCl_S1_L001_R2_001.fastq.gz
SRR10201482	SAMN12871462	TIAC1151	WGDPK-1151-buffer	WGDPK-1151-buffer_S2_L001_R1_001.fastq.gz	WGDPK-1151-buffer_S2_L001_R2_001.fastq.gz
SRR10201481	SAMN12871462	TIAC1151	UltraCleanTIAC-1151-3	UltraCleanTIAC-1151-3_S22_L001_R1_001.fastq.gz	UltraCleanTIAC-1151-3_S22_L001_R2_001.fastq.gz

SRR10201480	SAMN12871462	TIAC1151	MasterPureTIAC-1151-TrisHCl	MasterPureTIAC-1151-TrisHCl_S15_L001_R1_001.fastq.gz	MasterPureTIAC-1151-TrisHCl_S15_L001_R2_001.fastq.gz
SRR10201478	SAMN12871462	TIAC1151	WizardTIAC-1151-TrisHCl	WizardTIAC-1151-TrisHCl_S24_L001_R1_001.fastq.gz	WizardTIAC-1151-TrisHCl_S24_L001_R2_001.fastq.gz
SRR10201477	SAMN12871462	TIAC1151	GBGDK-1151-buffer	GBGDK-1151-buffer_S5_L001_R1_001.fastq.gz	GBGDK-1151-buffer_S5_L001_R2_001.fastq.gz
SRR10201476	SAMN12871462	TIAC1151	GenElute-TIAC-1151-TrisHCl	GenElute-TIAC-1151-TrisHCl_S19_L001_R1_001.fastq.gz	GenElute-TIAC-1151-TrisHCl_S19_L001_R2_001.fastq.gz
SRR10201475	SAMN12871462	TIAC1151	gTip-20-TIAC-1151-1	gTip-20-TIAC-1151-1_S17_L001_R1_001.fastq.gz	gTip-20-TIAC-1151-1_S17_L001_R2_001.fastq.gz
SRR10201474	SAMN12871462	TIAC1151	MasterPure-TIAC1151-TrisHCl	MasterPure-TIAC1151-TrisHCl_S22_L001_R1_001.fastq.gz	MasterPure-TIAC1151-TrisHCl_S22_L001_R2_001.fastq.gz
SRR10201473	SAMN12871462	TIAC1151	miniMAGTIAC-1151-3	miniMAGTIAC-1151-3_S30_L001_R1_001.fastq.gz	miniMAGTIAC-1151-3_S30_L001_R2_001.fastq.gz
SRR10201472	SAMN12871462	TIAC1151	MasterPure-TIAC-1151-TrisHCl	MasterPure-TIAC-1151-TrisHCl_S23_L001_R1_001.fastq.gz	MasterPure-TIAC-1151-TrisHCl_S23_L001_R2_001.fastq.gz
SRR10201471	SAMN12871462	TIAC1151	Wizard-TIAC-1151-TrisHCl	Wizard-TIAC-1151-TrisHCl_S1_L001_R1_001.fastq.gz	Wizard-TIAC-1151-TrisHCl_S1_L001_R2_001.fastq.gz
SRR10201470	SAMN12871462	TIAC1151	gTip20-TIAC1151-1	gTip20-TIAC1151-1_S28_L001_R1_001.fastq.gz	gTip20-TIAC1151-1_S28_L001_R2_001.fastq.gz
SRR10201469	SAMN12871463	TIAC1152	Wizard-TIAC-1152-TrisHCl	Wizard-TIAC-1152-TrisHCl_S2_L001_R1_001.fastq.gz	Wizard-TIAC-1152-TrisHCl_S2_L001_R2_001.fastq.gz
SRR10201467	SAMN12871463	TIAC1152	Easy-DNATIAC1152-2	Easy-DNATIAC1152-2_S16_L001_R1_001.fastq.gz	Easy-DNATIAC1152-2_S16_L001_R2_001.fastq.gz
SRR10201466	SAMN12871463	TIAC1152	MiniMAG-TIAC-1152-3	MiniMAG-TIAC-1152-3_S9_L001_R1_001.fastq.gz	MiniMAG-TIAC-1152-3_S9_L001_R2_001.fastq.gz
SRR10201465	SAMN12871463	TIAC1152	GenEluteTIAC-1152-TrisHCl	GenEluteTIAC-1152-TrisHCl_S2_L001_R1_001.fastq.gz	GenEluteTIAC-1152-TrisHCl_S2_L001_R2_001.fastq.gz
SRR10201464	SAMN12871463	TIAC1152	gTip20TIAC-1152-1	gTip20TIAC-1152-1_S9_L001_R1_001.fastq.gz	gTip20TIAC-1152-1_S9_L001_R2_001.fastq.gz
SRR10201463	SAMN12871463	TIAC1152	Blood-TissueTIAC1152-TrisHCl	Blood-TissueTIAC1152-TrisHCl_S2_L001_R1_001.fastq.gz	Blood-TissueTIAC1152-TrisHCl_S2_L001_R2_001.fastq.gz
SRR10201462	SAMN12871463	TIAC1152	MasterPureTIAC-1152-TrisHCl	MasterPureTIAC-1152-TrisHCl_S16_L001_R1_001.fastq.gz	MasterPureTIAC-1152-TrisHCl_S16_L001_R2_001.fastq.gz
SRR10201461	SAMN12871463	TIAC1152	UltraCleanTIAC1152-3	UltraCleanTIAC1152-3_S9_L001_R1_001.fastq.gz	UltraCleanTIAC1152-3_S9_L001_R2_001.fastq.gz
SRR10201460	SAMN12871464	TIAC1153	Easy-DNATIAC1153-2	Easy-DNATIAC1153-2_S17_L001_R1_001.fastq.gz	Easy-DNATIAC1153-2_S17_L001_R2_001.fastq.gz
SRR10201459	SAMN12871464	TIAC1153	UltraCleanTIAC1153-3	UltraCleanTIAC1153-3_S10_L001_R1_001.fastq.gz	UltraCleanTIAC1153-3_S10_L001_R2_001.fastq.gz
SRR10201458	SAMN12871464	TIAC1153	MasterPureTIAC-1153-TrisHCl	MasterPureTIAC-1153-TrisHCl_S17_L001_R1_001.fastq.gz	MasterPureTIAC-1153-TrisHCl_S17_L001_R2_001.fastq.gz
SRR10201456	SAMN12871464	TIAC1153	Wizard-TIAC-1153-TrisHCl	Wizard-TIAC-1153-TrisHCl_S3_L001_R1_001.fastq.gz	Wizard-TIAC-1153-TrisHCl_S3_L001_R2_001.fastq.gz

SRR10201455	SAMN12871464	TIAC1153	gTip20TIAC-1153-1	gTip20TIAC-1153-1_S10_L001_R1_001.fastq.gz	gTip20TIAC-1153-1_S10_L001_R2_001.fastq.gz
SRR10201454	SAMN12871464	TIAC1153	MiniMAG-TIAC-1153-3	MiniMAG-TIAC-1153-3_S10_L001_R1_001.fastq.gz	MiniMAG-TIAC-1153-3_S10_L001_R2_001.fastq.gz
SRR10201453	SAMN12871464	TIAC1153	Blood-TissueTIAC1153-TrisHCl	Blood-TissueTIAC1153-TrisHCl_S3_L001_R1_001.fastq.gz	Blood-TissueTIAC1153-TrisHCl_S3_L001_R2_001.fastq.gz
SRR10201452	SAMN12871464	TIAC1153	GenEluteTIAC-1153-TrisHCl	GenEluteTIAC-1153-TrisHCl_S3_L001_R1_001.fastq.gz	GenEluteTIAC-1153-TrisHCl_S3_L001_R2_001.fastq.gz
SRR10201451	SAMN12871465	TIAC1165	UltraCleanTIAC1165-3	UltraCleanTIAC1165-3_S11_L001_R1_001.fastq.gz	UltraCleanTIAC1165-3_S11_L001_R2_001.fastq.gz
SRR10201450	SAMN12871465	TIAC1165	MasterPure-TIAC-1165-TrisHCl	MasterPure-TIAC-1165-TrisHCl_S24_L001_R1_001.fastq.gz	MasterPure-TIAC-1165-TrisHCl_S24_L001_R2_001.fastq.gz
SRR10201449	SAMN12871465	TIAC1165	EasyDNATIAC-1165-2	EasyDNATIAC-1165-2_S29_L001_R1_001.fastq.gz	EasyDNATIAC-1165-2_S29_L001_R2_001.fastq.gz
SRR10201448	SAMN12871465	TIAC1165	miniMAGTIAC-1165-3	miniMAGTIAC-1165-3_S31_L001_R1_001.fastq.gz	miniMAGTIAC-1165-3_S31_L001_R2_001.fastq.gz
SRR10201447	SAMN12871465	TIAC1165	GenElute-TIAC-1165-TrisHCl	GenElute-TIAC-1165-TrisHCl_S20_L001_R1_001.fastq.gz	GenElute-TIAC-1165-TrisHCl_S20_L001_R2_001.fastq.gz
SRR10201445	SAMN12871465	TIAC1165	Blood-Tissue-TIAC-1165-TrisHCl	Blood-Tissue-TIAC-1165-TrisHCl_S22_L001_R1_001.fastq.gz	Blood-Tissue-TIAC-1165-TrisHCl_S22_L001_R2_001.fastq.gz
SRR10201444	SAMN12871465	TIAC1165	gTip20-TIAC1165-1	gTip20-TIAC1165-1_S29_L001_R1_001.fastq.gz	gTip20-TIAC1165-1_S29_L001_R2_001.fastq.gz
SRR10201443	SAMN12871465	TIAC1165	MasterPure-TIAC1165-TrisHCl	MasterPure-TIAC1165-TrisHCl_S23_L001_R1_001.fastq.gz	MasterPure-TIAC1165-TrisHCl_S23_L001_R2_001.fastq.gz
SRR10201442	SAMN12871465	TIAC1165	WGDPK-1165-buffer	WGDPK-1165-buffer_S3_L001_R1_001.fastq.gz	WGDPK-1165-buffer_S3_L001_R2_001.fastq.gz
SRR10201441	SAMN12871465	TIAC1165	WizardTIAC-1165-TrisHCl	WizardTIAC-1165-TrisHCl_S25_L001_R1_001.fastq.gz	WizardTIAC-1165-TrisHCl_S25_L001_R2_001.fastq.gz
SRR10201440	SAMN12871465	TIAC1165	miniMAG-TIAC-1165-3	miniMAG-TIAC-1165-3_S31_L001_R1_001.fastq.gz	miniMAG-TIAC-1165-3_S31_L001_R2_001.fastq.gz
SRR10201439	SAMN12871465	TIAC1165	Blood-TissueTIAC-1165-TrisHCl	Blood-TissueTIAC-1165-TrisHCl_S27_L001_R1_001.fastq.gz	Blood-TissueTIAC-1165-TrisHCl_S27_L001_R2_001.fastq.gz
SRR10201438	SAMN12871465	TIAC1165	gTip20TIAC-1165-1	gTip20TIAC-1165-1_S11_L001_R1_001.fastq.gz	gTip20TIAC-1165-1_S11_L001_R2_001.fastq.gz
SRR10201437	SAMN12871465	TIAC1165	Wizard-TIAC-1165-TrisHCl	Wizard-TIAC-1165-TrisHCl_S4_L001_R1_001.fastq.gz	Wizard-TIAC-1165-TrisHCl_S4_L001_R2_001.fastq.gz
SRR10201436	SAMN12871465	TIAC1165	UltraClean-TIAC-1165-3	UltraClean-TIAC-1165-3_S26_L001_R1_001.fastq.gz	UltraClean-TIAC-1165-3_S26_L001_R2_001.fastq.gz
SRR10201434	SAMN12871465	TIAC1165	MCDPK-1165-buffer	MCDPK-1165-buffer_S16_L001_R1_001.fastq.gz	MCDPK-1165-buffer_S16_L001_R2_001.fastq.gz
SRR10201433	SAMN12871465	TIAC1165	GenElute-TIAC1165-TrisHCl	GenElute-TIAC1165-TrisHCl_S27_L001_R1_001.fastq.gz	GenElute-TIAC1165-TrisHCl_S27_L001_R2_001.fastq.gz
SRR10201432	SAMN12871465	TIAC1165	UltraCleanTIAC-1165-3	UltraCleanTIAC-1165-3_S23_L001_R1_001.fastq.gz	UltraCleanTIAC-1165-3_S23_L001_R2_001.fastq.gz

SRR10201431	SAMN12871465	TIAC1165	MasterPureTIAC-1165-TrisHCl	MasterPureTIAC-1165-TrisHCl_S18_L001_R1_001.fastq.gz	MasterPureTIAC-1165-TrisHCl_S18_L001_R2_001.fastq.gz
SRR10201430	SAMN12871465	TIAC1165	GBGDK-1165-buffer	GBGDK-1165-buffer_S6_L001_R1_001.fastq.gz	GBGDK-1165-buffer_S6_L001_R2_001.fastq.gz
SRR10201429	SAMN12871465	TIAC1165	Easy-DNATIAC1165-2	Easy-DNATIAC1165-2_S18_L001_R1_001.fastq.gz	Easy-DNATIAC1165-2_S18_L001_R2_001.fastq.gz
SRR10201428	SAMN12871465	TIAC1165	DBTK-1165-cleanup	DBTK-1165-cleanup_S13_L001_R1_001.fastq.gz	DBTK-1165-cleanup_S13_L001_R2_001.fastq.gz
SRR10201427	SAMN12871465	TIAC1165	GenEluteTIAC-1165-TrisHCl	GenEluteTIAC-1165-TrisHCl_S4_L001_R1_001.fastq.gz	GenEluteTIAC-1165-TrisHCl_S4_L001_R2_001.fastq.gz
SRR10201426	SAMN12871465	TIAC1165	DBTK-1165-buffer	DBTK-1165-buffer_S9_L001_R1_001.fastq.gz	DBTK-1165-buffer_S9_L001_R2_001.fastq.gz
SRR10201425	SAMN12871465	TIAC1165	gTip-20-TIAC-1165-1	gTip-20-TIAC-1165-1_S18_L001_R1_001.fastq.gz	gTip-20-TIAC-1165-1_S18_L001_R2_001.fastq.gz
SRR10201423	SAMN12871465	TIAC1165	Easy-DNA-TIAC-1165-2	Easy-DNA-TIAC-1165-2_S16_L001_R1_001.fastq.gz	Easy-DNA-TIAC-1165-2_S16_L001_R2_001.fastq.gz
SRR10201422	SAMN12871465	TIAC1165	Blood-TissueTIAC1165-TrisHCl	Blood-TissueTIAC1165-TrisHCl_S4_L001_R1_001.fastq.gz	Blood-TissueTIAC1165-TrisHCl_S4_L001_R2_001.fastq.gz
SRR10201421	SAMN12871465	TIAC1165	Wizard-TIAC1165-TrisHCl	Wizard-TIAC1165-TrisHCl_S25_L001_R1_001.fastq.gz	Wizard-TIAC1165-TrisHCl_S25_L001_R2_001.fastq.gz
SRR10201420	SAMN12871465	TIAC1165	MiniMAG-TIAC-1165-3	MiniMAG-TIAC-1165-3_S11_L001_R1_001.fastq.gz	MiniMAG-TIAC-1165-3_S11_L001_R2_001.fastq.gz
SRR10201419	SAMN12871466	TIAC1169	MasterPureTIAC-1169-TrisHCl	MasterPureTIAC-1169-TrisHCl_S19_L001_R1_001.fastq.gz	MasterPureTIAC-1169-TrisHCl_S19_L001_R2_001.fastq.gz
SRR10201418	SAMN12871466	TIAC1169	Blood-TissueTIAC1169-TrisHCl	Blood-TissueTIAC1169-TrisHCl_S5_L001_R1_001.fastq.gz	Blood-TissueTIAC1169-TrisHCl_S5_L001_R2_001.fastq.gz
SRR10201417	SAMN12871466	TIAC1169	Easy-DNATIAC1169-2	Easy-DNATIAC1169-2_S19_L001_R1_001.fastq.gz	Easy-DNATIAC1169-2_S19_L001_R2_001.fastq.gz
SRR10201416	SAMN12871466	TIAC1169	GenEluteTIAC-1169-TrisHCl	GenEluteTIAC-1169-TrisHCl_S5_L001_R1_001.fastq.gz	GenEluteTIAC-1169-TrisHCl_S5_L001_R2_001.fastq.gz
SRR10201415	SAMN12871466	TIAC1169	Wizard-TIAC-1169-TrisHCl	Wizard-TIAC-1169-TrisHCl_S5_L001_R1_001.fastq.gz	Wizard-TIAC-1169-TrisHCl_S5_L001_R2_001.fastq.gz
SRR10201414	SAMN12871466	TIAC1169	MiniMAG-TIAC-1169-3	MiniMAG-TIAC-1169-3_S12_L001_R1_001.fastq.gz	MiniMAG-TIAC-1169-3_S12_L001_R2_001.fastq.gz
SRR10201412	SAMN12871466	TIAC1169	gTip20TIAC-1169-1	gTip20TIAC-1169-1_S12_L001_R1_001.fastq.gz	gTip20TIAC-1169-1_S12_L001_R2_001.fastq.gz
SRR10201411	SAMN12871466	TIAC1169	UltraCleanTIAC1169-3	UltraCleanTIAC1169-3_S12_L001_R1_001.fastq.gz	UltraCleanTIAC1169-3_S12_L001_R2_001.fastq.gz
SRR10201410	SAMN12871467	TIAC1638	UltraCleanTIAC1638-3	UltraCleanTIAC1638-3_S13_L001_R1_001.fastq.gz	UltraCleanTIAC1638-3_S13_L001_R2_001.fastq.gz
SRR10201409	SAMN12871467	TIAC1638	Easy-DNATIAC1638-2	Easy-DNATIAC1638-2_S20_L001_R1_001.fastq.gz	Easy-DNATIAC1638-2_S20_L001_R2_001.fastq.gz

SRR10201408	SAMN12871467	TIAC1638	GenEluteTIAC-1638-TrisHCl	GenEluteTIAC-1638-TrisHCl_S6_L001_R1_001.fastq.gz	GenEluteTIAC-1638-TrisHCl_S6_L001_R2_001.fastq.gz
SRR10201407	SAMN12871467	TIAC1638	Blood-TissueTIAC1638-TrisHCl	Blood-TissueTIAC1638-TrisHCl_S6_L001_R1_001.fastq.gz	Blood-TissueTIAC1638-TrisHCl_S6_L001_R2_001.fastq.gz
SRR10201406	SAMN12871467	TIAC1638	MiniMAG-TIAC-1638-3	MiniMAG-TIAC-1638-3_S13_L001_R1_001.fastq.gz	MiniMAG-TIAC-1638-3_S13_L001_R2_001.fastq.gz
SRR10201405	SAMN12871467	TIAC1638	gTip20TIAC-1638-1	gTip20TIAC-1638-1_S13_L001_R1_001.fastq.gz	gTip20TIAC-1638-1_S13_L001_R2_001.fastq.gz
SRR10201404	SAMN12871467	TIAC1638	Wizard-TIAC-1638-TrisHCl	Wizard-TIAC-1638-TrisHCl_S6_L001_R1_001.fastq.gz	Wizard-TIAC-1638-TrisHCl_S6_L001_R2_001.fastq.gz
SRR10201403	SAMN12871467	TIAC1638	MasterPureTIAC-1638-TrisHCl	MasterPureTIAC-1638-TrisHCl_S20_L001_R1_001.fastq.gz	MasterPureTIAC-1638-TrisHCl_S20_L001_R2_001.fastq.gz
SRR10201401	SAMN12871468	TIAC1660	GBGDK-1660-buffer	GBGDK-1660-buffer_S7_L001_R1_001.fastq.gz	GBGDK-1660-buffer_S7_L001_R2_001.fastq.gz
SRR10201400	SAMN12871468	TIAC1660	DBTK-1660-cleanup	DBTK-1660-cleanup_S14_L001_R1_001.fastq.gz	DBTK-1660-cleanup_S14_L001_R2_001.fastq.gz
SRR10201399	SAMN12871468	TIAC1660	UltraCleanTIAC1660-3	UltraCleanTIAC1660-3_S14_L001_R1_001.fastq.gz	UltraCleanTIAC1660-3_S14_L001_R2_001.fastq.gz
SRR10201398	SAMN12871468	TIAC1660	GenEluteTIAC-1660-TrisHCl	GenEluteTIAC-1660-TrisHCl_S7_L001_R1_001.fastq.gz	GenEluteTIAC-1660-TrisHCl_S7_L001_R2_001.fastq.gz
SRR10201397	SAMN12871468	TIAC1660	Wizard-TIAC-1660-TrisHCl	Wizard-TIAC-1660-TrisHCl_S7_L001_R1_001.fastq.gz	Wizard-TIAC-1660-TrisHCl_S7_L001_R2_001.fastq.gz
SRR10201396	SAMN12871468	TIAC1660	MasterPureTIAC-1660-TrisHCl	MasterPureTIAC-1660-TrisHCl_S21_L001_R1_001.fastq.gz	MasterPureTIAC-1660-TrisHCl_S21_L001_R2_001.fastq.gz
SRR10201395	SAMN12871468	TIAC1660	Easy-DNATIAC1660-2	Easy-DNATIAC1660-2_S21_L001_R1_001.fastq.gz	Easy-DNATIAC1660-2_S21_L001_R2_001.fastq.gz
SRR10201394	SAMN12871468	TIAC1660	DBTK-1660-buffer	DBTK-1660-buffer_S10_L001_R1_001.fastq.gz	DBTK-1660-buffer_S10_L001_R2_001.fastq.gz
SRR10201393	SAMN12871468	TIAC1660	gTip20TIAC-1660-1	gTip20TIAC-1660-1_S14_L001_R1_001.fastq.gz	gTip20TIAC-1660-1_S14_L001_R2_001.fastq.gz
SRR10201392	SAMN12871468	TIAC1660	Blood-TissueTIAC1660-TrisHCl	Blood-TissueTIAC1660-TrisHCl_S7_L001_R1_001.fastq.gz	Blood-TissueTIAC1660-TrisHCl_S7_L001_R2_001.fastq.gz
SRR10201491	SAMN12871468	TIAC1660	MCDPK-1660-buffer	MCDPK-1660-buffer_S17_L001_R1_001.fastq.gz	MCDPK-1660-buffer_S17_L001_R2_001.fastq.gz
SRR10201490	SAMN12871468	TIAC1660	MiniMAG-TIAC-1660-3	MiniMAG-TIAC-1660-3_S14_L001_R1_001.fastq.gz	MiniMAG-TIAC-1660-3_S14_L001_R2_001.fastq.gz
SRR10201489	SAMN12871468	TIAC1660	WGDPK-1660-buffer	WGDPK-1660-buffer_S4_L001_R1_001.fastq.gz	WGDPK-1660-buffer_S4_L001_R2_001.fastq.gz

The datasets supporting the conclusions of this study have been deposited in the NCBI SRA under the accession number PRJNA574887 (in-house sequenced data). The columns list the accession number of each sample, accession numbers of the seven samples, the sample name, the name of each library, and the filename of the forward and reverse read, respectively. The study accession number, the object status, and the bioproject accession numbers comprise SRP223655, "new", and PRJNA574887, respectively.

4. Supplementary Figures

4.1. Supplementary Fig. S1: Median mapping depths against Sakai *E. coli* O157:H7 reference genome and Sakai *E. coli* pO157 plasmid

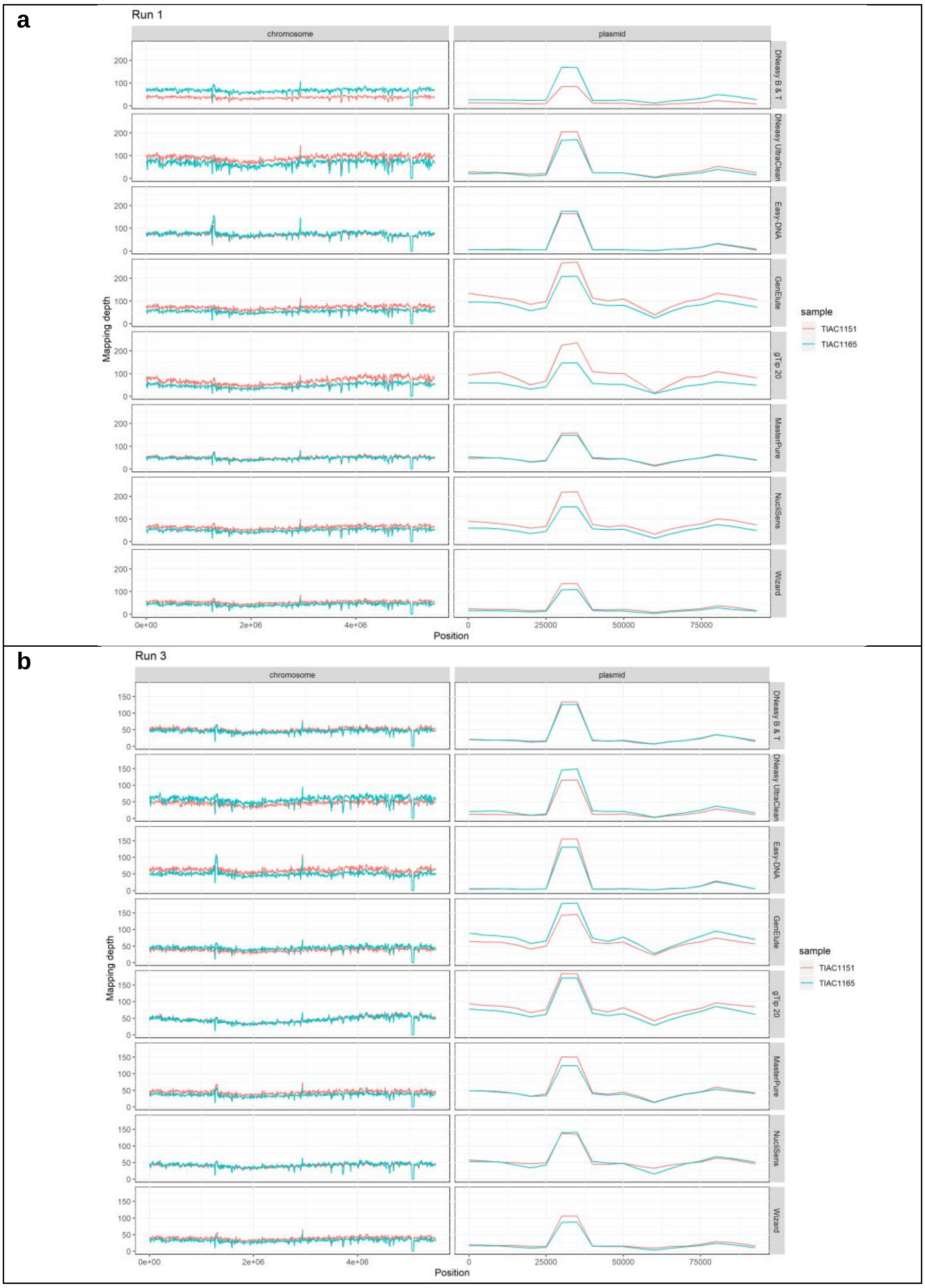

Supplementary Fig. S1 Overview of median mapping depths against the Sakai **E. coli** ***O157:H7 reference genome and the Sakai*** **E. coli** ***pO157 plasmid for sequencing replicates TIAC1151 and TIAC1165 for each kit, sequenced in run 1 (S1a) and run 3 (S1b).***

The median read mapping depth for each sample was calculated using a sliding window of 10,000 bases shifted by 5,000 bases for each data point.

Abbreviations: DNeasy Blood & Tissue kit (DNeasy B & T), DNeasy UltraClean Microbial kit (DNeasy UltraClean), Easy-DNA gDNA Purification kit (Easy-DNA), GenElute Bacterial gDNA kit (GenElute), Genomic-tip 20/G kit (gTip 20), MasterPure Complete DNA Purification kit (MasterPure), NucliSENS miniMag (NucliSens), Wizard gDNA Purification kit (Wizard).

4.2. Supplementary Fig. S2: %GC-content decrease in O-typing region of the Sakai *E. coli* O157:H7 reference genome

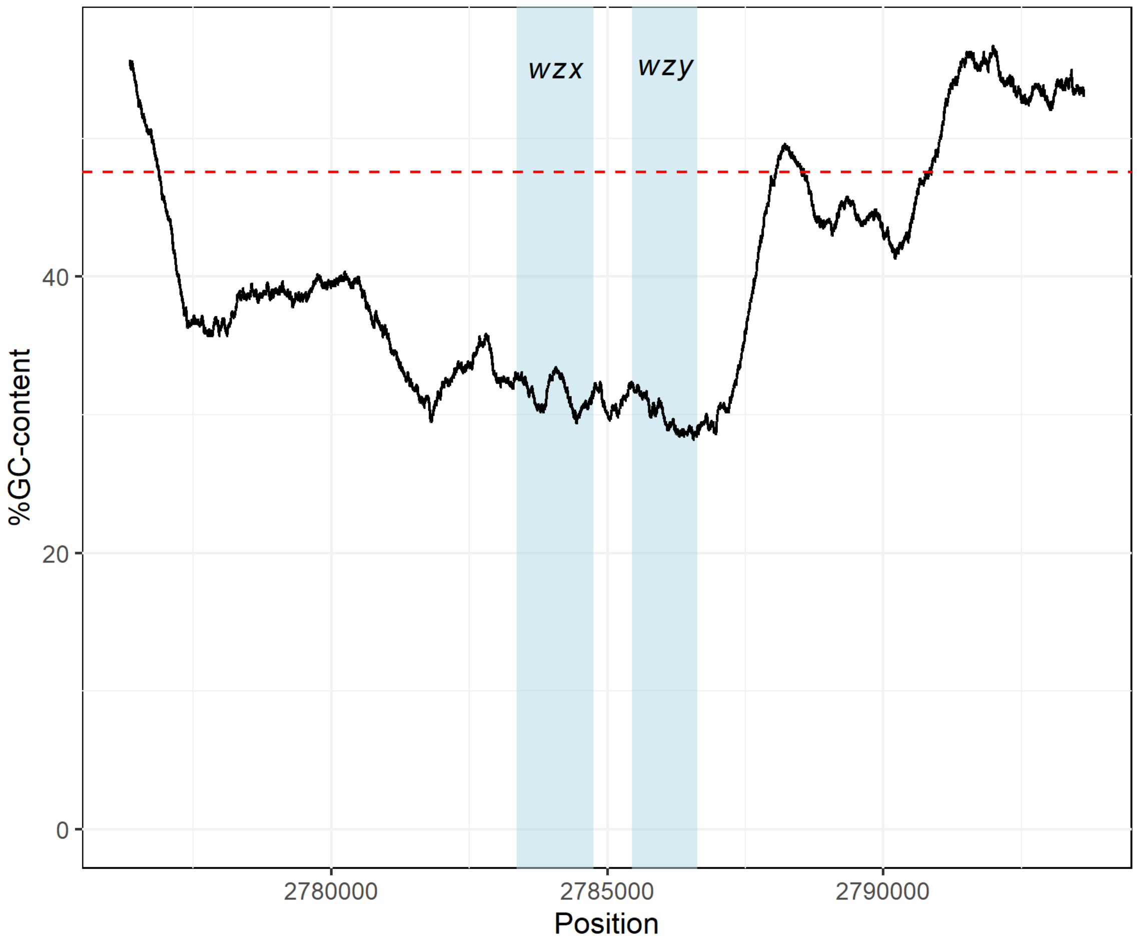

Supplementary Fig. S2 Decrease in %GC-content from position 2,775 kb to 2,795 kb in the Sakai* E. coli *O157:H7reference genome sequence.

The %GC-content over the chromosome of the Sakai E.coli *O157:H7 reference genome from position 2,775 kb to 2,795 kb is visualized; percentages were calculated over the 500 neighboring bases on each side. A decrease in %GC-content to ~30% in the region from 2,782,500 bp to 2,787,500 bp was observed. This region contains the O-antigen genes* wzx *and* wzy*, as marked in the figure. The dashed red line represents the average %GC-content of the entire reference sequence.*

4.3. Supplementary Fig. S3: Overview of virulence genotype obtained per sequencing run replicate and EDTA-containing samples

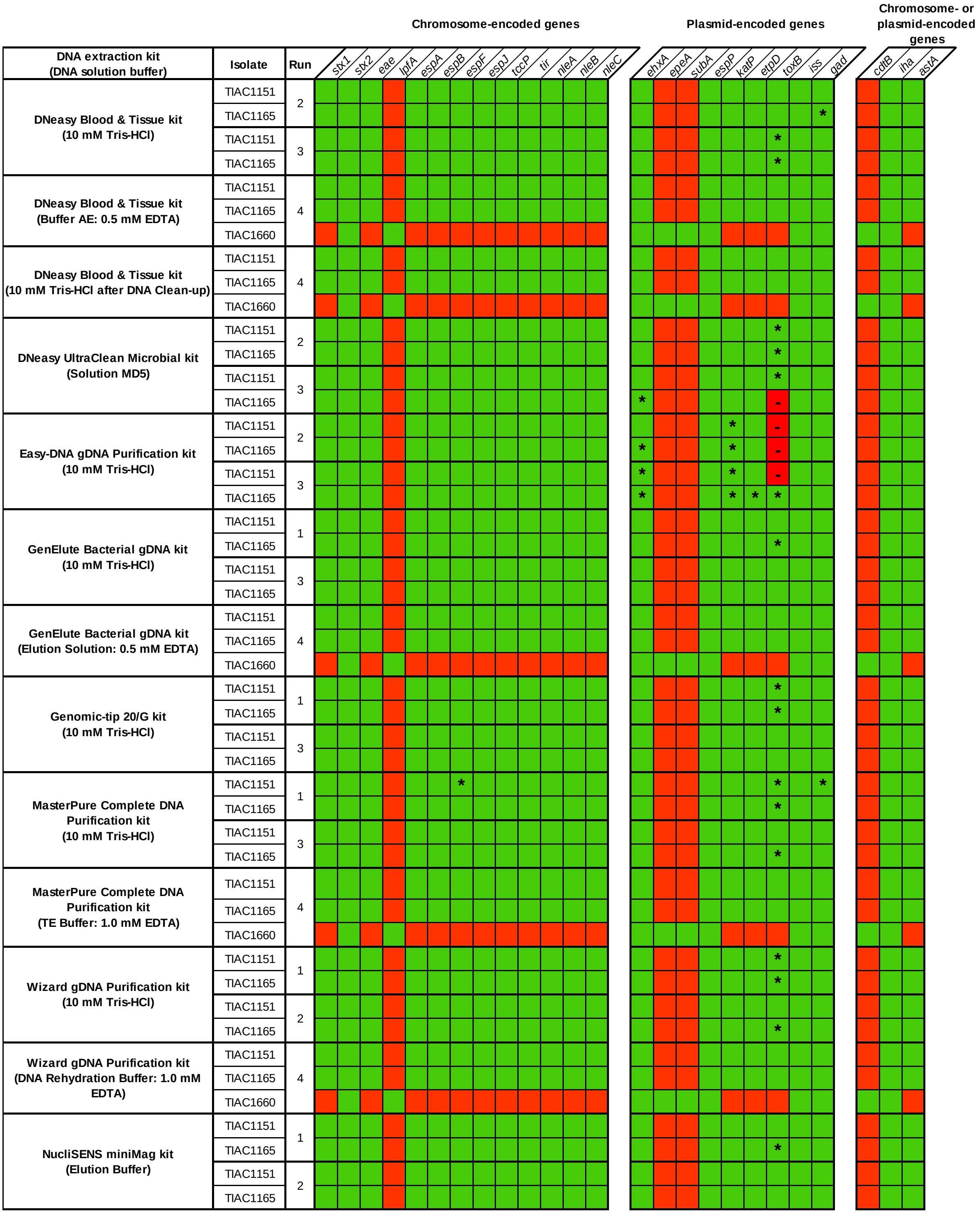

Supplementary Fig. S3 Overview of the virulence genotype obtained per sequencing run replicate and EDTA-containing samples

Presence or absence of virulence genes determined using BLAST+ and SRST2 (using thresholds of 60 % query coverage and 90 % sequence identity with the reference gene for gene detection) are indicated in green or red, respectively. Results are shown per kit for each sequencing run replicate and for the isolates from which the DNA was prepared with the four EDTA containing DNA extraction kits and for which the DNA was eluted or rehydrated in the supplied buffer containing EDTA.

**Virulence genes only detected with SRST2 read mapping*

-Missed virulence genes (not detected with SRST2, nor BLAST+)

4.4. Supplementary Fig. S4: %GC-content decrease in *toxB*-region of the Sakai *E. coli* O157:H7 plasmid reference genome

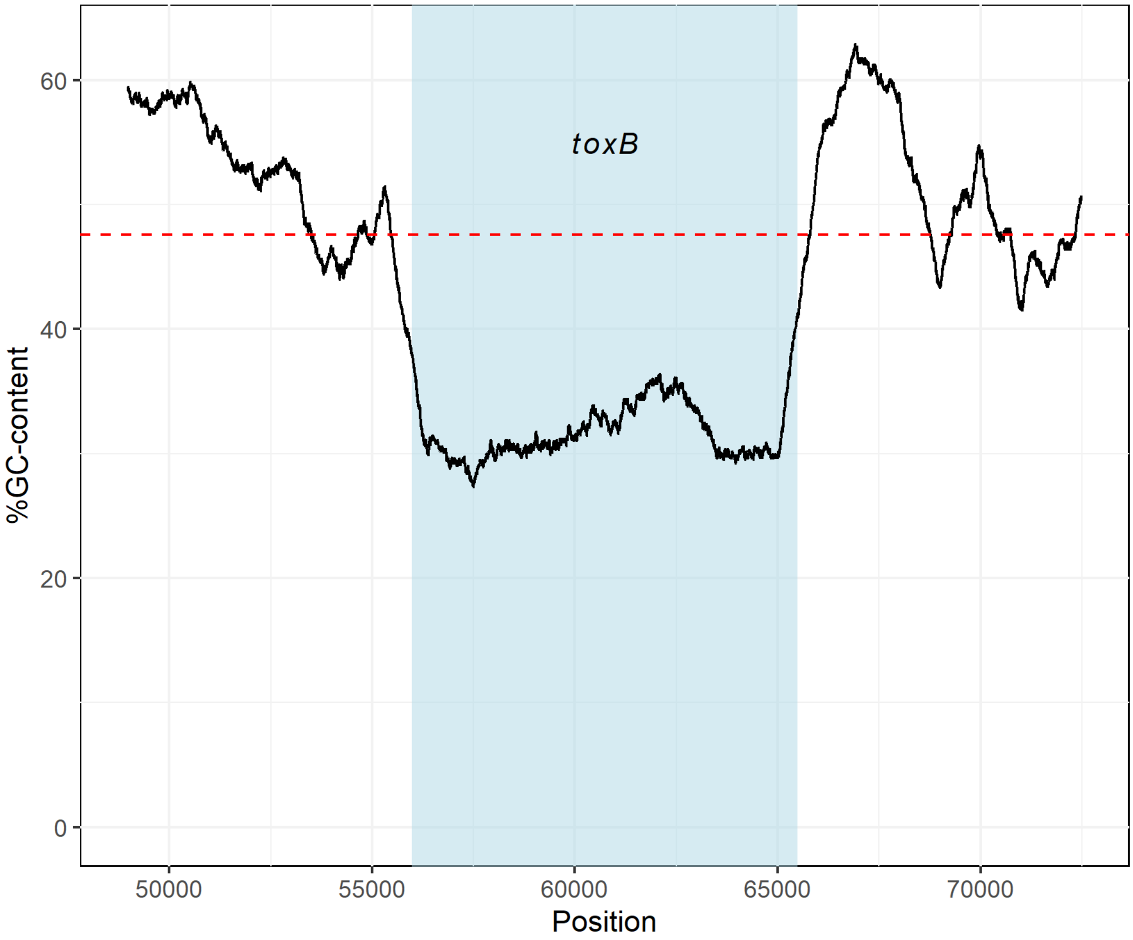

Supplementary Fig. S4 Decrease in %GC-content from position 50 kb to 70 kb in the Sakai* E. coli *pO157 plasmid sequence.

The %GC-content over the chromosome of the Sakai E. coli *O157:H7 reference plasmid from position 50 kb to 70 kb is visualized. Percentages were calculated over the 500 neighboring bases on each side. A decrease in %GC-content to ~30% was observed from position ~55.5 kb to ~65.5 kb. This region contains the* toxB *gene, as marked in the figure. The red dashed line represents the average %GC-content of the full plasmid sequence.*

4.5. Supplementary Fig. S5: cgMLST tree containing all samples, sequencing run replicates and EDTA-containing samples

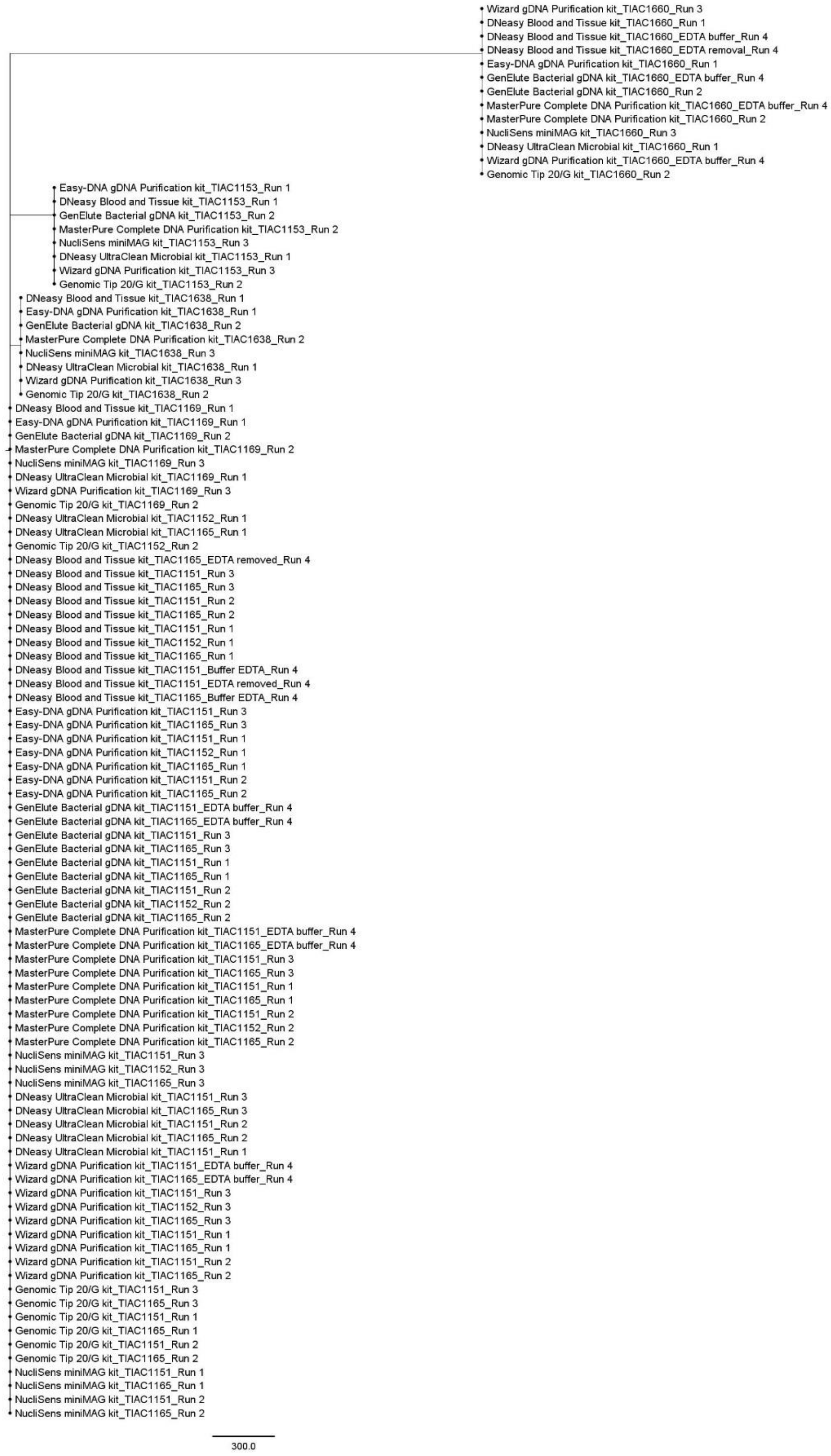

Supplementary Fig. S5 cgMLST tree containing all samples, sequencing run replicates and EDTA-containing samples

A minimum spanning tree was created with the MSTreeV2 method. The cgMLST tree was generated of all samples, sequencing run replicates and the isolates from which the DNA was eluted in the EDTA-containing buffer of the DNeasy Blood & Tissue, GenElute Bacterial gDNA, MasterPure Complete DNA Purification and Wizard gDNA Purification kits. All outbreak isolates (TIAC1151, TIAC1152, TIAC1165, TIAC1169) consistently clustered together, while non-outbreak samples TIAC1153, TIAC1638 and TIAC1660 were separated from the outbreak cluster. The scale bar represents number of cgMLST allele differences between samples.

Abbreviations: DNeasy Blood & Tissue kit (DNeasy Blood and Tissue kit), DNeasy UltraClean Microbial kit (DNeasy UltraClean Microbial kit), Easy-DNA gDNA Purification kit (Easy-DNA gDNA Purification kit), GenElute Bacterial gDNA kit (GenElute Bacterial gDNA kit), Genomic-tip 20/G kit (Genomic Tip 20/G kit), MasterPure Complete DNA Purification kit (MasterPure Complete DNA Purification kit), NucliSENS miniMag (NucliSens miniMAG kit), Wizard gDNA Purification kit (Wizard gDNA Purification kit).

4.6. Supplementary Fig. S6: SNP-tree containing all samples, sequencing run replicates and EDTA-containing samples

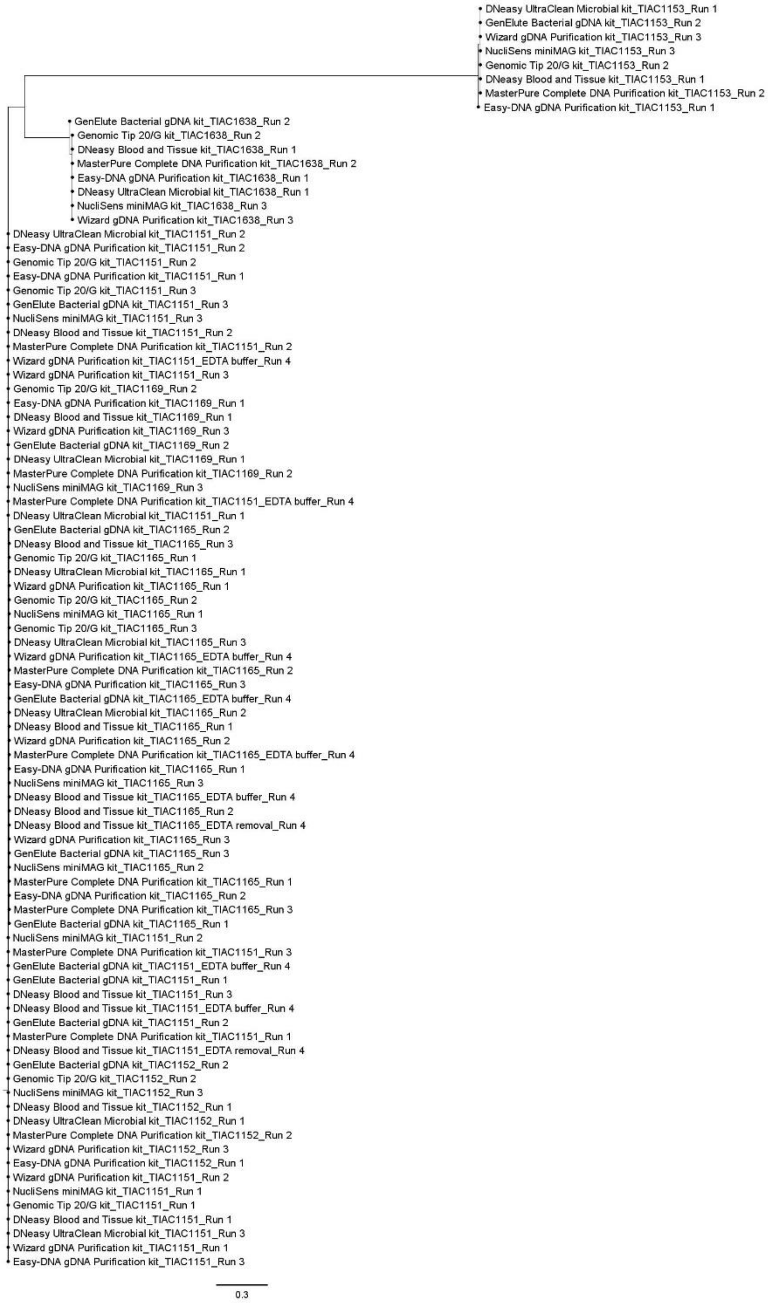

Supplementary Fig. S6 SNP tree containing all samples, sequencing run replicates and EDTA-containing samples
A maximum likelihood SNP tree was generated using the K2 nucleotide substitution model for the six E. coli *O157:H7 samples and all sequencing run replicates of the eight kits, and the isolates from which the DNA was eluted in the EDTA-containing buffer of the DNeasy Blood & Tissue, GenElute Bacterial gDNA, MasterPure Complete DNA Purification and Wizard gDNA Purification*

kits. All outbreak samples (TIAC1151, TIAC1152, TIAC1165, and TIAC1169) and their sequencing run replicates consistently clustered together, irrespective of the employed kit and the presence of EDTA in the DNA solution buffer. Interestingly, within the outbreak clade, specifically in the TIAC1165 samples, a limited number of SNPs different with the reference genome between the other outbreak samples was observed. The non-outbreak samples (TIAC1153 and TIAC1638) were separated from the outbreak clade, and clustered together per isolate. Notably, for isolate TIAC1153, the small number of SNPs different with the reference genome between the sample generated with the Easy-DNA gDNA Purification kit and all other TIAC1153 samples, was observed. The same applied for the TIAC1638 isolate processed with the GenElute Bacterial gDNA kit. These differences were solely due to masking of a low-quality region (see results).The distance scale bar represents the average number of nucleotide substitutions per site.

Abbreviations: DNeasy Blood & Tissue kit (DNeasy Blood and Tissue kit), DNeasy UltraClean Microbial kit (DNeasy UltraClean Microbial kit), Easy-DNA gDNA Purification kit (Easy-DNA gDNA Purification kit), GenElute Bacterial gDNA kit (GenElute Bacterial gDNA kit), Genomic-tip 20/G kit (Genomic Tip 20/G kit), MasterPure Complete DNA Purification kit (MasterPure Complete DNA Purification kit), NucliSENS miniMag (NucliSens miniMAG kit), Wizard gDNA Purification kit (Wizard gDNA Purification kit).

4.7. Supplementary Fig. S7: Influence of EDTA on Nextera XT library preparation

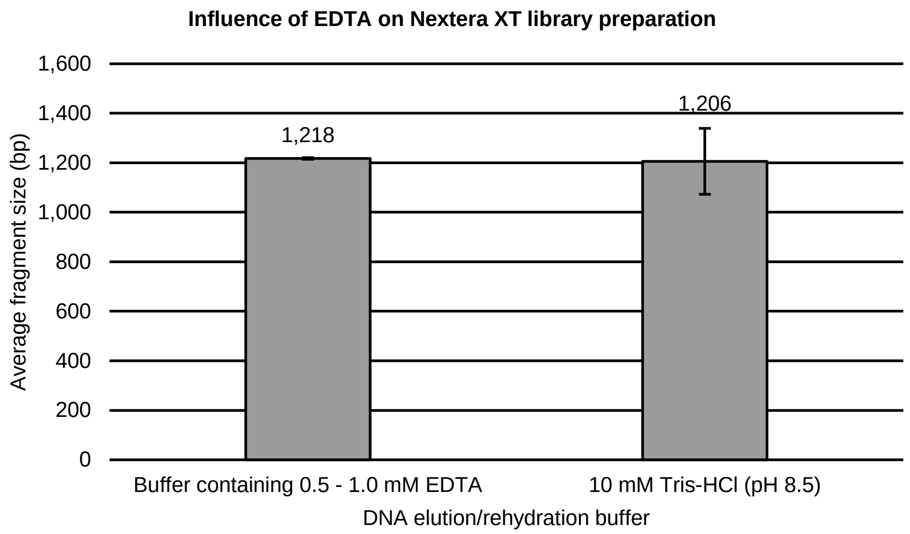

Supplementary Fig. S7 Influence of EDTA on DNA fragmentation during library preparation

The influence of EDTA in DNA solution buffers of the respective four DNA extraction kits on the DNA fragmentation step during Nextera XT (Illumina, San Diego, CA) library preparation was investigated through comparison of the average length of the DNA fragments solubilized in the buffer containing EDTA and the 10 mM Tris-HCl (pH 8.5) buffer. The average fragment sizes were calculated from three DNA extracts (TIAC1151, TIAC1165, and TIAC1660) eluted in the buffer containing 0.5 mM or 1.0 mM EDTA, or in 10 mM Tris-HCl (pH 8.5). Standard deviations are shown with the error bars.

4.8. Supplementary Fig. S8: Influence of EDTA on average number of reads uniquely mapping to the Sakai *E. coli* O157:H7 reference per million input reads

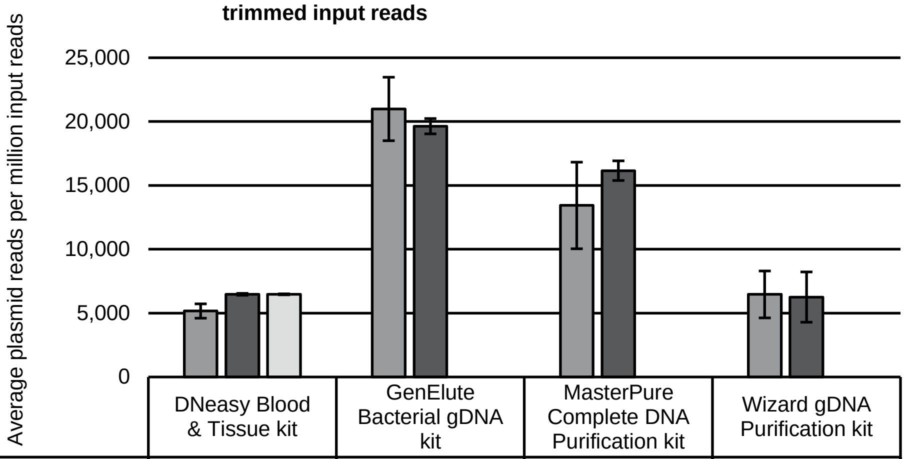

	DNeasy Blood & Tissue kit	GenElute Bacterial gDNA kit	MasterPure Complete DNA Purification kit	Wizard gDNA Purification kit
10 mM Tris-HCl (pH 8.5)	5,167	20,990	13,433	6,465
DNA solution buffer (EDTA)	6,475	19,640	16,157	6,259
DNA Clean & Concentrator kit (EDTA removal)	6,475			

Supplementary Fig. S8 The effect of EDTA on the average number of reads mapping uniquely to the Sakai* E. coli *pO157 plasmid reference normalized per one million trimmed input reads for the four kits containing EDTA in the DNA solution buffer.

Number of reads mapping uniquely against the Sakai E. coli *pO157 plasmid reference per million input reads when mapping simultaneously against the Sakai* E. coli *pO157 plasmid (NC_002128.1) and Sakai* E. coli *O157:H7 chromosomal (NC_002695.2) reference. This was performed for two samples (TIAC1151 and TIAC1165) that have been processed with the DNeasy Blood & Tissue, GenElute Bacterial gDNA, MasterPure Complete DNA Purification, and Wizard gDNA Purification kits and eluted in the accompanying elution/rehydration buffer containing 0.5 mM or 1.0 mM EDTA. Values were averaged over both samples that were processed with the four kits, per kit. The obtained average number of reads per kit mapping uniquely to the Sakai* E. coli *pO157 plasmid reference per one million trimmed input reads were compared to the average number of reads mapping uniquely to the Sakai* E. coli *pO157 plasmid reference per one million trimmed input reads of all O157:H7 samples (TIAC1151, TIAC1152, TIAC1153, TIAC1165, TIAC1169, and TIAC1638) processed with the four same kits, but from which the DNA was eluted/rehydrated in 10 mM Tris-HCl (pH 8.5). For the DNeasy Blood & Tissue kit (in the Fig. mentioned as Dneasy Blood and Tissue kit), the comparison was also made with the average number of reads mapping uniquely to the Sakai* E. coli *pO157 plasmid reference per one million trimmed input reads of the two samples TIAC1151 and TIAC1165, from which EDTA removal was performed using the DNA Clean & Concentrator kit after DNA elution in the 0.5 mM EDTA-containing buffer. Bars represent the standard deviation across samples for each extraction kit.*

5. References

1. Lecker, D. N. & Khan, A. Theoretical and experimental studies of the effects of heat, EDTA, and enzyme concentration on the inactivation rate of α-amylase from *Bacillus* sp. *Biotechnol. Prog.* **12**, 713–717 (1996).
2. Lovell, S., Goryshin, I. Y., Reznikoff, W. R. & Rayment, I. Two-metal active site binding of a Tn5 transposase synaptic complex. *Nat. Struct. Biol.* **9**, 278–281 (2002).
3. Meinke, W. J. & Jones, L. A. Lytic enzyme from lysates of *Streptomyces venezuelae* infected with actinophage MSP2. *J. Bacteriol.* **106**, 386–393 (1971).
4. Nielsen, E. M. & Andersen, M. T. Detection and characterization of verocytotoxin-producing *Escherichia coli* by automated 5′ nuclease PCR assay. *J. Clin. Microbiol.* **41**, 2884–2893 (2003).
5. Barbau-Piednoir, E. *et al.* Detection and discrimination in food samples of five *E. coli* pathotypes using a Combinatory SYBR®Green qPCR screening system. *Prep.* 3267–3285 (2015).
